# Zinc-Catalyzed Hydroboration of Carbon Dioxide Amplified by Borane-Tethered Heteroscorpionate Bis(Pyrazolyl)methane Ligands

**DOI:** 10.1021/acs.inorgchem.4c00500

**Published:** 2024-04-24

**Authors:** Tiago F. C. Cruz, Valentin Loupy, Luís F. Veiros

**Affiliations:** Centro de Química Estrutural, Institute of Molecular Sciences, Departamento de Engenharia Química, Instituto Superior Técnico, Universidade de Lisboa, Av. Rovisco Pais, 1049 001 Lisboa, Portugal

## Abstract

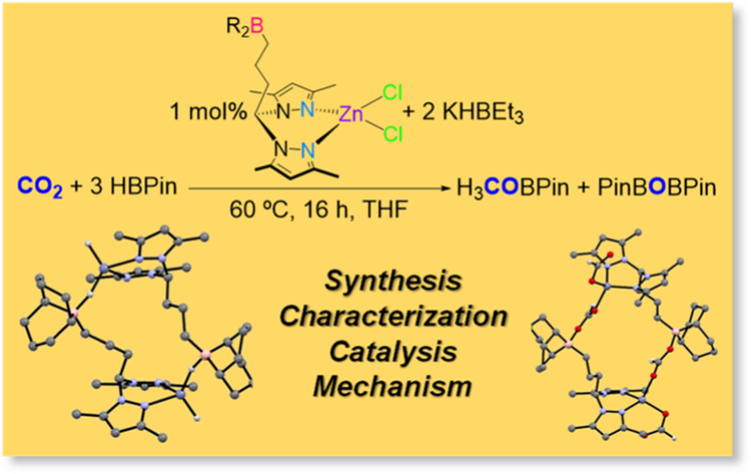

The borane-functionalized (BR_2_) bis(3,5-dimethylpyrazolyl)methane (**L**_**H**_) ligands **1a** (BR_2_: 9-borabicyclo[3.3.1]nonane or 9-BBN), **1b** (BR_2_: BCy_2_), and **1c** (BR_2_: B(C_6_F_5_)_2_) were synthesized by the allylation–hydroboration of **L**_**H**_. Metalation of **1a,b** with ZnCl_2_ yielded the heteroscorpionate dichloride complexes **[(1a,b)ZnCl**_**2**_**] 3a,b**. The reaction of **1a** with ZnEt_2_ led to the formation of the zwitterionic complex **[Et(1a)ZnEt(THF)] 5**. The reaction of complex **3a** with two equivalents of KHBEt_3_ under a carbon dioxide (CO_2_) atmosphere gave rise to the formation of the dimeric bis(formate) complex **[(1a)Zn(OCHO)**_**2**_**]**_**2**_**8**, in which its borane moieties intermolecularly stabilize the formate ligands of opposite metal centers. The allylated precursor **L**_**allyl**_ and its zinc dichloride, diethyl and bis(formate) complexes **[(L**_**allyl**_**)ZnCl**_**2**_**] 2**, **[(L**_**allyl**_**)ZnEt**_**2**_**] 4**, and **[(L**_**allyl**_**)Zn(OCHO)**_**2**_**] 7** were also isolated. The catalyst systems composed of 1 mol % of **3a** or **3b** and two equivalents of KHBEt_3_ hydroborated CO_2_ at 1 bar with pinacolborane (HBPin) to the methanol-level product H_3_COBPin (and PinBOBPin) in yields of 42 or 86%, respectively. The catalyst systems using the unfunctionalized complex **[(L**_**H**_**)ZnCl**_**2**_**] 6** and KHBEt_3_ or KHBEt_3_/*n*OctBR_2_ (BR_2_: 9-BBN or BCy_2_) hydroborated CO_2_ to H_3_COBPin but in 2.5- to 6-fold lower activities than those exhibited by **3a,b**/KHBEt_3_. The hydroboration of CO_2_ using **8** as a catalyst led to yields of 39–43%, comparable to those obtained with **3a**/KHBEt_3_. The results confirmed that the catalytic intermediates benefit from the incorporated boranes’ intra- or intermolecular stabilizations.

## Abstract 2

New heteroscorpionate zinc complexes bearing borane-tethered bis(3,5-dimethylpyrazolyl)methane ligands served as precatalysts for the hydroboration of CO_2_ with pinacolborane (HBPin) to the methanol-level product H_3_COBPin. Experimental and theoretical observations confirmed that the catalytic intermediates benefit from the incorporated boranes’ intra- or intermolecular stabilizations.

## Introduction

Atmospheric greenhouse gas buildup has been an undesired consequence of industrialization with serious ecosystem implications. Carbon dioxide (CO_2_) constitutes 80% of the total accumulated atmospheric gases,^1^ and its mitigation is currently a priority to counteract the effects of climate change.^2^ Physicochemical CO_2_ trapping technologies are attractive^3^ but present low efficiency and high operation costs.^4^ Chemically, the transformation of CO_2_ by copolymerization with epoxides^5^ or reaction with suitable substrates,^6^ for instance, has led to the synthesis of polycarbonates or formulations of pharmacological interest. These transformations usually require the use of catalytic processes.

Catalysts capable of yielding C1 building blocks from CO_2_ usually rely on dihydrogen gas (H_2_) as the hydrogen source and are mostly of heterogeneous nature.^7^ However, heterogeneous catalysts and H_2_ gas often require harsh operation conditions and present low activity/selectivity. Homogeneous-catalyzed hydrofunctionalization has emerged as a good strategy to chemically transform CO_2_ to C1 building blocks, by using mild conditions and inexpensive reductants, such as boranes (hydroboration)^8,9^ or silanes (hydrosilylation)^10^ as hydrogen sources.

**Chart 1:**
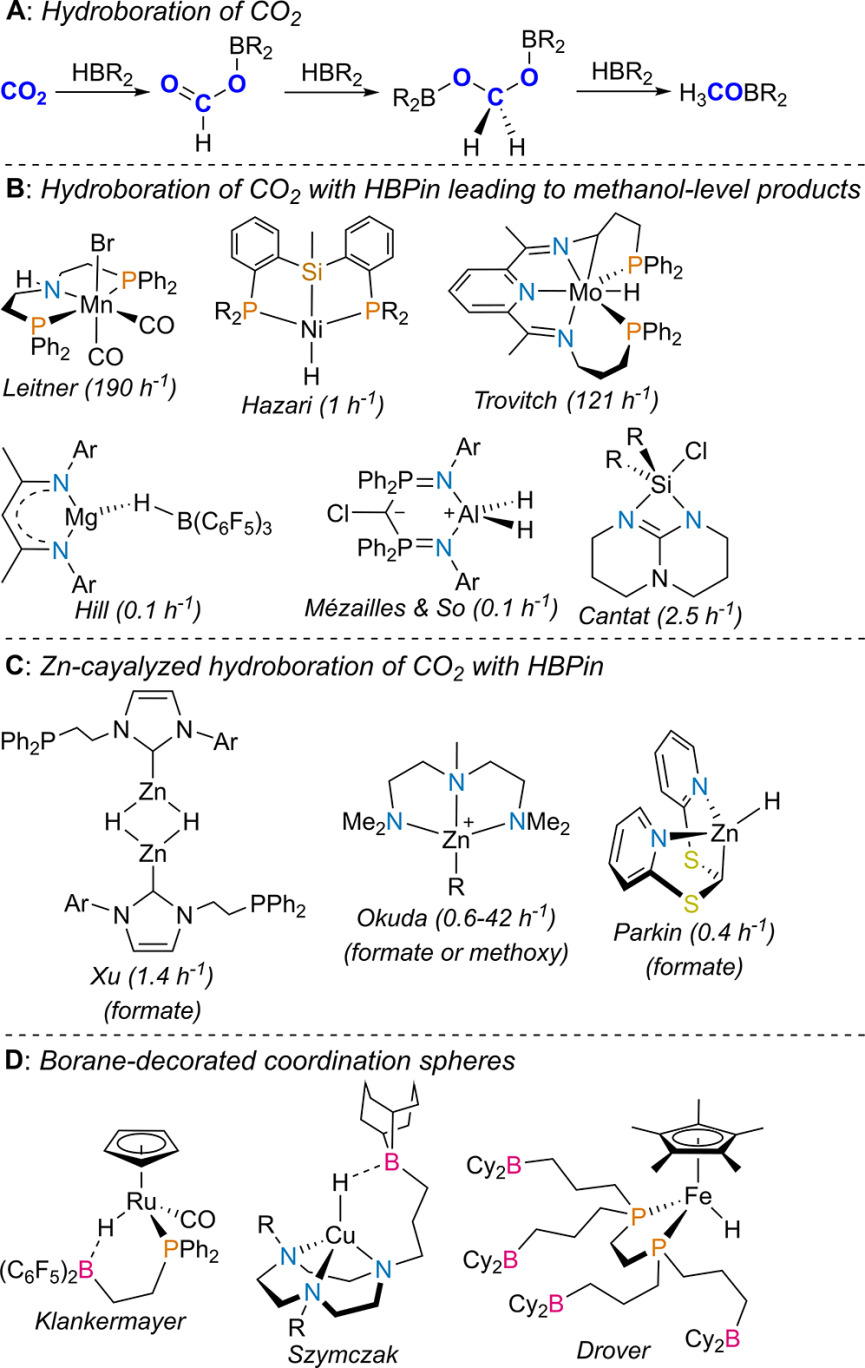
Different Levels of CO_2_ Reduction by Hydroboration (A), Selection of Catalysts for the Hydroboration of CO_2_ with HBPin to Methanol-Level Products (B), Zn-Based Catalysts for the Hydroboration of CO_2_ with HBPin (C), and Examples of Borane-Functionalized Coordination Spheres (D)a

CO_2_ hydroboration may lead to different levels of chemical reduction depending on the catalyst used; formate, methylene, and methoxy boranes are among the possible products (Chart 1, A). The first example of catalytic CO_2_ hydroboration with pinacolborane (HBPin) was reported by Sabo-Etienne and co-workers and used the dihydride bis(dihydrogen) ruthenium complex [RuH_2_(H_2_)_2_(PCy_3_)_2_], where mixtures of different products were obtained.^11^ Since then, the hydroboration of CO_2_ with HBPin has been catalyzed by a variety of molecular systems. Transition metals, such as Mn,^12^ Fe,^13^ Co,^14^ Ni,^15^ Cu,^16^ Mo,^17^ Ru,^18^ and Pd,^19^ and main group elements^20^ have been used in catalyst systems for CO_2_ hydroboration with HBPin, and their catalytic activity often originating from cooperativity between Lewis pairs.^21^ Other boranes, such as the 9-H-9-borabicyclo[3.3.1]nonane (9-BBN) dimer, catecholborane (HBCat), and BH_3_, have been known to hydroborate CO_2_, barring catalytic activation.^22^ Among the catalysts used in CO_2_ hydroboration with HBPin, only selected cases have been known to selectively generate the methoxy product H_3_COBPin (Chart 1, B).^23^ Zinc-catalyzed hydroboration of CO_2_ with HBPin has been previously reported, the formate and methoxy products being obtained (Chart 1, C).^24^

a The values in parentheses display the turnover frequencies of the catalysts and/or respective products.

Catalytic CO_2_ reduction depends on the effective and reversible activation of an M–H bond en route to active M–OCHO species. The activation of otherwise catalytically inactive hydride metal complexes by strong Lewis acidic boranes generating the respective cationic complexes has been successful in achieving such reactivity requirements.^25^ This approach has been adopted by promoting cooperation between the Lewis acids B(C_6_F_5_)_3_ and Al(C_6_F_5_)_3_. This combination successfully hydrosilylated CO_2_ to methane by promoting M–OCHO–SiR_3_ (M = B, Al) interactions.^26^ In particular, Zn–L–borane (L = H, OCHO) interactions led to enhanced stability of the respective complexes and promoted the disaggregation of potential multinuclear entities that would otherwise occur in unprotected Zn–L complexes.^27^

Inspired by these findings, we envisioned that manipulating the secondary coordination sphere of a metal hydride (or formate) complex by intramolecularly incorporating a strong Lewis acid would lead to efficient CO_2_ reduction. Secondary coordination sphere manipulation is pushing the boundaries of chemistry research, particularly toward homogeneous catalysis.^28^ Decorating established coordination complexes with boranes has been explored by a few research groups (Chart 1, D). Bercaw and co-workers used a borane–phosphine ligand to activate carbonyl ligands in rhenium complexes,^29^ while Klankermayer used the same ligand to activate Ru–H bonds.^30^ Szymczak and co-workers presented several works containing transition metal complexes of multidentate nitrogen-based ligands, based on pyridinepyrazolyl or 1,4,7-triazacyclononane derivatives functionalized with boranes, with applications in the stoichiometric activation of small molecules.^31^ Drover and co-workers prepared late-transition metal complexes of borane-functionalized 1,2-bis(phosphino)ethanes.^32^ Werlé and co-workers reported catalysts based on a borane-functionalized phosphinotriazine chelate for the hydrogenation of nitroarenes or the synthesis of silyl enol ethers.^33^

We have turned to the bis(pyrazolyl)methane (bpm) scaffold because of the feasibility of functionalizing the respective methylenic carbon and anticipating that such function would likely stand in an axial position with respect to the metal center. The bpm framework^34^ is the much less studied analogue of the *tris*(pyrazolyl)methane derivatives, also known as scorpionate ligands, widely used in the coordination chemistry field.^35^ The functionalization of bpm at the methylenic carbon has been featured in the literature, giving rise to heteroscorpionates with catalytic^36^ or medicinal applications.^37^ Flores and co-workers have successfully alkylated the bpm moiety at the methylenic carbon, in order to prepare dendritic Ni(II) complexes.^38^ McSkimming also prepared iron and cobalt complexes of bpm precursors containing an alkyl donor.^39^ Functionalization of the methylenic carbon of the bpm moiety with acetamidinate^40^ or phosphinimide^41^ moieties has also been successfully used in the preparation of zinc complexes, with applications in ring-opening polymerization reactions.

In this work, we have functionalized bis(3,5-dimethylpyrazolyl)methane (**L**_**H**_)^42^ with different boranes and prepared the respective bifunctional zinc complexes. Taking advantage of our experience in catalytic hydroboration of carbonyl groups,^43^ the new zinc complexes were used as precatalysts in the hydroboration of CO_2_ with HBPin to yield the methanol-level product H_3_COBPin and have explored the role of the newly included boranes in the catalytic activity and mechanism.

## Results and Discussion

### Synthesis and Characterization of the Ligands and Complexes

**Scheme 1:**
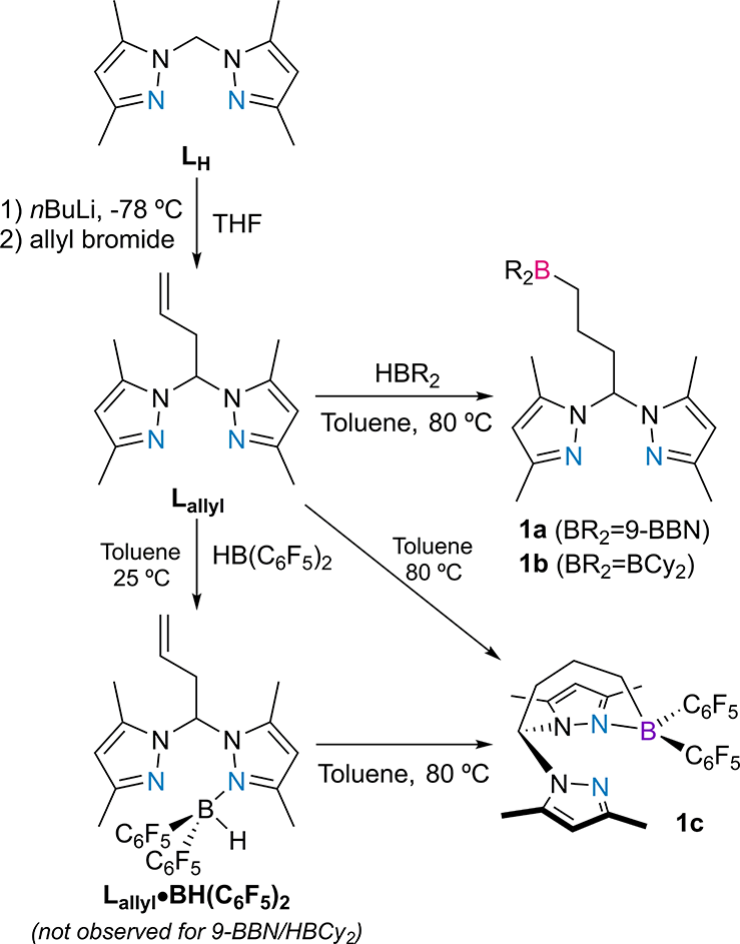
Synthesis of the Ligands **L**_**allyl**_ and **1a**–**c** and Detection of the Adduct **L**_**allyl**_·**HB(C**_**6**_**F**_**5**_**)**_**2**_

In order to functionalize **L**_**H**_ with pending boranes, an allylation–hydroboration methodology was used. First, **L**_**H**_ was *in situ* lithiated at the methylenic carbon with *n*-butyllithium at −78 °C, followed by a reaction with allyl bromide giving rise to the allylated derivative 1,1′-(but-3-ene-1,1-diyl)bis(3,5-dimethylpyrazolyl) **L**_**allyl**_ in moderate yields, by adapting similar literature procedures.^37,38^ Subsequently, the reaction of **L**_**allyl**_ with the 9-BBN dimer, dicyclohexylborane (HBCy_2_) or Piers’ borane (HB(C_6_F_5_)_2_) in toluene at 80 °C for 1 h, respectively, gave rise to the heteroscorpionate bis(3,5-dimethylpyrazolyl)methane compounds with borane (BR_2_) functionalities **1a** (BR_2_: 9-BBN), **1b** (BR_2_: BCy_2_), and **1c** (BR_2_: B(C_6_F_5_)_2_) in good yields, *viaanti*-Markovnikov hydroboration reactions (Scheme 1).

When **L**_**allyl**_ was reacted with HB(C_6_F_5_)_2_ in toluene at 25 °C, the Lewis pair **L**_**allyl**_·**HB(C**_**6**_**F**_**5**_**)**_**2**_ was formed instead, which completely converted into **1c** upon heating to 80 °C. Similar Lewis adducts were never observed in reactions using the 9-BBN dimer or HBCy_2_. Attempts to shorten the hydrocarbyl chain in **L**_**allyl**_ or **1a** by *in situ* lithiation of **L**_**H**_ followed by reaction with vinyl bromide or BrCH_2_CH_2_(9-BBN), respectively, were unsuccessful. The new compounds were characterized by NMR spectroscopy and elemental analysis.

The NMR spectra of **L**_**allyl**_ (Figures S1 and S2 in the Supporting Information (SI)) present the expected resonances for an allyl-functionalized bis(3,5-dimethylpyrazolyl)methyl moiety, and the methine proton resonance appears as a triplet at 6.25 ppm, coupling with the methylenic allyl resonance, which appears as another triplet at 3.34 ppm (^3^*J*_HH_ = 9.0 Hz). The NMR spectra of compounds **1a,b** (Figures S3–S8 in the SI) also present the resonances associated with bis(3,5-dimethylpyrazolyl)methane and 9-BBN or BCy_2_ moieties; the methine ^1^H NMR resonance appears as a triplet at 6.29 ppm coupling with a methylenic ^1^H resonance at 2.61 ppm (^3^*J*_HH_ = 9.0 Hz). The *anti*-Markovnikov additions to **L**_**allyl**_ in compounds **1a,b** are evidenced by a succession of three coupled methylenic proton resonances, centered at 2.58–2.61, 1.61–1.63, and 1.44–1.45 ppm. The methyl ^1^H NMR resonances of **L**_**allyl**_ and **1a,b** appear at 2.19 ppm, indicating rapid rotations of the 3,5-dimethylpyrazolyl rings about the methinic carbon–nitrogen bonds. The ^11^B NMR resonances of compounds **1a,b** appear in the range of 83.8–88.5 ppm, characteristic of tricoordinate boranes simultaneously containing alkyl and 9-BBN/BCy_2_ motifs.^44^ On the other hand, the NMR spectra of compounds **L**_**allyl**_·**HB(C**_**6**_**F**_**5**_**)**_**2**_ and **1c** (Figures S9–S16 in the SI) presented the spectroscopic features of asymmetric bis(3,5-dimethylpyrazolyl)methane moieties, respectively, containing allyl or hydroborated functionalities, caused by the fact that one of the two pyrazolyl rings formed Lewis pairs with HB(C_6_F_5_)_2_ or intramolecular B(C_6_F_5_)_2_ fragments. The asymmetry in compounds **L**_**allyl**_·**HB(C**_**6**_**F**_**5**_**)**_**2**_ and **1c** is verified by the presence of two different 3,5-dimethylpyrazolyl CH and diastereotopic CH_2_ proton resonances as well as four different methyl ^1^H/^13^C NMR resonances. The ^11^B NMR spectra of **L**_**allyl**_·**HB(C**_**6**_**F**_**5**_**)**_**2**_ and **1c** possess resonances at −15 and −4 ppm, respectively, characteristic of tetracoordinate boron compounds containing two C_6_F_5_ groups.^45^

**Scheme 2:**
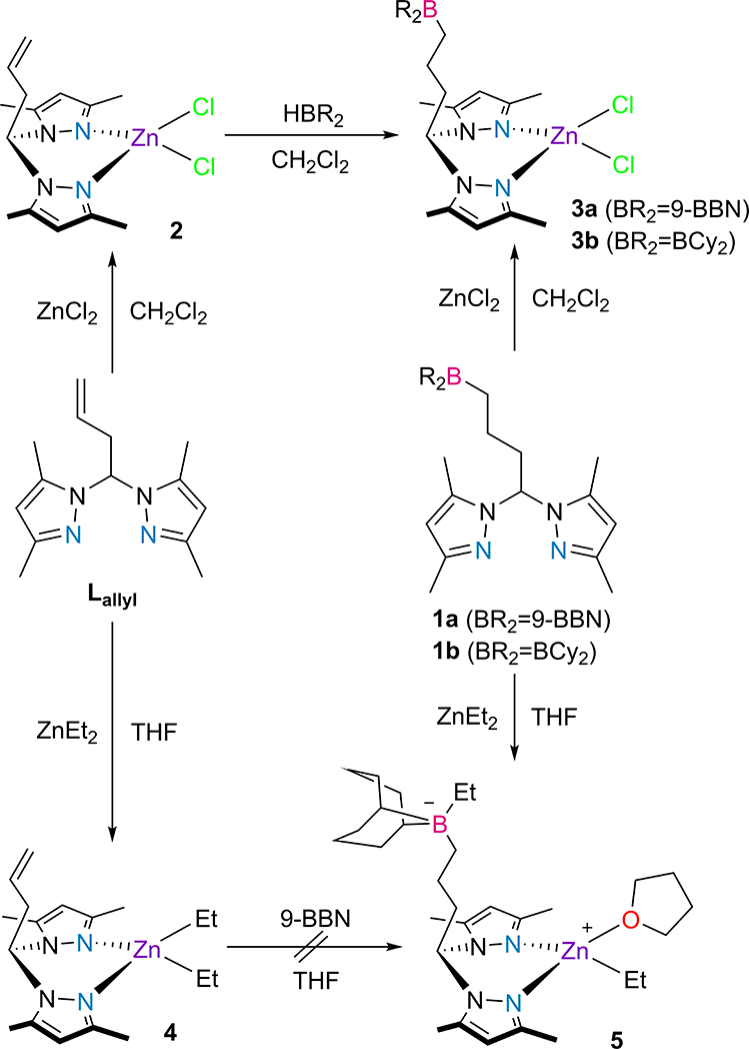
Synthesis of the Zinc Dichloride (**2**, **3a**, and **3b**) and Diethylzinc (**4** and **5**) Complexes

The heteroscorpionate zinc dichloride complexes **[(L**_**allyl**_**)ZnCl**_**2**_**] 2**, **[(1a)ZnCl**_**2**_**] 3a** and **[(1b)ZnCl**_**2**_**] 3b** were synthesized in good yields, *via* metalation of **L**_**allyl**_, **1a**, and **1b**, respectively, with ZnCl_2_ in dichloromethane (Scheme 2).

Metalation of **L**_**allyl**_ with ZnEt_2_ in THF afforded the heteroscorpionate diethylzinc complex **[(L**_**allyl**_**)ZnEt**_**2**_**] 4**. By its turn, the reaction of **1a** with ZnEt_2_ led to the formation of the zwitterionic complex **[Et(1a)ZnEt(THF)] 5**, in which it is presumed that the 9-BBN moiety abstracted an ethyl anion from the newly formed zinc center, which, in turn, is stabilized by a coordinated THF molecule.

Complexes **3a** and **3b** could also be isolated in good yields *via* post *anti*-Markovnikov hydroboration of complex **2** with an excess of the 9-BBN dimer or HBCy_2_, respectively, in dichloromethane, for 16 h at 25 °C. The zinc dichloride complex of **L**_**H**_ (complex **[(L**_**H**_**)ZnCl**_**2**_**] 6**), which had been previously reported in the literature,^46^ was also analogously synthesized for comparison purposes. The reaction of complex **4** with the half equivalent of the 9-BBN dimer did not lead to the formation of complex **5**, with an uncharacterizable mixture being instead isolated. Metalation of **1c** with ZnCl_2_ or reaction of complex **2** with HB(C_6_F_5_)_2_ did lead to a new zinc dichloride complex (NMR spectra of a reaction of **1c** with ZnCl_2_ in Figures S25–S27 in the SI), whose impure nature, despite recrystallization attempts, precluded further characterization and use. The reaction of compound **1c** with ZnEt_2_ or post hydroboration of complex **4** with HB(C_6_F_5_)_2_ led to untraceable mixtures. The lack of success in complexation reactions of **1c** was likely due to the occurrence of side reactions associated with the very high Lewis acidity of its B(C_6_F_5_)_2_ fragment.

All new complexes were characterized by NMR and Fourier transform infrared (FTIR) spectroscopies and elemental analysis (except for complexes **4** and **5**, due to their great air and recrystallization instability). The NMR spectra of complexes **2**–**5** (Figures S17–S24 and S28–S32 in the SI) display the expected resonances for the moieties present in **L**_**allyl**_ and **1a,b**, and complexes **3a,b** show ^11^B NMR resonances in the range of 84.2–88.2 ppm. The CH resonance of the 3,5-dimethylpyrazolyl rings in the ligands **L**_**allyl**_ and **1a,b** is downfield-shifted upon coordination, going from 5.76 to 6.00–6.64 ppm. Similar observations may be noted for the methyl resonances in the 3,5-dimethylpyrazolyl moieties, going from seemingly overlapped at 2.19 ppm in compounds **L**_**allyl**_ and **1a,b** to two distinct and upfield-shifted at 2.41 and 2.50 ppm upon coordination. The decrease in the symmetry of ligands **L**_**allyl**_ and **1a,b** in the complexes is caused by the coordination strain imparted in those fragments in complexes **3a,b** and **4**. Complex **4** presents the corresponding ethyl resonances, with the methylenic one appearing at −0.07 ppm (^3^*J*_HH_ = 9.0 Hz). Complex **5** presents ^1^H NMR resonances for one coordinated THF molecule at 3.62 and 1.78 ppm and C*H*_2_ and C*H*_3_ ethyl resonances at 0.65 and 0.35 ppm and from 0.29 to −0.09 ppm. The ^11^B NMR resonance of complex **5** is a sharp singlet at −17.9 ppm, characteristic of a borate anion containing bis(alkyl) and 9-BBN substituents.^47^ The characterization of complexes **2**–**5** by FTIR spectroscopy (Figures S41–S45 in the SI) evidenced the presence of 9-BBN or BCy_2_ and propylenic moieties by an increase of the intensity of CH bond vibration modes in the 2800–3000 cm^–1^ range, going from the **L**_**allyl**_ ligand in complexes **2** and **4** to the **1a** or **1b** ligands in complexes **3a**, **3b** and **5**. The structural formulations of complexes **4** and **5** are made on the basis of their spectroscopic characteristics, which are in line with the previous report by Lang and co-workers of diethylzinc complexes bearing the unfunctionalized **L**_**H**_ ligand.^48^ Though an intramolecular stabilization in complex **5** cannot be completely ruled out, the high symmetry exhibited for the proton and carbon resonances corresponding to the propyl bridge between the bis(3,5-dimethylpyrazolyl)methyl and borate moiety (*cf.* asymmetry of the same moieties in the NMR spectra of the intramolecular Lewis pair in compound **1c**), the coordination of a THF molecule and the very sharp nature of the respective ^11^B NMR resonance make the formulation, presented in Scheme 2, the most probable one.

**Scheme 3:**
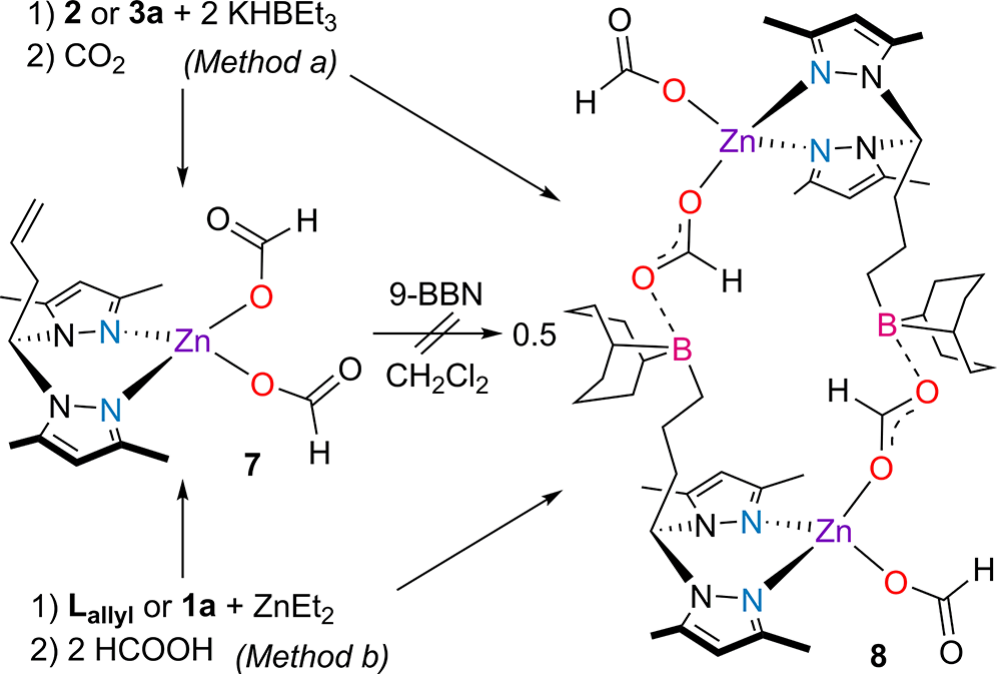
Synthesis of the Zinc Bis(Formate) Complexes **7** and **8**

Reactions of complexes **2** and **3a** with two equivalents of KHBEt_3_ or of **L**_**allyl**_ and **1a** with ZnH_2_ in THF systematically led to untraceable mixtures of complexes, regardless of the order in which the reagents were mixed or the control of the reactions temperatures. However, when the residues obtained from the reactions of complexes **2** or **3a** with two equivalents of KHBEt_3_ were placed under CO_2_ atmosphere, the heteroscorpionate zinc bis(formate) complexes **[(L**_**allyl**_**)Zn(OCHO)**_**2**_**] 7** or **[(1a)Zn(OCHO)**_**2**_**]**_**2**_**8** were respectively obtained, the latter a dimeric complex in which the 9-BBN moieties of one fragment intermolecularly interact with the terminal oxygen atoms of one of the formate ligands coordinated to the zinc center of the other fragment (Scheme 3, Method a).

By adapting the procedure reported by Lang and co-workers for the unfunctionalized **L**_**H**_ analogue,^48^ the reaction of **L**_**allyl**_ or **1a** with ZnEt_2_ in THF, followed by the addition of two equivalents of formic acid, also resulted in the formation of complexes **7** and **8**, in good yields (Scheme 3, Method b). Complexes **7** and **8** were characterized by NMR and FTIR spectroscopies and elemental analysis. The fact that the bis(formate) complexes **7** or **8** were, respectively, isolated from mixtures of complexes **2** or **3a** and 2 equivalents of KHBEt_3_ under CO_2_ atmosphere implies that zinc dihydride complexes, although not isolated, were present during those reactions.

The reaction of complex **7** with half equivalent of the 9-BBN dimer did not yield complex **8**, a complex of unknown formulation still containing the allyl functionality being observed instead. The addition of further equivalents of the 9-BBN dimer completely converted the allyl functionality, but a mixture of unidentified products, none of which corresponding to complex **8**, was isolated. Applying the same synthetic procedures to compound **1b** or complex **3b** did lead to the observation of formate functionalities, but the resulting reaction mixtures were significantly contaminated with other unknown compounds. Attempts to synthesize complexes **7** and **8** by reactions of complexes **2** and **3a**, respectively, with sodium formate were not successful. Performing reactions of ligands **1a**,**b** with ZnH_2_ under a CO_2_ atmosphere also led to untraceable mixtures.

**Figure 1:**
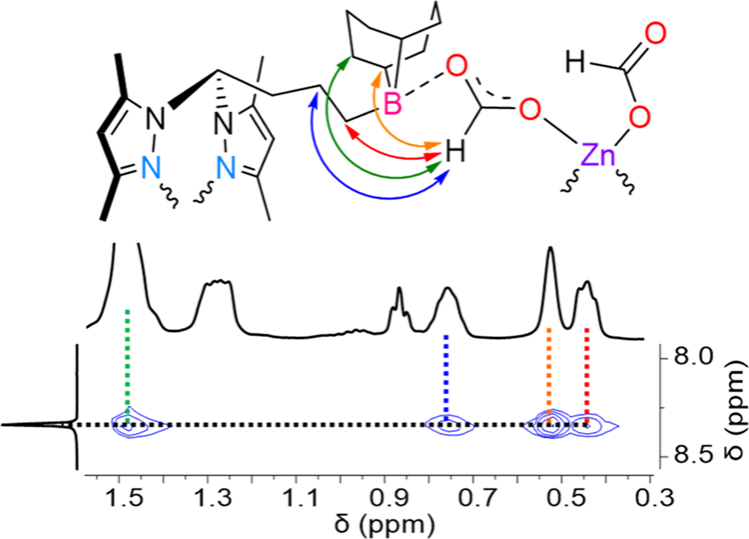
Section of the ^1^H–^1^H NOESY spectrum of complex **8** highlighting through-space NOE crosspeaks between the formate and 9-BBN/aliphatic chain proton resonances (bottom) along with a structure of complex **8** depicting said crosspeaks (top).

Complexes **7** and **8** were characterized by NMR spectroscopy (Figures S33–S37 in the SI). Aside from the resonances correspondent to symmetric **L**_**allyl**_ and **1a** moieties, the bis(formate) complexes **7** and **8** present ^1^H NMR resonances at 8.32–8.33 ppm, correlatable with the respective ^13^C NMR resonances at 167.0–170.1 ppm, respectively, assigned to the formate protons and carbons present in those complexes. The ^11^B NMR resonance in complex **8** is present at −21 ppm, characteristic of a tetracoordinate boron with a 9-BBN functionality. The ^1^H–^1^H NOESY spectrum of complex **8** (Figure S38 in the SI) displays a series of NOE off-diagonal crosspeaks between the formate resonance, at 8.33 ppm, and the 9-BBN CH_2_/9-BBN CH/propylic CH_2_ ones at 1.49–0.44 ppm (Figure 1).

Solutions of the bis(formate) complexes **7** and **8** were also analyzed by DOSY NMR spectroscopy (Figures S39 and S40), from which it was possible to observe that all resonances have a single diffusion coefficient (8.99 × 10^–7^ cm^2^ s^–1^ for complex **7** and 5.00 × 10^–7^ cm^2^ s^–1^ for complex **8**), meaning that, as expected, a single entity is present in each of the respective solutions. Moreover, the ratio of the hydrodynamic radii of complexes **7** and **8** is equal to 0.56. This means that complex **8** has approximately double the hydrodynamic radius of complex **7**.

The characterization of complexes **7** and **8** by FTIR spectroscopy (Figures S46 and S47 in the SI) evidenced the presence of 9-BBN or BCy_2_ and propylenic moieties by an increase of the intensity of CH bond vibration modes in the 2800–3000 cm^–1^ range, going from the **L**_**allyl**_ moiety in complex **7** to the **1a** moiety in complex **8**. The C=O stretching vibrations of the formate ligands in complexes **7** and **8** appear in the range of 1500–1600 cm^–1^.

The reasoning for the structural formulation of complex **8** is 4-fold: (1) the observation of ^1^H–^1^H NOE crosspeaks between the formate and 9-BBN/aliphatic chain proton resonances is only conceivable if those two sets of resonances are of different fragments; (2) the ^11^B NMR spectrum of complex **8** is characteristic of a tetracoordinate boron atom; (3) the high symmetry exhibited for the proton and carbon resonances corresponding to the propyl bridge between the bis(3,5-dimethylpyrazolyl)methyl and borane moieties discards any kind of intramolecular stabilization (*cf.* asymmetry of the same moieties in the NMR spectra of the intramolecular Lewis pair in compound **1c**); and (4) the DOSY NMR experiments performed on complexes **7** (a model monomeric complex) and **8**, evidenced the dimeric nature of the latter. Though the formation of complex **8** in a dimeric form was confirmed by NMR spectroscopy, elemental analysis, and FTIR spectroscopy, no single crystal was ever available, and, therefore, its existence as a monomeric entity cannot be completely ruled out.

The reaction of complex **8** with 6 equivalents of HBPin for 1 h (top of Figure S49 in the SI) led to the complete conversion of complex **8** to a new complex along with the formation of HCOOBPin, PinBOCH_2_OBPin and H_3_COBPin/PinBOBPin, in a 1:0.03:0.33 ratio. After 16 h, H_3_COBPin was the only observed product (bottom of Figure S49 in the SI). The new complex formed 1 h after the reaction between **8** and 6 equivalents of HBPin is presumably a zinc dihydride complex, in which the 9-BBN is stabilizing one of the hydride ligands, due to the appearance of a ^11^B NMR resonance at −23 ppm, diagnostic of a Zn–H–B interaction, no longer detectable after 16 h.

**Figure 2:**
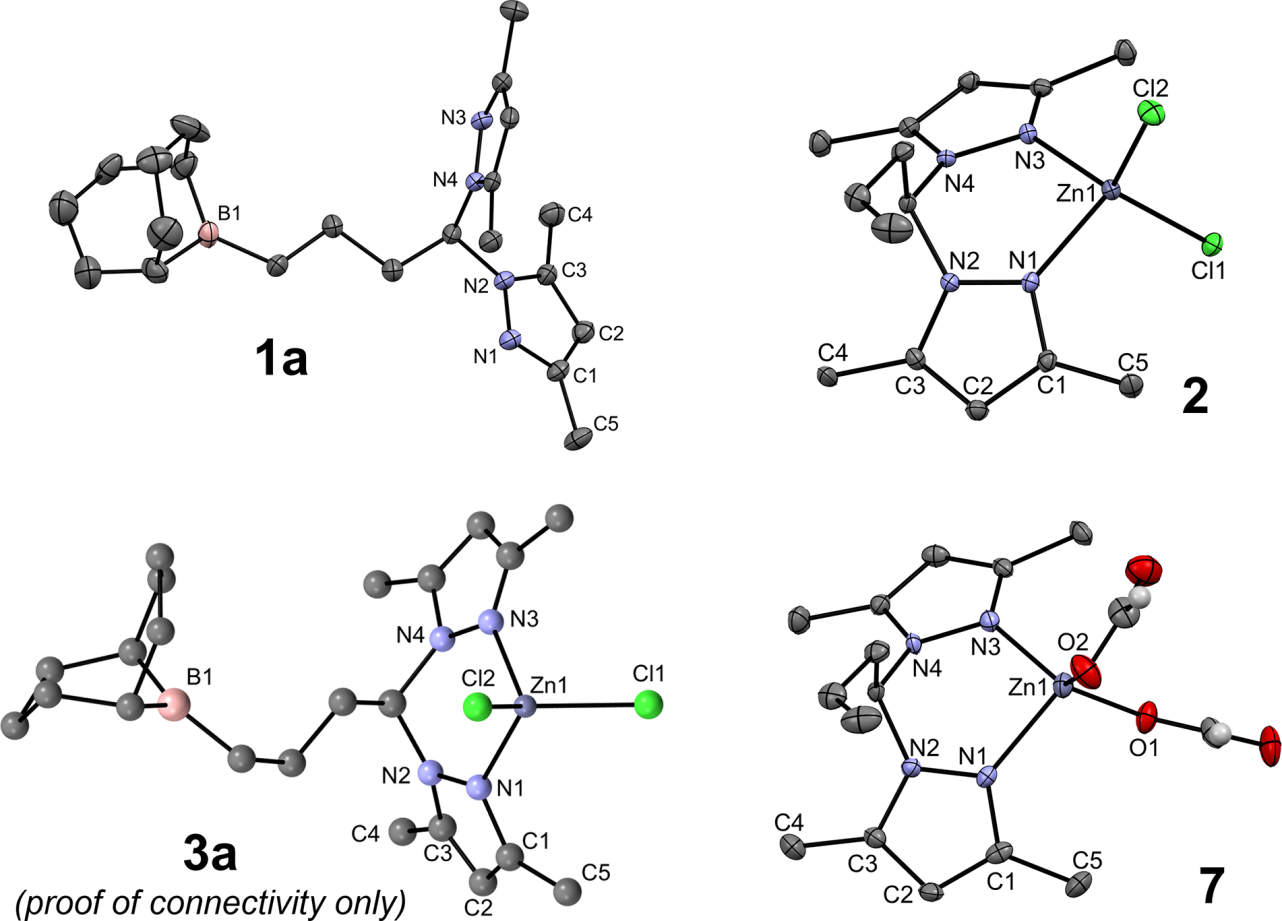
Single-crystal X-ray diffraction structures of compound **1a** and complexes **2** and **7**, showing 30% probability ellipsoids, and a proof of the molecular connectivity of complex **3a**. The hydrogen atoms (except those of the formate ligands in complex **7**) and the dichloromethane solvate molecule in complex **2** were omitted for the sake of clarity.

Compound **1a** and complexes **2**, **3a**, and **7** were analyzed by single-crystal X-ray diffraction. Compound **1a** crystallized in the triclinic system, in the *P*-1 space group, while complexes **2**/**7** and **3a** crystallized in the monoclinic system, in the *P*2_1_/*n* and *P*2_1_ space groups, respectively, with the structure of complex **2** being solved as a dichloromethane solvate. The molecular structures of compound **1a**, complexes **2** and **7**, and the proof of connectivity of complex **3a** (see the Experimental and Computational Methodologies in the SI) are presented in Figure 2, and selected bond lengths and angles and crystallographic data are displayed in Tables S1 and S2 in the SI.

The molecular structure of compound **1a** confirms the *anti*-Markovnikov nature of the addition of the 9-BBN dimer to the allyl function of compound **L**_**allyl**_, while also evidencing a staggered conformation of both 3,5-dimethylpyrazolyl rings and the linear propyl chain. The two fused 6-membered rings of the 9-BBN moiety in ligand **1a** adopt a chair–chair configuration.

Complexes **2** and **3a** present a κ^2^-*N*,*N* coordination mode of the **L**_**allyl**_ or **1a** ligands, respectively, aside from two chloride coligands. In complex **3a**, the borane arm was also evident in its molecular structure, with the three-carbon hydrocarbyl bridge between the bis(3,5-dimethylpyrazolyl)methane and 9-BBN moieties adopting a staggered conformation. The two fused 6-membered rings of the 9-BBN moiety in complex **3a** adopt a chair-boat configuration. The Zn1–N bond lengths are in the range 2.027(3)–2.06(2) Å, and the bite angle of the bis(3,5-dimethylpyrazolyl)methane chelate is in the range of 93.0(4)–93.04(12)°. The Zn1–Cl bond lengths in complexes **2** and **3a** are in the range of 2.192(5)–2.2336(10) Å. The coordination geometries of complexes **2** and **3a** are nearly tetrahedral, the τ_4_ parameters^49^ being in the range of 0.90–0.92. The chelation rings in complexes **2** and **3a** adopt boat configurations, whose α and β ring puckering parameters,^50^ respectively, defined as the angles between the {N1, N2, N3, N4} and {C11, N2, N4} and {N1, N2, N3, N4} and {Zn1, N1, N3} planes may be calculated. The α and β values for complexes **2** and **3a** are in the ranges of 49.05–52.57 and 5.22–9.43°, respectively. The structural features of complexes **2** and **3a** are in line with those crystallographically reported for zinc dichloride complexes with the bpm skeleton.^51^ The X-ray structure of complex **3a** could be accurately reproduced by density functional theory (DFT) calculations, as highlighted by the respective structure superimposition presented in Figure S48 in the SI.

Complex **7** presents a κ^2^-*N*,*N* coordination mode of the **L**_**allyl**_ ligand and two formate coligands displaying κ^1^-coordination modes. The Zn1–O bond lengths are in the range of 1.949(3)–1.967(3) Å. Furthermore, two orientations of the formate ligands within zinc complex **7** are observed: one has the uncoordinated oxygen atoms pointing away from the **L**_**allyl**_ ligand (distal configuration), while the other is pointing toward the methyl and methylene of the same scaffold (proximal configuration). Complex **7** has a distorted tetrahedral coordination geometry (τ_4_ = 0.82) and has α and β ring puckering parameters equal to 53.29 and 10.77°, respectively. Complex **7** is the first zinc bis(formate) complex with a bpm skeleton.

### DFT Studies on Relevant Zinc Dihydride and Bis(Formate) Complexes

DFT calculations^52^ were performed in order to gain insight on the possible zinc dihydride and bis(formate) structures containing ligands **1a,b**. Several possible structures were optimized: (1) monomeric structures containing tricoordinate “free” boranes, namely complexes **[(1a,b)ZnH**_**2**_**]**_**free**_ and **[(1a,b)Zn(OCHO)**_**2**_**]**_**free**_; (2) monomeric structures containing tetracoordinate “capped” boranes, through intramolecular interactions, namely complexes **[(1a,b)ZnH**_**2**_**]**_**capped**_ and **[(1a,b)Zn(OCHO)**_**2**_**]**_**capped**_; and (3) dimeric structures containing tetracoordinate “capped” boranes, through intermolecular interactions, namely complexes **[(1a,b)ZnH**_**2**_**]**_**2**_ and **[(1a,b)Zn(OCHO)**_**2**_**]**_**2**_. Several possible structures of dimeric zinc bis(formate) complexes containing ligand **1a** were explored, *e.g*., one in which both formate ligands of one center are “free” and those of the other are “capped” (complex **[(1a)Zn(OCHO**_**capped**_**)**_**2**_**][(1a)Zn(OCHO**_**free**_**)**_**2**_**]**) and those that contain mixed coordination modes of the formate ligands (complexes **[(1a)Zn(κ**^**1**^**/κ**^**2**^**OCHO)**_**2**_**]**_**2,A–D**_). The coordinates of all optimized structures are presented in the SI. A summary of selected structural parameters of the structures determined by DFT calculations is presented in Table 1.

**Table 1:** Selected Structural Parameters (Average Values) of Some of the Relevant Configurations of Zinc Dihydride (X = H) and Bis(Formate) (X = OC*H*O) Complexes Containing Ligands **1a,b**, Determined by DFT Calculations

complex	Zn–X (Å)	Zn–X(B) (Å)	B–X (Å)	τ_4_
**[(1a,b)ZnH**_**2**_**]**_**free**_	1.616	-	-	0.82
**[(1a,b)ZnH**_**2**_**]**_**capped**_	1.588	1.771	1.329	0.82
**[(1a)ZnH**_**2**_**]**_**2**_	1.601	1.756	1.349	0.87
**[(1b)ZnH**_**2**_**]**_**2**_	1.597	1.812	1.348	0.89
**[(1a,b)Zn(OCHO)**_**2**_**]**_**free**_	1.955	-	-	0.88
**[(1a,b)Zn(OCHO)**_**2**_**]**_**capped**_	1.938	2.033	1.609	0.78
**[(1a)Zn(OCHO)**_**2**_**]**_**2**_	1.936	2.000	1.617	0.85
**[(1b)Zn(OCHO)**_**2**_**]**_**2**_	1.939	2.004	1.612	0.84

The structures containing tricoordinate “free” boranes are structurally similar to complexes **3a**,**b** and **7** and present average Zn–H and Zn–O bond lengths equal to 1.616 and 1.955 Å, respectively. The structures containing tetracoordinate “capped” boranes possess, on average, terminal Zn–H and Zn–O bond lengths in the ranges 1.588–1.601 and 1.936–1.939 Å, respectively. The “capped” Zn–H and Zn–O bonds, containing Zn–H–B and Zn–O–B interactions, are on average 0.18 and 0.07 Å longer than terminal ones, respectively. This observation makes clear that the borane functionality is responsible for the weakening of the “capped” Zn–H/Zn–O bonds. Furthermore, the two C–O bond lengths of the formate ligands present in Zn–O–B interactions are similar, being on average 1.259 Å, evidencing electronic delocalization of the “capped” formate ligands, with two bridging O atoms: C–O–X (X = B or Zn). This contrasts with the electronic distinction between the two oxygen atoms of the terminal formate ligands that present a shorter C–O bond for the dangling O atom (*ca.* 1.23 Å). In all optimized structures containing ligand **1a**, the two fused 6-membered rings of the 9-BBN moiety adopt chair-boat configurations. In general, all zinc centers in the optimized structures display distorted tetrahedral coordination geometries, with τ_4_ parameters in the range of 0.79–0.89, except for complex **[(1a)Zn(OCHO)**_**2**_**]**_**capped**_, which presented an especially distorted coordination geometry (τ_4_ = 0.69).

**Figure 3:**
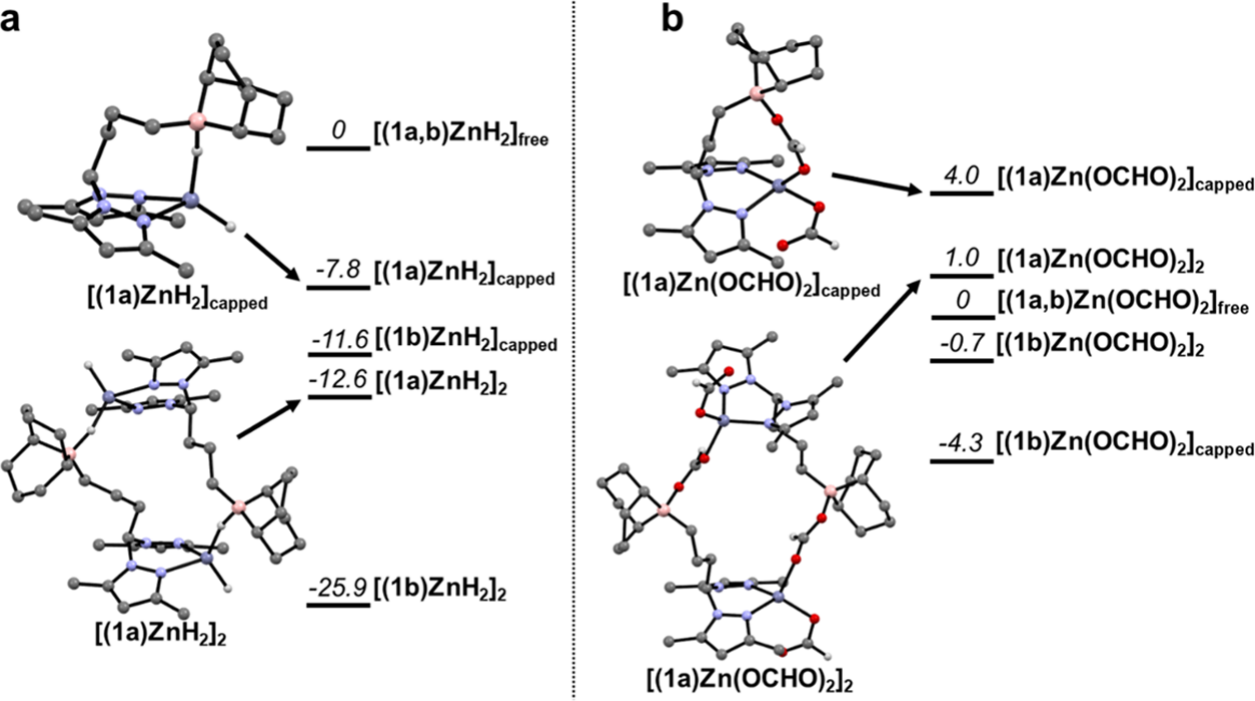
Selected structures and Gibbs energies (kcal mol^–1^, numbers in italics) of the relevant configurations of zinc dihydride (a) and bis(formate) (b) complexes containing ligands **1a,b**, determined by DFT calculations. All hydrogens have been omitted for clarity except those of the hydride and formate ligands. The black arrows are a visual guide.

The relative stability of the complexes was addressed by the calculations, and a selection of structures and the corresponding Gibbs energies is presented in Figure 3.

All of the zinc dihydride structures are stabilized by inter- or intramolecular interactions between the hydride ligands and neighboring boranes. Complexes **[(1a,b)ZnH**_**2**_**]**_**capped**_, bearing “capped” Zn–H motifs, sit 7.8 and 11.6 kcal mol^–1^ below the complexes **[(1a,b)ZnH**_**2**_**]**_**free**_, bearing “free” Zn–H motifs. These hydride species present further thermodynamic preference for complexes **[(1a,b)ZnH**_**2**_**]**_**2**_, with dimeric structures containing tetracoordinate “capped” boranes. Complexes **[(1a,b)ZnH**_**2**_**]**_**2**_ are 12.6 and 25.9 kcal mol^–1^ more stable than those bearing “free” Zn–H bonds. The structures with BCy_2_ substituents on the borane functionality, with ligand **1b**, show a greater thermodynamic preference for the formation of “capped” Zn–H fragments than those with 9-BBN ones, with ligand **1a**. This is particularly evident by a 13.3 kcal mol^–1^ energetic gain by replacing 9-BBN substituents for BCy_2_ ones in the dimeric complexes **[(1a,b)ZnH**_**2**_**]**_**2**_. The intramolecular stabilization of a zinc dihydride complex by a pending borane and consequent establishment of a Zn–H–B interaction has been previously reported in the literature.^31b^

When looking at the Gibbs energies of the zinc bis(formate) complexes, all structures containing ligand **1b** (with the BCy_2_ fragment) are stabilized by inter- or intramolecular interactions between boron and the formate ligand O atom: “capped” complexes **[(1b)Zn(OCHO)**_**2**_**]**_**c**_ and **[(1b)Zn(OCHO)**_**2**_**]**_**2**_, respectively, sit 4.3 and 0.7 kcal mol^–1^ below the “free” complex **[(1b)Zn(OCHO)**_**2**_**]**_**free**_. On the other hand, complex **[(1a)Zn(OCHO)**_**2**_**]**_**2**_ (that is, complex **8**) is only marginally less stable than complex **[(1a)Zn(OCHO)**_**2**_**]**_**free**_ (by 1.0 kcal mol^–1^), while **[(1a)Zn(OCHO)**_**2**_**]**_**capped**_ is 4.0 kcal mol^–1^ more energetic than complex **[(1a)Zn(OCHO)**_**2**_**]**_**free**_, further reinforcing the preferred experimental isolation of the dimeric complex **8**. All other structures considered resulted in more energetic species, being at least 5.4 kcal mol^–1^ less stable than complex **[(1a)Zn(OCHO)**_**2**_**]**_**free**_

While the isolation of the dimeric bis(formate) complex **8** validates the results obtained from the DFT calculations, the lack of success in the isolation of putative dihydride complexes lacks a similar validation. Given the low hydricity^53^ of borohydrides,^54^ the initial reaction of complexes **3a**,**b** with KHBEt_3_ in THF may also first involve the hydride transfer of the latter to the borane moieties of the former. In this reaction, the formation of zwitterionic complexes similar to complex **5**, in which the ethyl ligands are replaced by hydride ligands, cannot be ruled out.

The combined experimental and theoretical observations favor the stabilization of the hydride and formate coligands by the borane functionalities within ligands **1a,b** present in the complexes, thus envisioning importance in mediating CO_2_ hydroboration reactions.

### Catalytic Hydroboration of CO_2_ with HBPin

The zinc complexes **3a** and **3b** were tested as catalysts in the CO_2_ hydroboration with HBPin, and the results are summarized in Table 2.

**Table 2:**
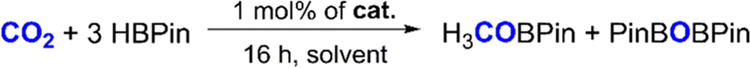
Hydroboration of CO_2_ with HBPin Catalyzed by the Zinc Complexes^a^

entry	**cat.**	solvent	temperature (°C)	yield (%)^b^
1	**3a**/2 KHBEt_3_	THF-*d*_8_	60	42
2	**3b**/2 KHBEt_3_	THF-*d*_8_	60	**86**
3	**2**/2 KHBEt_3_	THF-*d*_8_	60	17
4	**6**/2 KHBEt_3_	THF-*d*_8_	60	17
5	**6**/2 KHBEt_3_/*n*Oct(9-BBN)	THF-*d*_8_	60	20
6	**6**/2 KHBEt_3_/*n*OctBCy_2_	THF-*d*_8_	60	81
7	**3b**/2 KHBEt_3_	THF-*d*_8_	40	48
8	**6**/2 KHBEt_3_/*n*OctBCy_2_	THF-*d*_8_	40	18
9	**8**	THF-*d*_8_	60	43
10	**8^c^**	THF-*d*_8_	60	39
11	**3a**/2 KHBEt_3_	benzene-*d*_6_	60	16
12	**3a**	THF-*d*_8_	60	18
13	KHBEt_3_	THF-*d*_8_	60	8
14	-	THF-*d*_8_	60	0

a Conditions: 1 mol % **cat**.; 1 mmol of HBPin; and 1 bar of CO_2_. The yields were determined by ^1^H NMR spectroscopy utilizing 1,3,5-trimethoxybenzene as an internal standard.

b Based on HBPin.

c *In situ* prepared by reacting complex **3a** with two equivalents of KHBEt_3_ in THF, exposure to CO_2_ atmosphere, and evaporating all volatile materials.

Exposing THF-*d*_8_ solutions of HBPin to 1 bar of CO_2_ in the presence of 1 mol % of the catalyst system composed of complexes **3a** or **3b** and two equivalents of KHBEt_3_ at 60 °C for 16 h led to the selective formation of H_3_COBPin along with PinBOBPin in yields of 42 or 86%, respectively (Table 2, entries 1 and 2). The formation of H_3_COBPin/PinBOBPin was detected by ^1^H, ^13^C, ^11^B, and ^1^H–^13^C HSQC NMR spectroscopies (Figures S50–S53 in the SI), matching the data found in the literature (Table S3 in the SI).^14^ The reported yields correspond to the mixture H_3_COBPin/PinBOBPin and were determined by integration of the H_3_COBPin methoxy ^1^H NMR resonance against the 1,3,5-trimethoxybenzene standard and presented with respect to HBPin. In all productive catalytic reactions using KHBEt_3_ as the activator, the formation of BEt_3_ was detected by the presence of a minor ^11^B NMR resonance at 75 ppm.^55^ The improved yields when using precatalyst **3b** are due to the fact that putative dihydride/bis(formate) intermediates bearing the BCy_2_ borane are more prone to intra- or intermolecular stabilization, as indicated by DFT calculations.

In order to confirm the utility of the borane functionalities, zinc dichloride complexes **2** and **6**, respectively bearing borane-free **L**_**allyl**_ and **L**_**H**_ ligands, were also tested in the hydroboration of CO_2_ under the same conditions utilized for complexes **3a,b**. When the unfunctionalized catalyst systems **2**/2 KHBEt_3_ (Table 2, entry 3) and **6**/2 KHBEt_3_ (Table 2, entry 4), the hydroboration of CO_2_ also resulted in the formation of H_3_COBPin/PinBOBPin, but in yields of 17%. Additionally, catalytic experiments utilizing 1 mol % of the unfunctionalized catalyst systems **6**/2 KHBEt_3_ in the presence of 1 mol % of *n*Oct(9-BBN) (Table 2, entry 5) or *n*OctBCy_2_ (Table 2, entry 6), respectively, yielded H_3_COBPin/PinBOBPin in 20 or 81% yield. The catalytic hydroboration of CO_2_ at 40 °C using 1 mol % of **3b**/2 KHBEt_3_ (Table 2, entry 7) or **6**/2 KHBEt_3_/*n*OctBCy_2_ (Table 2, entry 8) proceeded with yields of 48 and 18%, respectively. These catalytic experiments show that the borane-tethered precatalysts **3a** and **3b** led to 2.5- to 6-fold improved catalytic activities in comparison with unfunctionalized catalyst systems in which the respective boranes were separately introduced. These observations indicate that the included borane functionalization contributed to an increase in the catalytic activity and confirmed the bifunctional characteristic of the present catalyst system. In addition, it was also verified that the catalytic activity benefits from intramolecular borane functionalization.

The single-component catalytic reaction utilizing 1 mol % of complex **8** (Table 2, entry 9) led to a yield of H_3_COBPin/PinBOBPin equal to 43%. When complex **8** was *in situ* prepared by reacting complex **3a** with two equivalents of KHBEt_3_ in THF, exposure to CO_2_ atmosphere, and evaporating all volatile materials and used as a catalyst under the same conditions, a 39% yield of H_3_COBPin/PinBOBPin was obtained (Table 2, entry 10). The yields obtained with isolated or *in situ* prepared complex **8** led to comparable yields with that obtained using the **3a**/KHBEt_3_ catalyst system. These results show that complex **8** is a catalytically active species and that BEt_3_, formed in the reactions using KHBEt_3_, has no influence on the catalytic results.

When performing the CO_2_ hydroboration with 1 mol % of **3a**/2 KHBEt_3_ in benzene-*d*_6_, the yield drops to 16% (Table 2, entry 11), indicative of the inability of benzene-*d*_6_ to solubilize both the precatalysts and the catalytic reaction intermediates. The reaction of **3a** (Table 2, entry 12) or KHBEt_3_ (Table 2, entry 13) on their own was met with 18 and 8% yield, respectively. Performing similar reactions at room temperature or in the absence of both **3a**/KHBEt_3_ (Table 2, entry 14) did not lead to any recordable yield.

**Figure 4:**
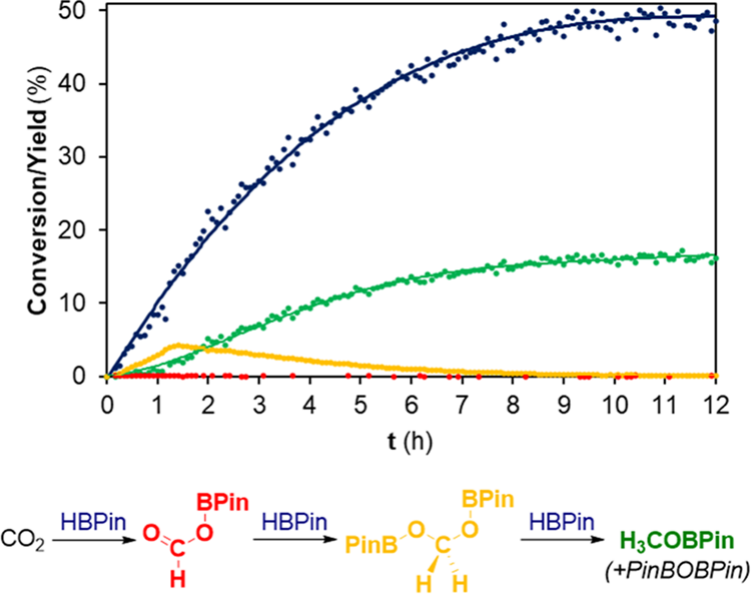
Kinetics of the hydroboration of CO_2_ with HBPin catalyzed by **3b**/2 KHBEt_3_, at 40 °C: evolution of the conversion of HBPin (blue data) and of the yields of the boronate esters resulting from the mono (red data), double (yellow data), and triple (green data) reduction of CO_2_. The colored lines are a visual guide. Conditions: 1 mol % of **3b**/2 KHBEt_3_; 1 mmol of HBPin; and 1 bar of CO_2_; solvent: THF-*d*_8_ (0.5 mL). The conversions and yields were determined by ^1^H NMR spectroscopy using 1,3,5-trimethoxybenzene as an internal standard.

The kinetics of CO_2_ hydroboration catalyzed by 1 mol % of **3b**/2 KHBEt_3_ at 40 °C was monitored and is presented in Figure 4.

For early reaction times (less than 15 min) and low HBPin conversions (less than 2%), the products deriving from the mono, double, and triple reduction of CO_2_, that is, products HCOOBPin, PinBOCH_2_OBPin and H_3_COBPin/PinBOBPin, respectively, were all detected. The assignment of the HCOOBPin and PinBOCH_2_OBPin products was made by comparing the respective formate and methylenic proton resonances (Table S3 in the SI) with those reported in the literature.^ ^14,18c^^ The HCOOBPin product was completely converted early on, while the amount of PinBOCH_2_OBPin increased until HBPin conversion reached 12%. From this point on, PinBOCH_2_OBPin was gradually converted onto H_3_COBPin/PinBOBPin, with the latter being the only observed product at the end of the reaction. The first (10 min) and last (16 h) ^1^H NMR spectra acquired during the kinetic monitoring have been presented in Figure S54, while the resonances used for the calculations are presented in Table S3 in the SI.

The present catalyst system served as a good platform for the selective transformation of CO_2_ to the methanol-level product H_3_COBPin, achieving a maximum Turnover Frequency of 5.4 h^–1^, faring well with the results found in the literature for main group elements and first-row transition metals under dilute conditions and using 1 bar of CO_2_ pressure, which were in the range of 0.1–3.3 h^–1^. For a more detailed comparison, a summary of the different results obtained with previously reported catalysts for the hydroboration of CO_2_ with HBPin displaying methoxy selectivity has been presented in Table S4 in the SI.

**Scheme 4:**
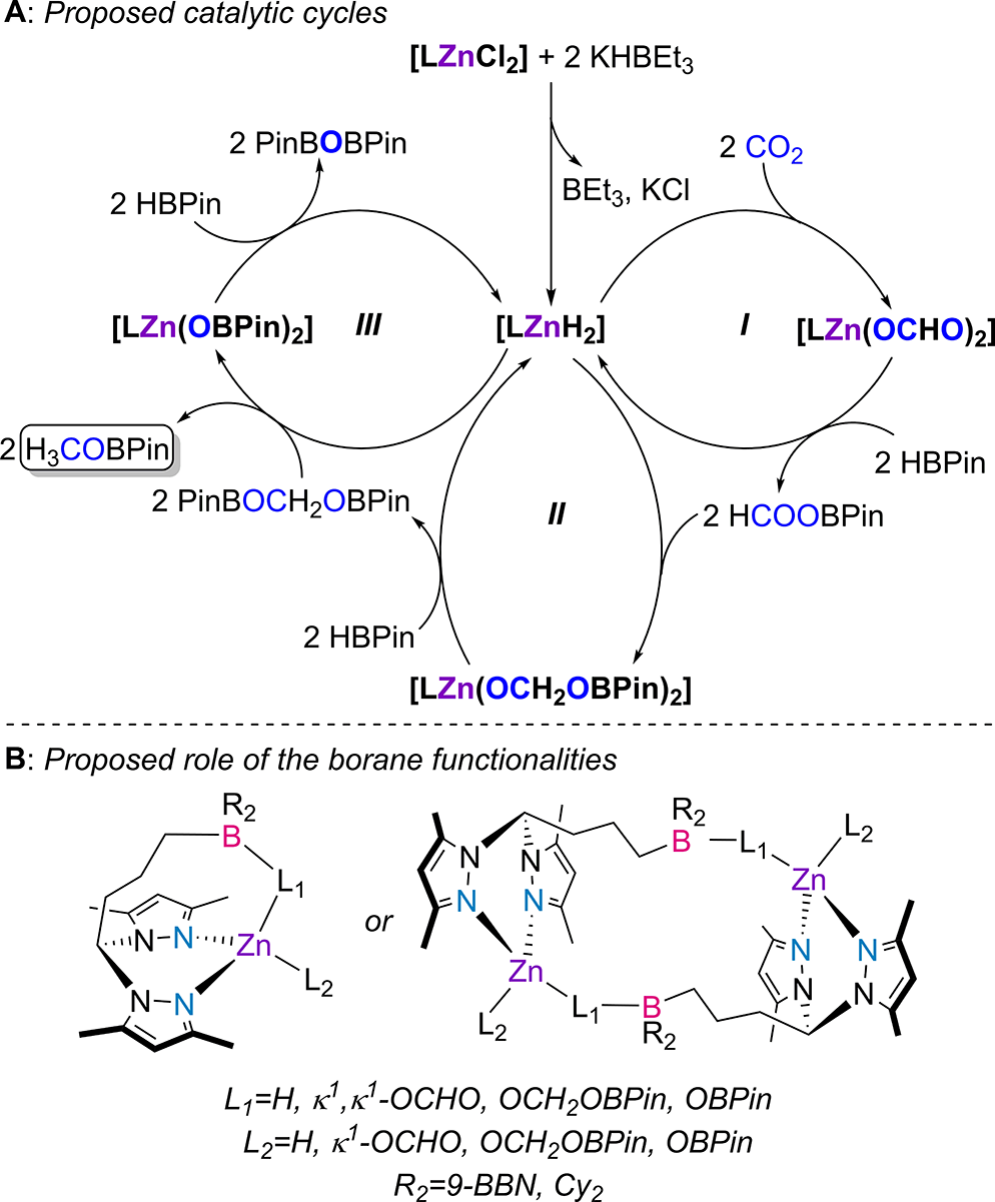
Mechanistic Proposal for the Hydroboration of CO_2_ with HBPin Catalyzed by Complexes **3a,b**/2 KHBEt_3_, Where L Denotes Ligands **1a,b** (**A**), and Proposed Role of the Borane Funcrionalities (**B**)

Based on experimental evidence, a mechanism for the hydroboration of CO_2_ with HBPin catalyzed by **3a**,**b**/2 KHBEt_3_ is proposed (Scheme 4, A).

The activation of complexes **3a**,**b** by KHBEt_3_ likely originates dihydride complexes of the type **[LZnH**_**2**_**]**, with the observable formation of BEt_3_ (by ^11^B NMR spectroscopy) and KCl. Exposure of putative **[LZnH**_**2**_**]** complexes to a CO_2_ atmosphere promptly gives rise to the bis(formate) complexes **[LZn(OCHO)**_**2**_**]**, *via* insertion reactions. This reactivity has been experimentally verified by the isolation of bis(formate) complex **8**, containing ligand **1a**. Complexes **[LZn(OCHO)**_**2**_**]** react with HBPin in σ–bond metathesis reactions giving rise to HCOOBPin along with the regeneration of **[LZnH**_**2**_**]** intermediates (Scheme 4, Cycle **I**).

The HCOOBPin product may again insert onto the Zn–H bonds of **[LZnH**_**2**_**]** intermediates, yielding complexes of the type **[LZn(OCH**_**2**_**OBPin)**_**2**_**]**. Complexes **[LZn(OCH**_**2**_**OBPin)**_**2**_**]** again react with HBPin, regenerating **[LZnH**_**2**_**]** and forming PinOCH_2_OBPin (Scheme 4, Cycle **II**). Although the formation of intermediate **[LZn(OCH**_**2**_**OBPin)**_**2**_**]** has not been experimentally observed, HCOOBPin and PinOCH_2_OBPin have both been detected in the reaction of complex **8** with 3 equivalents of HBPin and in the kinetic monitoring of a catalytic run.

Further reaction of intermediates **[LZn(OCH**_**2**_**OBPin)**_**2**_**]** with HBPin presumably leads to the formation of H_3_COBPin along with intermediates **[LZn(OBPin)**_**2**_**]**. Intermediates **[LZn(OBPin)**_**2**_**]** may again react with HBPin regenerating the hydride complexes **[LZnH**_**2**_**]** and forming PinBOBPin, with the formation of the latter also experimentally observed (Scheme 4, Cycle **III**). Mechanistic studies on metal-catalyzed CO_2_ hydroboration have indicated that β-boroxide elimination in [M(OCH_2_OBPin)] intermediates led to the formation of formaldehyde and intermediates [M(OBPin)].^18c^ However, although the formation of formaldehyde cannot be ruled out, we did not detect its formation. Because of this, the mechanism proposed by Trovitch, avoiding formaldehyde, seemed the most plausible one.^17^

The initial formation of complexes **[LZnH_2_]** and **[LZn(OCHO)_2_]** having equal coligands is governed by the initial stoichiometry of complexes **[LZnCl_2_]** and KHBEt_3_. However, the formation of the subsequent methanolate and borolanolate intermediates and regeneration of the hydride/formate intermediates may lead to complexes bearing mixed coligands. Such mixed coligand complexes containing hydride, formate, methanolate, and borolanolate moieties are also expected to exhibit catalytic turnover and may not be ruled out.

The appended boranes in precatalysts **3a**,**b** (as opposed to their absence in complexes **2** and **6**) play a role in kinetically stabilizing all catalytic cycles intermediates, by means of establishing Lewis pairs between hydrogen/oxygen-based ligands and the respective borane functions, that is, the establishment of Zn–H–B or Zn–O–B interactions (Scheme 4, B). Such Zn–H–B or Zn–O–B interactions may, in one aspect, facilitate the insertion and σ-bond metathesis reactions occurring during catalysis by weakening the respective Zn–H/Zn–O bonds. The conversion of **[LZn(OCH**_**2**_**OBPin)**_**2**_**]** intermediates with HBPin into **[LZn(OBPin)**_**2**_**]** ones may particularly benefit from intra- or intermolecular borane stabilization, due to the highly polarized nature of the terminal C–O bond, where, potentially, the oxygen atom is interacting with both BPin and 9-BBN/BCy_2_ fragments. On the other hand, Zn–H–B or Zn–O–B may also contribute to avoid the formation of aggregates that would otherwise form when dealing with unprotected Zn–H/Zn–O species. The prevention of such aggregates increases the solubility of the respective reaction intermediates, increasing their catalytic activity.

## Conclusions

In this work, new borane-tethered heteroscorpionate bis(3,5-dimethylpyrazolyl)methane ligands amplified the zinc-catalyzed hydroboration of CO_2_ to methanol-level products.

The newly prepared allylated bis(3,5-dimethylpyrazolyl)methane analogue **L**_**allyl**_ served as a precursor to three new borane-tethered ligands **1a**–**c**, *viaanti*-Markovnikov hydroboration reactions. Metalation of **1a,b** with ZnCl_2_ or ZnEt_2_ yielded dichloride complexes **3a,b** or zwitterionic complex **5**. The reaction of complex **3a** with two equivalents of KHBEt_3_ in the presence of CO_2_ gave rise to the formation of the bis(formate) complex **8**, in which the 9-BBN moieties of one fragment intermolecularly interact with the terminal oxygen atoms of one of the formate ligands coordinated to the zinc center of the other fragment. The complexes containing the **L**_**allyl**_ ligand were also synthesized (complexes **2**, **4** and **7**). Similar coordination chemistry using compound **1c** proved unsuccessful. All complexes were structurally characterized by NMR spectroscopy, FTIR spectroscopy, and elemental analysis and selected cases by single-crystal X-ray diffraction. The nuclearity of complex **8** was verified by DOSY NMR spectroscopy. Several possible dihydride and bis(formate) complexes bearing ligands **1a**,**b** were studied by DFT calculations, with the structures bearing the BCy_2_ function being more prone to intra- or intermolecular stabilization by the borane than those with the 9-BBN one.

The catalyst systems composed of 1 mol % of **3a** or **3b** with two equivalents of KHBEt_3_ successfully promoted the hydroboration of CO_2_ at 1 bar with HBPin to the methanol-level product H_3_COBPin (along with PinBOBPin) in 42 or 86% yields, respectively. Catalysis using complex **8** as a single-component catalyst system also led to yields (39–43%) comparable to those obtained with **3a**/2 KHBEt_3_. The catalyst system composed of the borane-free complex **6** with two equivalents of KHBEt_3_ or by **6**/2 KHBEt_3_/*n*OctBR_2_ (BR_2_ being 9-BBN or BCy_2_) also hydroborated CO_2_ to H_3_COBPin but in 2.5- to 6-fold lower activities than those exhibited when using the catalyst systems **3a,b**/2 KHBEt_3_ or **8**, confirming the advantage of including intramolecular borane functionalities.

The boranes appended in the precatalysts kinetically stabilize the catalytic intermediates by establishing Zn–H–B or Zn–O–B interactions. Such interactions may facilitate the insertion/σ-bond metathesis reactions occurring during catalysis, while also preventing the formation of aggregates and increasing the solubility and catalytic activity of the reaction intermediates. The present work has reinforced the potential and implications of secondary coordination sphere tuning in in the catalytic conversion of CO_2_.

## Supporting Information Available

Experimental and computational methodologies; synthetic and catalytic experimental procedures; NMR spectra of the compounds; DOSY NMR data for complexes **7** and **8;** FTIR spectra of the complexes; supplementary single-crystal X-ray diffraction data; NMR data of the reaction of complex **8** with HBPin; selected NMR data of the catalytic reactions; literature comparison of catalytic results and references (PDF)
All calculated species coordinates joint (XYZ)

## Supplementary Material

ic4c00500_si_001.pdf

ic4c00500_si_002.xyz
