# Supplementary material for: Zinc-Catalyzed Hydroboration of Carbon Dioxide Amplified by Borane-Tethered Heteroscorpionate Bis(Pyrazolyl)methane Ligands
Source: Inorg Chem. 2024 Apr 24;63(18):8244–56. doi: 10.1021/acs.inorgchem.4c00500 (PMC11080050; doi:10.1021/acs.inorgchem.4c00500)
Supplement: Supplementary file 1 — ic4c00500_si_001.pdf [file ic4c00500_si_001.pdf]

Supporting Information for

Zinc-catalyzed Hydroboration of Carbon Dioxide Amplified by Borane-tethered Heteroscorpionate *bis*(Pyrazolyl)methane Ligands

Tiago F. C. Cruz,[1]* Valentin Loupy,[1] Luís F. Veiros,[1]

[1] *Centro de Química Estrutural, Institute of Molecular Sciences, Departamento de Engenharia Química, Instituto Superior Técnico, Universidade de Lisboa, Av. Rovisco Pais, 1049 001 Lisboa, Portugal.*

*Corresponding Author; e-mail: carpinteirocruz@tecnico.ulisboa.pt

Table of contents

Table of contents....S1
Experimental and computational methodologies....S2
Synthetic and catalytic experimental procedures....S5
NMR spectra of the compounds....S18
DOSY NMR data for complexes **7** and **8**....S40
FTIR spectra of the complexes....S41
Supplementary single crystal X-ray diffraction data....S45
NMR data of the reaction of complex **8** with HBPin....S48
Selected NMR data of the catalytic reactions....S49
Literature comparison of catalytic results....S52
References....S53

Experimental and computational methodologies

General procedures: Unless otherwise stated, all operations were performed under dry dinitrogen (N_2) atmosphere using standard glovebox and Schlenk techniques. N_2 and carbon dioxide (CO_2) gases used for all operations (purity: <1 ppm O_2 and H_2O) were supplied by Air Liquide and further purified by passage through 4 Å molecular sieves and SICAPENT®, respectively. Solvents and solutions were transferred using a positive pressure of N_2 through stainless steel cannulae and mixtures were filtered in a similar way using modified cannulae that could be fitted with glass fiber filter disks. Solvents were pre-dried with activated 4 Å molecular sieves and distilled by heating under N_2 over suitable drying agents (sodium/benzophenone for toluene and THF; CaH_2 for *n*-hexane and dichloromethane) and stored under N_2 in J. Young-type ampoules. All deuterated solvents used were dried over activated 4 Å molecular sieves, degassed by the freeze-pump-thaw technique and stored under N_2 in J. Young-type ampoules. $ZnCl_2$ was heated under dynamic vacuum at 100 ºC until constant weight prior to use. Formic acid and HBPin were distilled trap-to-trap prior to use. *bis*(3,5-Dimethylpyrazolyl)methane (**L_H**),[1] complex **[(L_H)$ZnCl_2$]** (**6**),[2] $HBCy_2$,[3] $HB(C_6F_5)_2$,[4] 9-(*n*-octyl)-9-borabicyclo[3.3.1]nonane (*n*Oct(9-BBN)),[5] and dicyclohexyl(*n*-octyl)borane (*n*OctBCy$_2$)[6] were prepared as described in the literature. The remaining reagents were used as received from commercial sources (*e.g.* Acros, Alfa Aesar, Merck). Elemental analyses were obtained from the elemental analysis service of Instituto Superior Técnico, Universidade de Lisboa, on a Fisons Instrument Mod EA-1108. FTIR measurements were performed on a Bruker Alpha II ATR IR spectrometer located inside a glovebox.

NMR spectroscopy measurements: NMR spectra were recorded on a *Bruker "AVANCE III"* 300 MHz spectrometer at 299.995 MHz (^{1}H), 75.4296 MHz (^{13}C), 96.2712 MHz (^{11}B), and 282.404 (^{19}F). The spectra were referenced internally using the residual protio-resonances (^{1}H) and the solvent carbon (^{13}C) resonances of the corresponding solvents[7] to tetramethylsilane ($\delta = 0$), and referenced externally using 15% $BF_3 \cdot OEt_2$ ($\delta = 0$), for ^{11}B, and $CFCl_3$ ($\delta = 0$) for ^{19}F. All solution samples, excluding those involving organic molecules devoid of boranes, were prepared inside a glovebox and transferred to screw-capped or J. Young NMR tubes. All chemical shifts are quoted in δ (ppm) and coupling constants (J) in Hz with multiplicities abbreviated as br (broad), s (singlet), d (doublet), t (triplet), q (quartet), h (heptet) and m (multiplet). The DOSY NMR spectra were obtained at 25 ºC using a bipolar stimulated echo

sequence (STE) with smoothed square gradients. The average diffusion coefficients of complexes **7** and **8** were converted to the respective hydrodynamic radii by means of the Stokes-Einstein equation.[8]

X-ray diffraction: Crystallographic and experimental details of crystal structure determinations are listed in Tables S1 and S2 of the ESI. The crystals were selected under an inert atmosphere, covered with polyfluoroether oil and mounted on a nylon loop. Crystallographic data were collected using graphite monochromated Mo-Kα radiation (λ = 0.71073 Å) on a Bruker AXS-KAPPA APEX II diffractometer equipped with an Oxford Cryosystem open-flow dinitrogen cryostat, at 150 K. Cell parameters were retrieved using Bruker SMART[9] software and refined using Bruker SAINT[10] on all observed reflections. Absorption corrections were applied using SADABS.[11] Structure solution and refinement were performed using direct methods with the programs SIR2014[12] and SHELXL[13] included in the package of programs WINGX-Version 2014.1.[14] Complex **3a** contained highly disordered solvate molecules in an intermediate electron density map and, therefore, the SQUEEZE routine, included in PLATON,[15] was included, since no appropriate disorder model could be applied. The CIF file corresponding to the molecular structure of complex **3a** presented B-level alerts, which were associated with a high R_{int} and low precision of selected bonds, owing to the poor diffracting power/lack of unique data of the crystal and the corresponding data atom after the completion of the structure refinement. Nevertheless, it was possible to solve the molecular structure, though only presented in the article as a proof of its molecular connectivity. All non-hydrogen atoms were refined anisotropically, and the hydrogen atoms were inserted in idealized positions and allowed to refine riding on the parent carbon atom. Graphic presentations were prepared with Mercury 2022.3.0.[16] Data was deposited in CCDC under the deposit numbers 2328683 for compound **1a**, 2328684 for complex **2**, 2328685 for complex **3a** and 2328686 for complex **7**.

Computational details: Calculations were performed using the GAUSSIAN 09 software package[17] and the PBE0 functional, without symmetry constraints. That functional uses a hybrid generalized gradient approximation (GGA), including 25 % mixture of Hartree-Fock[18] exchange with DFT[19] exchange-correlation, given by Perdew, Burke and Ernzerhof functional (PBE).[20] The optimized geometries were obtained with the Stuttgart Effective Core Potentials and associated basis set (SDD)[21] with an added f-polarization function[22] for Zn, and a standard 6-31G(d,p)[23] for the remaining elements (basis b1). The electronic energies (E_{b1}) obtained at

the PBE0/b1 level of theory were converted to free energy at 298.15 K and 1 atm (G_{b1}) by using zero point energy and thermal energy corrections based on structural and vibration frequency data calculated at the same level.

Single point energy calculations were performed on the geometries obtained at the PBE0/b1 level using the same functional and a 6-311++G(d,p) basis set.[24] Solvent effects (THF) were accounted for in all calculations (including geometry optimizations) by means of the Polarisable Continuum Model (PCM) initially devised by Tomasi and coworkers[25] with radii and non-electrostatic terms of the SMD solvation model, developed by Truhler *et al.*[26] The free energy values presented (G_{b2}-D3) were corrected for dispersion by means of Grimme DFT-D3 method[27] with Becke and Johnson short distance damping,[28] being derived from the electronic energy values obtained at the PBE0-D3/6-311++G(d,p)//PBE0/b1 level (E_{b2}-D3) according to the following expression: (G_{b2}-D3) = (E_{b2}-D3) + G_{b1} – E_{b1}.

Synthetic and catalytic experimental procedures

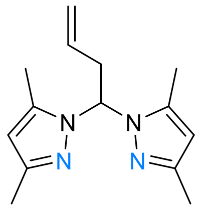

Synthesis of $\mathbf{L_{allyl}}$: ***Caution!*** *n-Butyllithium is extremely pyrophoric. It must be handled using proper inert atmosphere stainless steel cannula transfer techniques.* *n*-Butyllithium (12 mmol, 4.8 mL of a 2.5 M solution in *n*-hexane) was added to a THF solution of $\mathbf{L_H}$ (10 mmol, 2.04 g) cooled to -78 ºC, by means of an ethanol/liquid dinitrogen cold bath. The resulting tan suspension was stirred for 2 h without removing the cold bath. Allyl bromide (12 mmol, 1.0 mL) was added to the cold suspension and the mixture was stirred at room temperature overnight. All volatiles were removed under reduced pressure. Beyond this point, maintaining a N_2 atmosphere was no longer necessary. The mixture was treated with 100 mL of a diethyl ether/water mixture. The phases were separated, the aqueous phase was extracted with diethyl ether and the organic extracts combined and evaporated to dryness in the rotative evaporator. The crude was purified by flash column chromatography using a 5:1 ethyl acetate:*n*-hexane eluent, yielding a white microcrystalline powder. Yield: 1.12 g (46%). Anal. Calc. for $C_{14}H_{20}N_4$, obtained (calculated): C 68.95 (68.82), H 8.12 (8.25), N 22.53 (22.93). ^{1}H NMR (300 MHz, $CDCl_3$): δ 6.25 (1H, t, NC*H*N, $^3J_{HH}$ = 9.0 Hz), 5.77 (2H, s, C$H_{pyrazole}$), 5.62 (1H, m, =CH_{allyl}), 5.19-5.01 (2H, m, =CH_{2allyl}), 3.34 (2H, t, CH_{2allyl}, $^3J_{HH}$ = 9.0 Hz), 2.19 (12H, br s, C$H_{3pyrazole}$). ^{13}C{^{1}H} NMR (75 MHz, $CDCl_3$): δ 147.6 (*C*$Me_{pyrazole}$), 139.8 (*C*$Me_{pyrazole}$), 132.7 (=CH_{allyl}), 118.9 (=*C*H_{2allyl}), 106.9 (*C*$H_{pyrazole}$), 72.5 (N*C*HN), 37.5 (CH_{2allyl}), 13.7 (C$H_{3pyrazole}$), 11.3 (C$H_{3pyrazole}$).

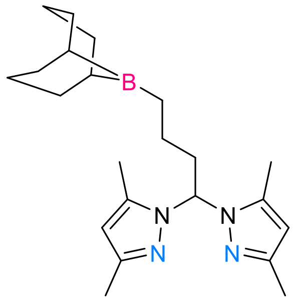

Synthesis of 1a: Toluene was added to a solid mixture of **L$_{allyl}$** (0.77 mmol, 0.19 g) and the 9-H-9-Borabicyclo[3.3.1]nonane (9-BBN) dimer (0.42 mmol, 0.10 g) and the resulting solution was stirred at 80 ºC for 1 h. All volatiles were removed under reduced pressure, the residue redissolved in *n*-hexane, the solution filtered and evaporated to dryness, from which a colorless oil was formed which crystallized upon standing. Crystals suitable for X-ray diffraction from a concentrated *n*-hexane solution cooled to -20 ºC. Yield: 0.25 g (88%). Anal. Calc. for $C_{22}H_{35}BN_4$•0.25(–OSi$(CH_3)_2$–), obtained (calculated): C 70.09 (70.21), H 10.01 (9.56), N 14.99 (14.56). ^{1}H NMR (300 MHz, $CDCl_3$): δ 6.29 (1H, t, NC*H*N, $^3J_{HH}$ = 9.0 Hz), 5.76 (2H, s, C*H*$_{pyrazole}$), 2.61 (2H, q, B-CH_2CH_2C*H*$_2$-C, $^3J_{HH}$ = 6.0 Hz), 2.19 (12H, s, C*H*$_{3pyrazole}$), 1.88-1.38 (16H, m, C*H*$_{9\text{-BBN}}$ + C*H*$_{2,9\text{-BBN}}$ + B-CH_2C*H*$_2CH_2$-C), 1.17 (2H, t, B-C*H*$_2CH_2CH_2$-C, $^3J_{HH}$ = 9.0 Hz). ^{13}C{^{1}H} NMR (75 MHz, $CDCl_3$): δ 147.2 (*C*Me$_{pyrazole}$), 139.9 (*C*Me$_{pyrazole}$), 106.9 (*C*H$_{pyrazole}$), 73.3 (N*C*HN), 35.8 (B-CH_2CH_2C*H*$_2$-C), 33.2 (*C*H$_{2,9\text{-BBN}}$), 31.0 (*C*H$_{9\text{-BBN}}$), 23.4 (B-C*H*$_2CH_2CH_2$-C), 23.3 (*C*H$_{2,9\text{-BBN}}$), 20.8 (B-CH_2C*H*$_2CH_2$-C), 13.7 (C*H*$_{3pyrazole}$), 11.3 (C*H*$_{3pyrazole}$).

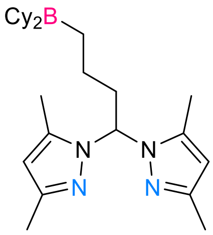

Synthesis of 1b: Toluene was added to a solid mixture of **L$_{allyl}$** (2.0 mmol, 0.66 g) and dicyclohexylborane (2.2 mmol, 0.39 g) and the resulting solution was stirred at 80 ºC for 1 h. All volatiles were removed under reduced pressure, the residue redissolved in n-hexane, the solution filtered and evaporated to dryness, from which a colorless oil was formed which crystallized upon standing. Yield: 0.70 g (83%). Anal. Calc. for $C_{26}H_{43}BN_4$•0.25(–OSi$(CH_3)_2$–), obtained (calculated): C 73.78 (73.92), H 10.15 (10.26), N 13.01 (13.26). ^{1}H NMR (300 MHz, $CDCl_3$): δ 6.29 (1H, t, NC*H*N, $^3J_{HH}$ = 7.5 Hz), 5.76 (2H, s, C*H*$_{pyrazole}$), 2.58 (2H, q, C*H*$_2$-NCHN, $^3J_{HH}$ = 8.0 Hz), 2.19 (12H, s, C*H*$_{3pyrazole}$), 1.73-1.05 (33H, m, B-CH_2CH_2C*H*$_2$-C + B-CH_2C*H*$_2$$CH_2$-C + B-C*H*$_2$$CH_2CH_2$-C + C*H*$_{BCy2}$ + C*H*$_{2BCy2}$). ^{13}C{^{1}H} NMR (75 MHz, $CDCl_3$): δ 147.3 (*C*Me$_{pyrazole}$), 139.9 (*C*Me$_{pyrazole}$), 106.9 (*C*H$_{pyrazole}$), 73.2 (N*C*HN), 36.2 (B*C*H), 27.7 (*C*$_{BCy2}$), 27.1 (*C*$_{BCy2}$), 20.3 (B*C*H$_2$), 13.7 (C*H*$_{3pyrazole}$), 11.3 (C*H*$_{3pyrazole}$). ^{11}B NMR (96 MHz, $CDCl_3$): δ 83.1 (br, *B*Cy$_2$).

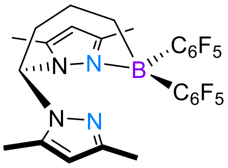

Synthesis of 1c: Toluene was added to a solid mixture of **L$_{allyl}$** (1.0 mmol, 0.24 g) and $HB(C_6F_5)_2$ (1.1 mmol, 0.38 g) and the resulting solution was stirred at 80 ºC for 1 hour. All volatiles were removed under reduced pressure at room temperature, the residue was extracted with *n*-hexane, the solution filtered and evaporated to dryness, from which a colorless foam was formed. The crude product was recrystallized as a microcrystalline white powder from a concentrated *n*-hexane solution cooled to -20 ºC. Yield: 0.38 g (65%). Anal. Calc. for $C_{26}H_{21}BF_{10}N_4$, obtained (calculated): C 53.12 (52.91), H 3.53 (3.59), N 9.34 (9.49). ^{1}H NMR (300 MHz, $CDCl_3$): δ 6.47 (1H, dd, NC*H*N, $^3J_{HH}$ = 12.0 Hz; $^2J_{HH}$ = 3.0 Hz), 5.96 (1H, s, C$H_{pyrazole}$), 5.87 (1H, s, C$H_{pyrazole}$), 2.90 (1H, m, B-$CH_2CH_2CH_2$-C), 2.34-2.18 (4H, m, B-$CH_2CH_2CH_2$-C + C$H_{3pyrazole}$), 1.96-1.82 (4H, m, B-$CH_2CH_2CH_2$-C + C$H_{3pyrazole}$), 1.78 (3H, s, C$H_{3pyrazole}$), 1.65-1.47 (4H, m, B-$CH_2CH_2CH_2$-C + C$H_{3pyrazole}$), 1.43-1.22 (2H, m, B-$CH_2CH_2CH_2$-C). ^{13}C{^{1}H} NMR (75 MHz, $CDCl_3$): δ 152.7 (*C*Me$_{pyrazole}$), 150.0 (C_6F_5), 148.5 (*C*Me$_{pyrazole}$), 146.6 (C_6F_5), 141.0 (*C*Me$_{pyrazole}$), 140.8 (C_6F_5), 139.0 (*C*Me$_{pyrazole}$), 137.8 (C_6F_5), 135.7 (C_6F_5), 111.0 (*C*H$_{pyrazole}$), 108.0 (*C*H$_{pyrazole}$), 74.6 (N*C*HN), 33.8 (CH_2), 21.1 (CH_2), 15.4 (C$H_{3pyrazole}$), 13.6 (C$H_{3pyrazole}$), 11.0 (C$H_{3pyrazole}$), 9.9 (C$H_{3pyrazole}$). ^{11}B NMR (96 MHz, $CDCl_3$): δ -4.0 (br, *B*($C_6F_5)_2$). ^{19}F NMR (282 MHz, $CDCl_3$): δ -129.7 (1F, br, *ortho*-B($C_6F_5)_2$), -132.6 (2F, br, *ortho*-B($C_6F_5)_2$), -158.1 to -158.4 (2F, m, *para*-B($C_6F_5)_2$), -163.4 (2F, m, *meta*-B($C_6F_5)_2$), -164.0 (1F, m, *meta*-B($C_6F_5)_2$), -164.6 (1F, m, *meta*-B($C_6F_5)_2$).

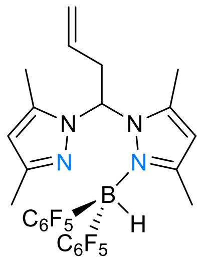

L_{allyl}•HB(C_6F_5)$_2$: Toluene was added to a solid mixture of **L_{allyl}** (0.22 mmol, 0.055 g) and HB(C_6F_5)$_2$ (0.22 mmol, 0.076 g) and the resulting solution was stirred at room temperature for 1 h. All volatiles were removed under reduced pressure, the residue extracted with *n*-hexane, the extracts combined, concentrated, and stored at -20 ºC, yielding an off-white microcrystalline solid . Yield: 0.12 g (93%). Anal. Calc. for $C_{26}H_{21}BF_{10}N_4$, obtained (calculated): C 53.31 (52.91), H 3.58 (3.59), N 9.21 (9.49). ^{1}H NMR (300 MHz, $CDCl_3$): δ 7.11 (1H, m, NC*H*N), 5.99 (1H, s, C$H_{pyrazole}$), 5.82 (1H, s, C$H_{pyrazole}$), 5.78 (1H, m, =CH_{allyl}), 5.12-4.90 (2H, m, =CH_{2allyl}), 4.77 (1H, vbr, *H*B(C_6F_5)$_2$), 3.57 (1H, m, CH_{2allyl}), 2.70 (1H, m, CH_{2allyl}), 2.20 (3H, s, C$H_{3pyrazole}$), 2.02 (3H, s, C$H_{3pyrazole}$), 1.97 (3H, s, C$H_{3pyrazole}$), 1.93 (3H, s, C$H_{3pyrazole}$). ^{13}C{^{1}H} NMR (75 MHz, $CDCl_3$): δ 151.5 (*C*$Me_{pyrazole}$), 149.8 (m, HB(*C*$_6F_5$)$_2$), 148.4 (*C*$Me_{pyrazole}$), 146.7 (m, HB(*C*$_6F_5$)$_2$), 145.7 (*C*$Me_{pyrazole}$), 141.5 (m, HB(*C*$_6F_5$)$_2$), 141.2 (*C*$Me_{pyrazole}$), 138.8 (m, HB(*C*$_6F_5$)$_2$), 138.3 (m, HB(*C*$_6F_5$)$_2$), 135.7 (m, HB(*C*$_6F_5$)$_2$), 131.7 (=CH_{allyl}), 119.6 (=*C*H_{2allyl}), 111.0 (*C*$H_{pyrazole}$), 107.5 (*C*$H_{pyrazole}$), 72.0 (N*C*HN), 36.9 (CH_{2allyl}), 14.4 (C$H_{3pyrazole}$), 13.8 (C$H_{3pyrazole}$), 12.3 (C$H_{3pyrazole}$), 10.4 (C$H_{3pyrazole}$). ^{11}B NMR (96 MHz, $CDCl_3$): δ -15.0 (br, H*B*(C_6F_5)$_2$). ^{19}F NMR (282 MHz, $CDCl_3$): δ -134.2 (2F, m, *ortho*-HB(C_6*F*$_5$)$_2$), -134.8 (2F, m, *ortho*-HB(C_6*F*$_5$)$_2$), -157.9 (2F, t, *para*-HB(C_6*F*$_5$)$_2$, $^3J_{FF}$ = 19.7 Hz), -163.2 to -164.1 (4F, m, *meta*-HB(C_6*F*$_5$)$_2$).

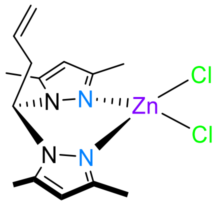

Synthesis of [(L_{allyl})$ZnCl_2$] (2): Dichloromethane was added to a solid mixture of **L_{allyl}** (0.82 mmol, 0.20 g) and $ZnCl_2$ (0.90 mmol, 0.12 g) and the resulting cloudy solution was stirred at room temperature overnight. All volatiles were removed under reduced pressure, the residue redissolved in minimal dichloromethane and the solution was filtered onto three-fold *n*-hexane under magnetic stirring, thus precipitating a white powder. The supernatant was filtered off and the white powder was dried under vacuum. Crystals suitable for X-ray diffraction were obtained from slow evaporation of a saturated dichloromethane solution into *n*-hexane. Yield: 0.27 g (86%). Anal. Calc. for $C_{14}H_{20}Cl_2N_4Zn$•1.25CH_2Cl_2, obtained (calculated): C 37.47 (37.63), H 4.63 (4.66), N 11.96 (11.51). ^{1}H NMR (300 MHz, $CDCl_3$): δ 6.19 (1H, t, NC*H*N, $^3J_{HH}$ = 9.0 Hz), 5.77 (2H, s, C$H_{pyrazole}$), 5.34 (1H, m, =CH_{allyl}), 5.18-5.00 (2H, m, =CH_{2allyl}), 3.16 (2H, t, CH_{2allyl}, $^3J_{HH}$ = 9.0 Hz), 2.48 (6H, s, C$H_{3pyrazole}$), 2.45 (6H, s, C$H_{3pyrazole}$). ^{13}C{^{1}H} NMR (75 MHz, $CDCl_3$): δ 154.3 (*C*$Me_{pyrazole}$), 143.6 (*C*$Me_{pyrazole}$), 128.8 (=CH_{allyl}), 122.4 (=*C*H_{2allyl}), 107.8 (*C*$H_{pyrazole}$), 66.9 (N*C*HN), 41.3 (CH_{2allyl}), 14.1 (C$H_{3pyrazole}$), 11.7 (C$H_{3pyrazole}$).

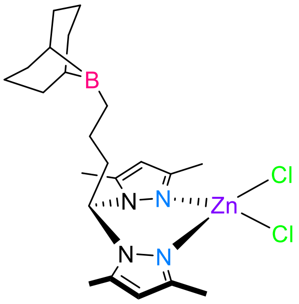

Synthesis of [(1a)ZnCl$_2$] (3a): *Method a:* Dichloromethane was added to a solid mixture of **2** (1.7 mmol, 0.65 g) and the 9-BBN dimer (1.3 mmol, 0.31 g) and the resulting solution was stirred at room temperature for 2 h. All volatiles were removed under reduced pressure, the residue redissolved in minimal dichloromethane and the solution was filtered onto three-fold *n*-hexane under magnetic stirring, thus precipitating a white powder. The supernatant was filtered off and the white powder was dried under vacuum. Yield: 0.58 g (67%). *Method b:* Dichloromethane was added to a mixture of **1a** (1.0 mmol, 0.367 g) and $ZnCl_2$ (1.1 mmol, 0.150 g) and the resulting cloudy solution was stirred for 16 h at room temperature. The solvent was evaporated to dryness and dissolved in minimal dichloromethane and filtered to 3-fold *n*-hexane, precipitating a white powder. Yield: 0.41 g (81%). Crystals suitable for X-ray diffraction were obtained from slow diffusion of *n*-pentane onto a saturated dichloromethane solution. Anal. Calc. for $C_{22}H_{35}BCl_2N_4Zn{\bullet}0.6CH_2Cl_2$, obtained (calculated): C 49.33 (49.03), H 6.76 (6.59), N 9.63 (10.12). ^{1}H NMR (300 MHz, $CDCl_3$): δ 6.16 (1H, t, NC*H*N, $^3J_{HH}$ = 9.0 Hz), 6.04 (2H, s, C$H_{pyrazole}$), 2.60-2.47 (8H, m, B-CH_2C$H_2$$CH_2$-C + C$H_{3pyrazole}$), 2.45 (6H, s, C$H_{3pyrazole}$), 1.88-1.08 (16H, m, C$H_{9\text{-}BBN}$ + C$H_{2,9\text{-}BBN}$ + B-C$H_2$$CH_2$$CH_2$-C + B-$CH_2$$CH_2CH_2$-C). ^{13}C{^{1}H} NMR (75 MHz, $CDCl_3$): δ 154.1 (*C*$Me_{pyrazole}$), 142.6 (*C*$Me_{pyrazole}$), 107.8 (*C*$H_{pyrazole}$), 67.4 (N*C*HN), 40.2 (B-$CH_2$$CH_2CH_2$-C), 33.2 (*C*$H_{2,9\text{-}BBN}$), 31.0 (*C*$H_{9\text{-}BBN}$), 23.3 (B-C$H_2$$CH_2$$CH_2$-C), 23.2 (*C*$H_{2,9\text{-}BBN}$), 20.1 (B-$CH_2CH_2$$CH_2$-C), 14.1 (C$H_{3pyrazole}$), 11.6 (C$H_{3pyrazole}$). ^{11}B NMR (96 MHz, $CDCl_3$): δ 88.6 (br, 9-BBN).

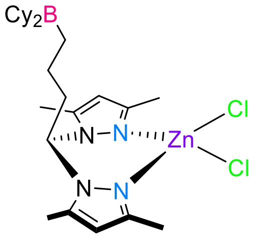

Synthesis of [(1b)ZnCl$_2$] (3b): *Method a:* Dichloromethane was added to a solid mixture of **2** (1.00 mmol, 0.380 g) and dicyclohexylborane (1.20 mmol, 0.214 g) and the resulting solution was stirred at room temperature for 2 h. All volatiles were removed under reduced pressure, the residue redissolved in minimal dichloromethane and the solution was filtered onto three-fold *n*-hexane under magnetic stirring, thus precipitating a white powder. The supernatant was filtered off and the white powder was dried under vacuum. Yield: 0.53 g (95%). *Method b:* Dichloromethane was added to a mixture of **1b** (1.0 mmol, 0.423 g) and ZnCl$_2$ (1.1 mmol, 0.150 g) and the resulting cloudy solution was stirred for 16 h at room temperature. The solvent was evaporated to dryness and dissolved in minimal dichloromethane and filtered to 3-fold *n*-hexane, precipitating a white powder. Yield: 0.35 g (63%). Anal. Calc. for $C_{26}H_{43}BCl_2N_4Zn$•$0.5CH_2Cl_2$, obtained (calculated): C 52.63 (52.94), H 7.21 (7.38), N 8.97 (9.32). ^{1}H NMR (300 MHz, CDCl$_3$): δ 6.14 (1H, t, NC*H*N, $^3J_{HH}$ = 7.5 Hz), 6.05 (2H, s, C*H*$_{pyrazole}$), 2.54 (6H, s, C*H*$_{3pyrazole}$), 2.46 (6H, s, C*H*$_{3pyrazole}$), 1.72-1.02 (25H, m, C*H*$_{BCy2}$ + C*H*$_{2BCy2}$ + B-C*H*$_2$CH$_2$CH$_2$-C + B-CH$_2$C*H*$_2$CH$_2$-C + B-CH$_2$CH$_2$C*H*$_2$-C). ^{13}C{^{1}H} NMR (75 MHz, CDCl$_3$): δ 154.0 (*C*Me$_{pyrazole}$), 142.1 (*C*Me$_{pyrazole}$), 107.4 (*C*H$_{pyrazole}$), 67.4 (N*C*HN), 40.3 (B-CH$_2$CH$_2$*C*H$_2$-C), 35.7 (*C*$_{BCy2}$), 27.5 (*C*$_{BCy2}$), 27.0 (*C*$_{BCy2}$), 23.8 (B-*C*H$_2$CH$_2$CH$_2$-C), 19.6 (B-CH$_2$*C*H$_2$CH$_2$-C), 14.0 (C*H*$_{3pyrazole}$), 11.5 (C*H*$_{3pyrazole}$). ^{11}B NMR (96 MHz, CDCl$_3$): δ 85.0 (br, *B*Cy$_2$).

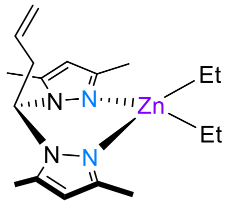

Synthesis of [(L_{allyl})ZnEt$_2$] (4): ZnEt$_2$ (0.75 mmol, 0.75 mL of a 1 M solution in hexanes) was added to a toluene solution of $\mathbf{L_{allyl}}$ (0.73 mmol, 0.18 g) and the mixture was stirred for 1 h at room temperature. The solution was concentrated, double layered with three-fold *n*-hexane and stored at -20 ºC for 1h, precipitating a tan solid. The supernatant was decanted off and solid dried under vacuum. Yield: 0.18 g (66%). This complex is extremely sensitive to air and to recrystallization. Because of this, despite many attempts, unsuitable elemental analysis results were obtained. ^{1}H NMR (300 MHz, THF-d_8): δ 6.24 (1H, t, NC*H*N, $^3J_{HH}$ = 9.0 Hz), 5.81 (2H, s, C$H_{pyrazole}$), 5.47 (1H, m, =CH_{allyl}), 5.15-4.86 (2H, m, =CH_{2allyl}), 3.26 (2H, t, CH_{2allyl}, $^3J_{HH}$ = 9.0 Hz), 2.31 (6H, s, C$H_{3pyrazole}$), 2.19 (6H, s, C$H_{3pyrazole}$), 0.93 (6H, t, ZnCH$_2$CH_3, $^3J_{HH}$ = 9.0 Hz), -0.07 (4H, q, ZnCH_2CH$_3$, $^3J_{HH}$ = 6.0 Hz). ^{13}C{^{1}H} NMR (75 MHz, THF-d_8): δ 148.8 (*C*Me$_{pyrazole}$), 139.7 (*C*Me$_{pyrazole}$), 132.3 (=CH_{allyl}), 118.2 (=*C*H$_{2allyl}$), 105.7 (*C*H$_{pyrazole}$), 68.9 (N*C*HN), 38.7 (CH_{2allyl}), 12.7 (C$H_{3pyrazole}$), 12.1 (C$H_{3pyrazole}$), 10.1 (ZnCH$_2$*C*H$_3$), 2.2 (Zn*C*H$_2$CH$_3$).

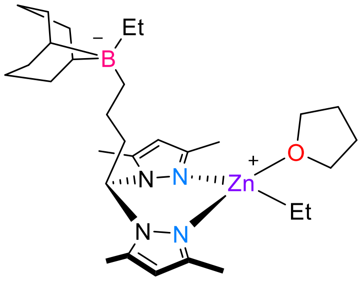

Synthesis of [Et(1a)ZnEt(THF)] (5): $ZnEt_2$ (1.0 mmol, 1.0 mL of a 1 M solution in hexanes) was added to a THF solution of **1a** (1.0 mmol, 0.367 g) and the mixture was stirred for 1 h at room temperature. The solution was concentrated, double layered with three-fold *n*-hexane and stored at -20 ºC for 1h, precipitating a colorless oil. The supernatant was decanted off and the oil dried under vacuum, upon which a white foamy solid gradually formed. Yield: 0.11 g (23%). This complex is extremely sensitive to air and to recrystallization. Because of this, despite many attempts, unsuitable elemental analysis results were obtained. ^{1}H NMR (300 MHz, THF-d_8): δ 6.60 (1H, t, NC*H*N, $^{3}J_{HH}$ = 7.5 Hz), 6.18 (2H, s, C$H_{pyrazole}$), 3.62 (4H, m, CH_{2THF}), 2.56 (6H, s, C$H_{3pyrazole}$), 2.33 (6H, s, C$H_{3pyrazole}$), 2.17 (2H, q, C$H_2$$CH_2$$CH_2$, $^{3}J_{HH}$ = 7.5 Hz), 2.03-1.74 (10H, m, C$H_{9\text{-BBN}}$ + C$H_{2,9\text{-BBN}}$ + CH_{2THF}), 1.48-1.29 (8H, m, CH_2C$H_2$$CH_2$ + $CH_2$$CH_2CH_2$ + C$H_{2,9\text{-BBN}}$), 0.65-0.35 (6H, m, ZnCH_2CH_3 + BCH_2CH_3), 0.29 to -0.09 (8H, m, C$H_{2,9\text{-BBN}}$ + ZnC$H_2$$CH_3$ + BC$H_2$$CH_3$). $^{13}C\{^{1}H\}$ NMR (75 MHz, THF-d_8): δ 153.3 (*C*$Me_{pyrazole}$), 145.1 (*C*$Me_{pyrazole}$), 107.8 (*C*$H_{pyrazole}$), 68.7 (N*C*HN), 44.6 (B-$CH_2$$CH_2CH_2$-C), 34.62 (*C*$H_{2,9\text{-BBN}}$), 34.48 (*C*$H_{9\text{-BBN}}$), 28.49 (B-C$H_2$$CH_2$$CH_2$-C), 26.54 (*C*$H_{2,9\text{-BBN}}$), 22.37 (B-$CH_2CH_2$$CH_2$-C), 13.45 (C$H_{3pyrazole}$), 13.17 (B$CH_2CH_3$), 11.30 (C$H_{3pyrazole}$), 11.20 (Zn$CH_2CH_3$), 8.96 (B$CH_2$$CH_3$), -1.52 (ZnC$H_2$$CH_3$). ^{11}B NMR (96 MHz, THF-d_8): δ -17.9 (s, 9-BBN).

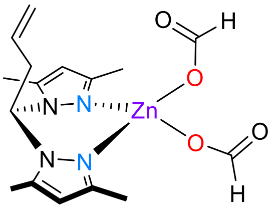

Synthesis of [(L_{allyl})Zn(OCHO)$_2$] (7): *Method a*: A mixture of complex **2** (1.0 mmol, 0.38 g) and $KHBEt_3$ (2.2 mmol, 0.30 g) were treated with THF at -78 ºC, warmed up to room temperature and stirred for 30 min, forming a cloudy solution. The solution was filtered and degassed by three freeze-pump-thaw cycles. The degassed solution was saturated with CO_2 (atmosphere pressure) and stirred overnight. The solution was concentrated, double layered with *n*-hexane and cooled to -20 ºC, precipitating a white powder. The powder was redissolved in minimal dichloromethane, the solution filtered to three-fold *n*-hexane with stirring, reprecipitating a white powder. Yield: 0.22 g (54%). *Method b*: $ZnEt_2$ (1.1 mmol, 1.1 mL of a 1 M solution in hexanes) was added to a toluene solution of $\mathbf{L_{allyl}}$ (1.0 mmol, 0.25 g) and the mixture was stirred for 1 h at room temperature. Formic acid (2.1 mmol, 0.075 mL) was added to the solution and the resulting cloudy solution was stirred until effervescence was no longer observed. Three-fold *n*-hexane was added to the mixture, precipitating a white powder. The supernatant was decanted off and the powder was washed with toluene and with *n*-hexane and redissolved in minimal dichloromethane. The solution was filtered to three-fold *n*-hexane with stirring, reprecipitating a white powder. Crystals suitable for X-ray diffraction were obtained by slow evaporation of a dichloromethane solution into *n*-hexane. Yield: 0.15 g (35%). Anal. Calc. for $C_{16}H_{22}N_4O_4Zn{\bullet}3CH_2Cl_2$, obtained (calculated): C 34.69 (34.87), H 4.48 (4.31), N 8.36 (8.56). ^{1}H NMR (300 MHz, $CDCl_3$): δ 8.32 (2H, s, OC*H*O), 6.08 (1H, t, NC*H*N, $^3J_{HH}$ = 9.0 Hz), 6.00 (2H, s, C$H_{pyrazole}$), 5.29 (1H, m, =CH_{allyl}), 5.17-5.03 (2H, m, =CH_{2allyl}), 3.10 (2H, t, CH_{2allyl}, $^3J_{HH}$ = 9.0 Hz), 2.42 (6H, s, C$H_{3pyrazole}$), 2.40 (6H, s, C$H_{3pyrazole}$). ^{13}C{^{1}H} NMR (75 MHz, $CDCl_3$): δ 167.0 (O*C*HO), 154.3 (*C*$Me_{pyrazole}$), 142.3 (*C*$Me_{pyrazole}$), 128.9 (=CH_{allyl}), 122.4 (=*C*H_{2allyl}), 107.5 (*C*$H_{pyrazole}$), 66.8 (N*C*HN), 40.9 (CH_{2allyl}), 13.4 (C$H_{3pyrazole}$), 11.4 (C$H_{3pyrazole}$).

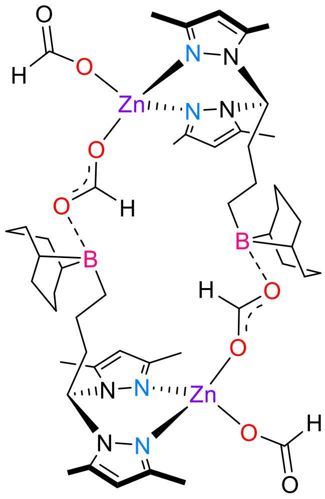

Synthesis of [(1a)Zn(OCHO)$_2$]$_2$ (8): *Method a*: A mixture of complex **3a** (0.85 mmol, 0.43 g) and $KHBEt_3$ (1.7 mmol, 0.23 g) were treated with THF at -78 ºC, warmed up to room temperature and stirred for 30 min, forming a cloudy solution. The solution was filtered and degassed by three freeze-pump-thaw cycles. The degassed solution was saturated with CO_2 (atmosphere pressure) and stirred overnight. The solution was concentrated, double layered with *n*-hexane and cooled to -20 ºC, precipitating a white powder. Yield: 0.186 g (42%). *Method b*: $ZnEt_2$ (1.0 mmol, 1.0 mL of a 1 M solution in hexanes) was added to a THF solution of **1a** (1.0 mmol, 0.37 g) and the mixture was stirred for 1 h at room temperature, resulting in a cloudy solution. THF was added to the mixture until complete dissolution and formic acid (2.0 mmol, 0.075 mL) was added to the solution and the resulting cloudy solution was stirred until effervescence was no longer observed. Three-fold *n*-hexane was added to the mixture, precipitating a white powder. The supernatant was decanted off and the powder was washed with toluene and with *n*-hexane and extracted in refluxing dichloromethane. The combined extracts were concentrated and filtered to three-fold *n*-hexane with stirring, reprecipitating a white powder. Yield: 0.41 g (78%). Anal. Calc. for $C_{48}H_{76}B_2N_8O_8Zn_2$•$CH_2Cl_2$, obtained (calculated): C 52.36 (52.06), H 7.32 (6.95), N 9.53 (9.91). ^{1}H NMR (300 MHz, $CDCl_3$): δ 8.33 (4H, s, OC*H*O), 6.25 (2H, t, NC*H*N, $^3J_{HH}$ = 9.0 Hz), 6.00 (4H, s, C*H*$_{pyrazole}$), 2.43 (12H, s, C*H*$_{3pyrazole}$), 2.34 (12H, s, C*H*$_{3pyrazole}$), 2.20 (4H, q, B-CH_2CH_2C*H*$_2$-C, $^3J_{HH}$ = 8.0 Hz), 1.76 (4H, m, C*H*$_{9\text{-BBN}}$), 1.49 (12H, m, C*H*$_{2,9\text{-BBN}}$), 1.31 (4H, m, C*H*$_{2,9\text{-BBN}}$), 0.76 (4H, br, B-CH_2C*H*$_2$$CH_2$-C), 0.52 (4H, m, C*H*$_{2,9\text{-BBN}}$), 0.44 (4H, m, B-C*H*$_2$$CH_2CH_2$-C). ^{13}C{^{1}H} NMR (75 MHz, $CDCl_3$): δ 170.1 (O*C*HO), 153.8 (*C*Me$_{pyrazole}$), 142.7 (*C*Me$_{pyrazole}$), 107.8 (*C*H$_{pyrazole}$), 66.8 (N*C*HN), 40.9 (B-CH_2CH_2*C*H$_2$-C), 31.9 (*C*H$_{2,9\text{-BBN}}$), 25.8 (*C*H$_{9\text{-BBN}}$), 25.1 (*C*H$_{2,9\text{-BBN}}$), 22.9 (B-*C*H$_2$$CH_2CH_2$-C), 19.4 (B-$CH_2$*C*H$_2$$CH_2$-C), 13.5 (*C*H$_{3pyrazole}$), 11.4 (*C*H$_{3pyrazole}$). ^{11}B NMR (96 MHz, $CDCl_3$): δ -21.1 (br, 9-BBN).

General procedure for catalytic CO_2 hydroboration: Inside a dinitrogen-filled glovebox, the desired complex (typically 0.01 mmol) and $KHBEt_3$ (typically 0.02 mmol) were weighed together in a 5 mL vial and 0.5 mL of deuterated solvent was added. In a separate 5 mL vial, HBPin (typically 1 mmol) and 1,3,5-trimethoxybenzene (typically 1 mmol) were also weighed together. The contents of the two vials were combined and added to a J. Young NMR tube. The resulting mixture was degassed by the freeze-pump-thaw technique, subjected to 1 bar of CO_2, and heated at the desired temperature. After completion of the reaction, the mixture was analyzed by NMR spectroscopy. Representative NMR spectra of the CO_2 hydroboration reactions are presented in Figures S48-S52 of the Supporting Information.

NMR spectra of the compounds

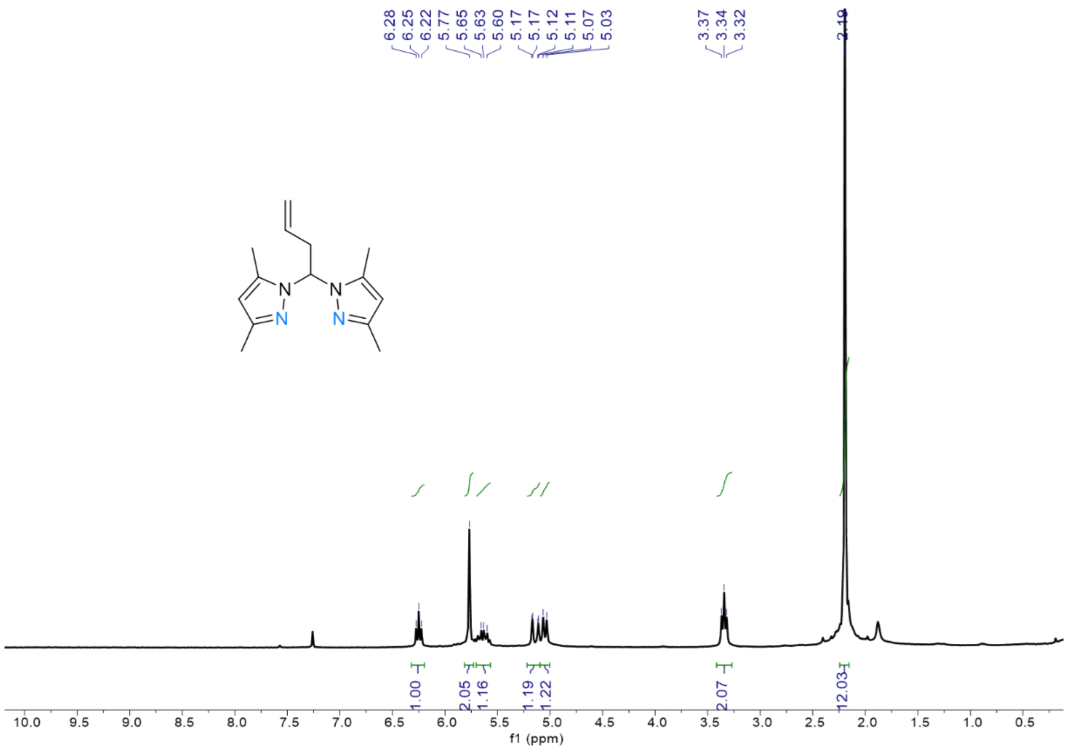

Figure S1 ^{1}H NMR spectrum (300 MHz, $CDCl_3$) of compound $\mathbf{L_{allyl}}$.

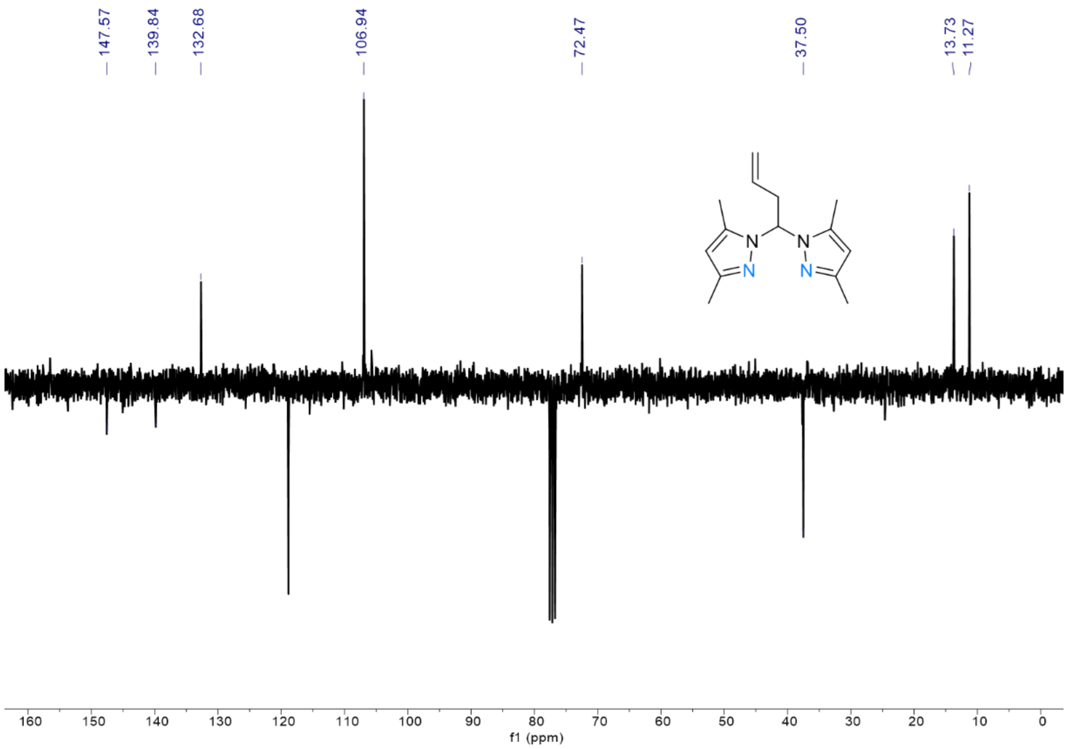

Figure S2 ^{13}C APT NMR spectrum (75 MHz, $CDCl_3$) of compound $\mathbf{L_{allyl}}$.

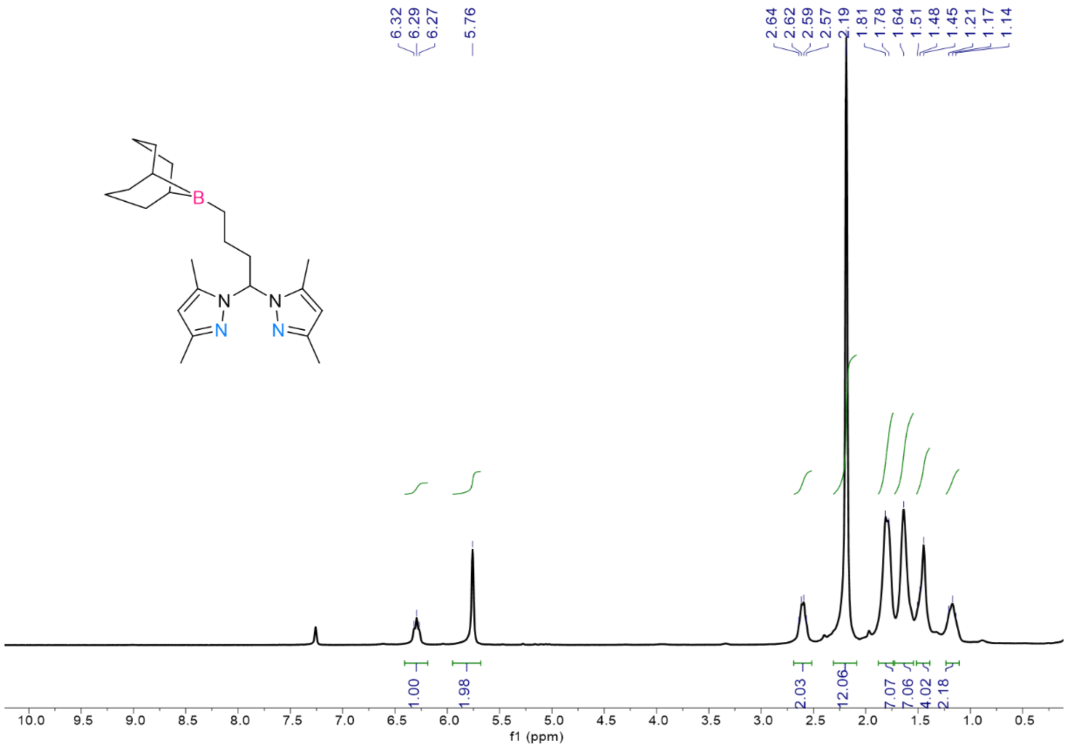

Figure S3 ^{1}H NMR spectrum (300 MHz, $CDCl_3$) of compound **1a**.

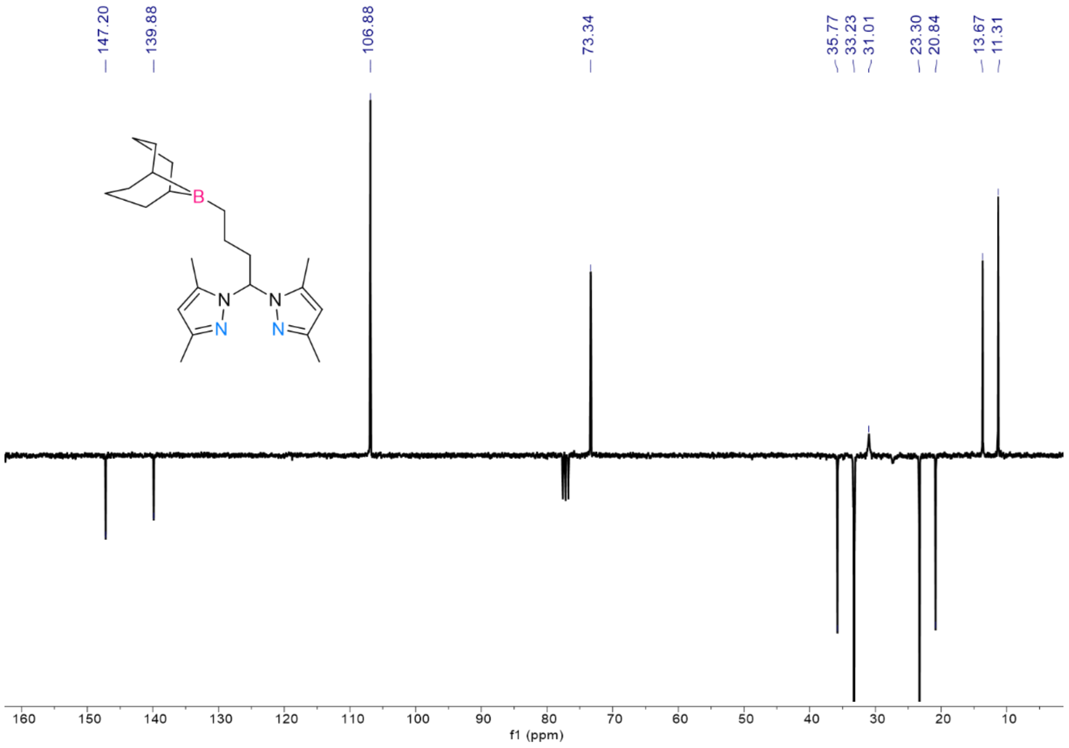

Figure S4 ^{13}C APT NMR spectrum (75 MHz, $CDCl_3$) of compound **1a**.

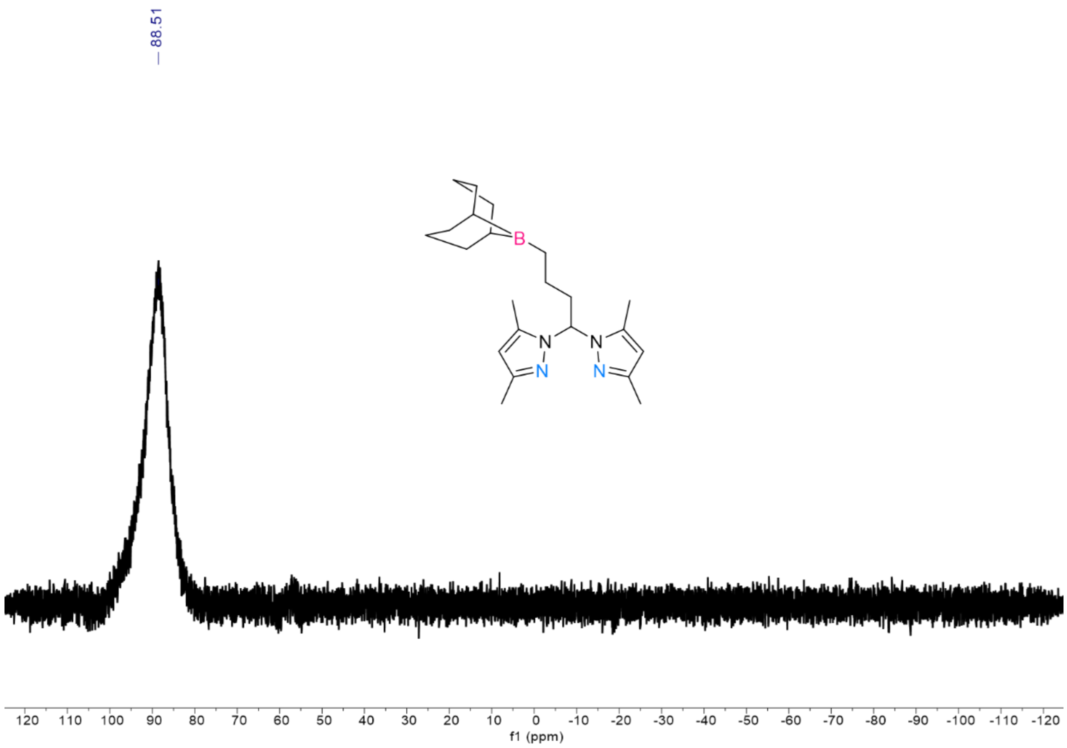

Figure S5 ^{11}B NMR spectrum (96 MHz, $CDCl_3$) of compound **1a**.

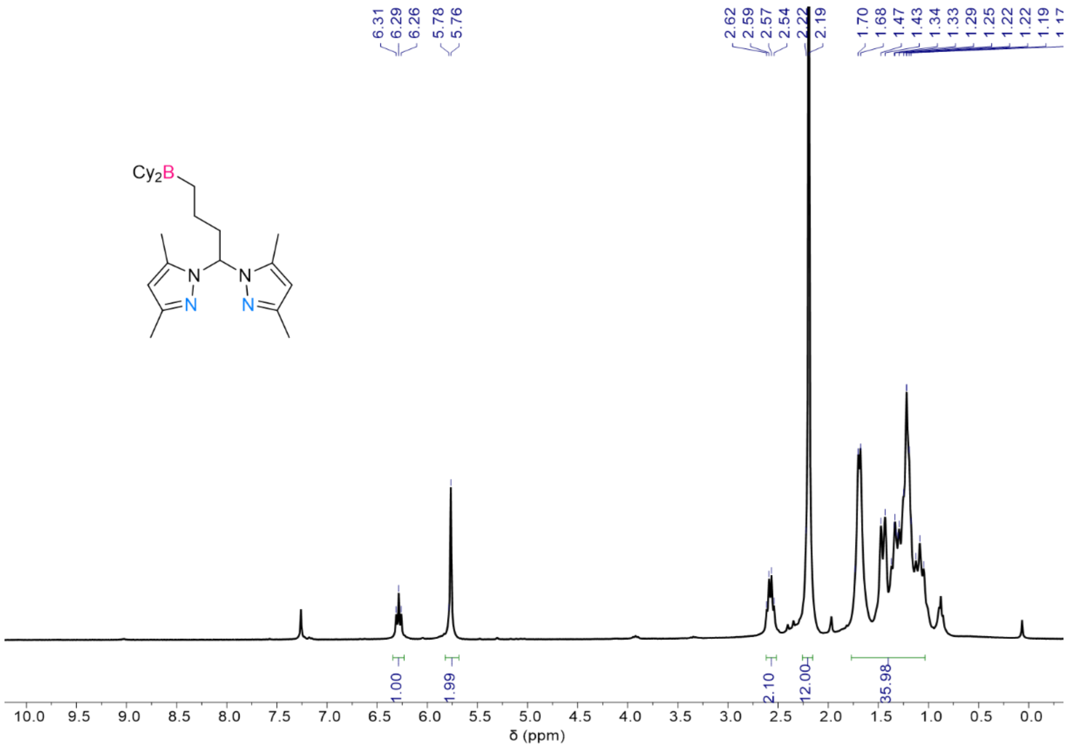

Figure S6 ^{1}H NMR spectrum (300 MHz, $CDCl_3$) of compound **1b**.

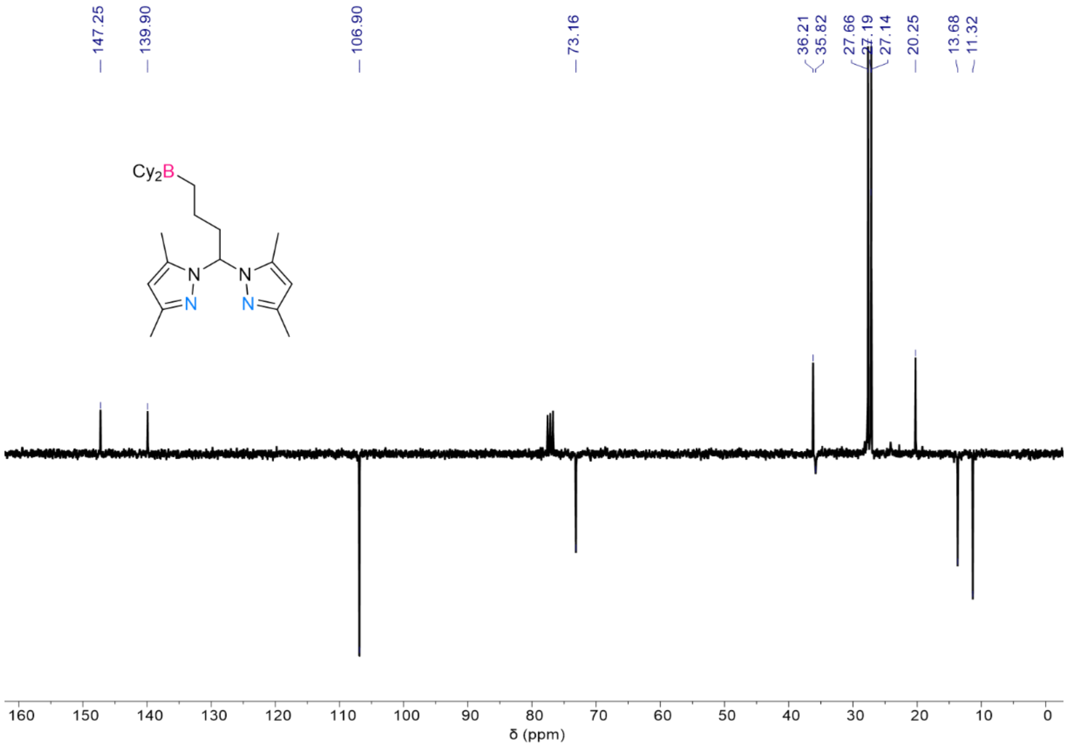

Figure S7 ^{13}C APT NMR spectrum (75 MHz, $CDCl_3$) of compound **1b**.

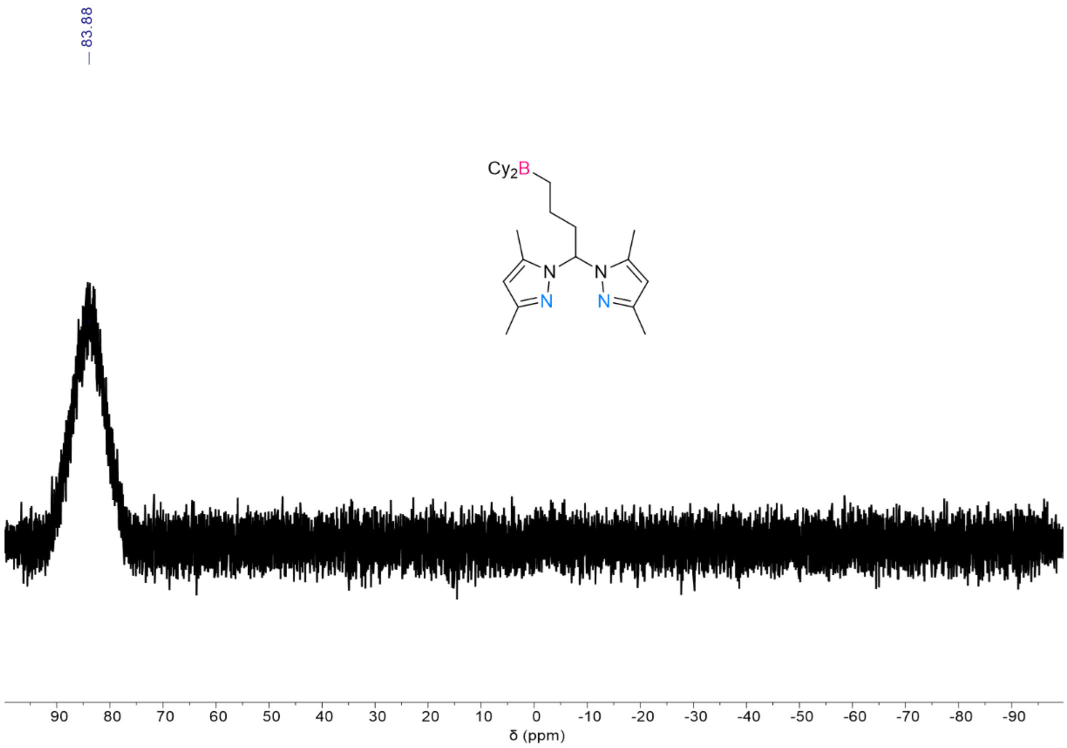

Figure S8 ^{11}B NMR spectrum (96 MHz, $CDCl_3$) of compound **1b**.

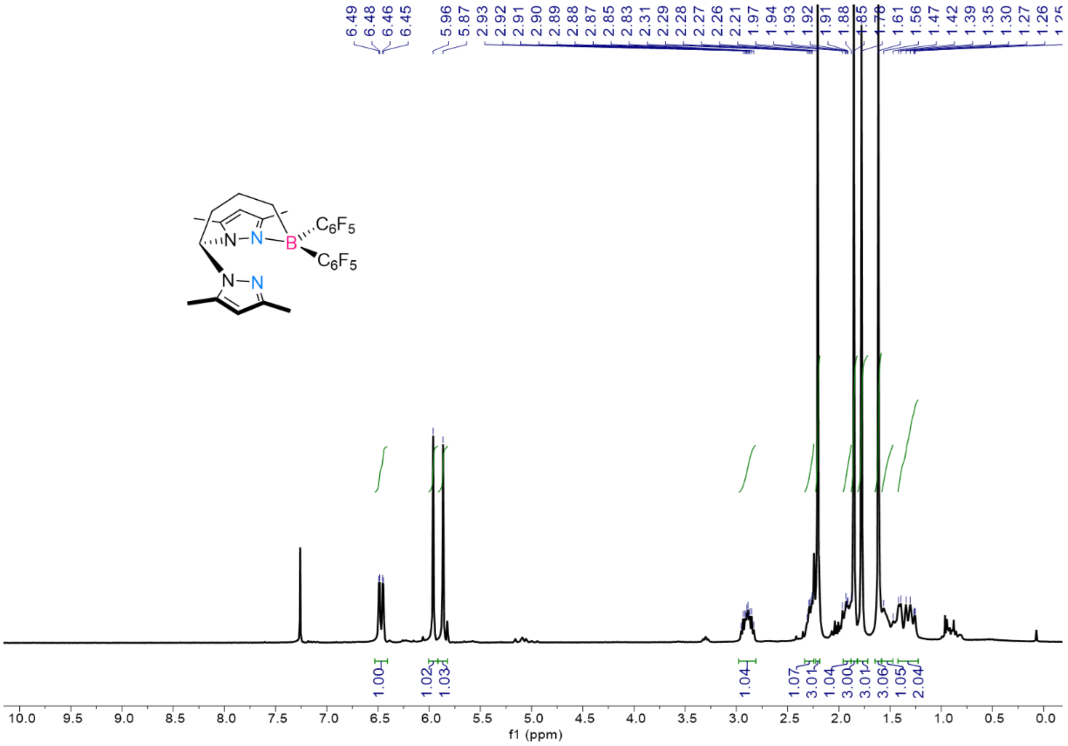

Figure S9 ^{1}H NMR spectrum (300 MHz, $CDCl_3$) of compound **1c**.

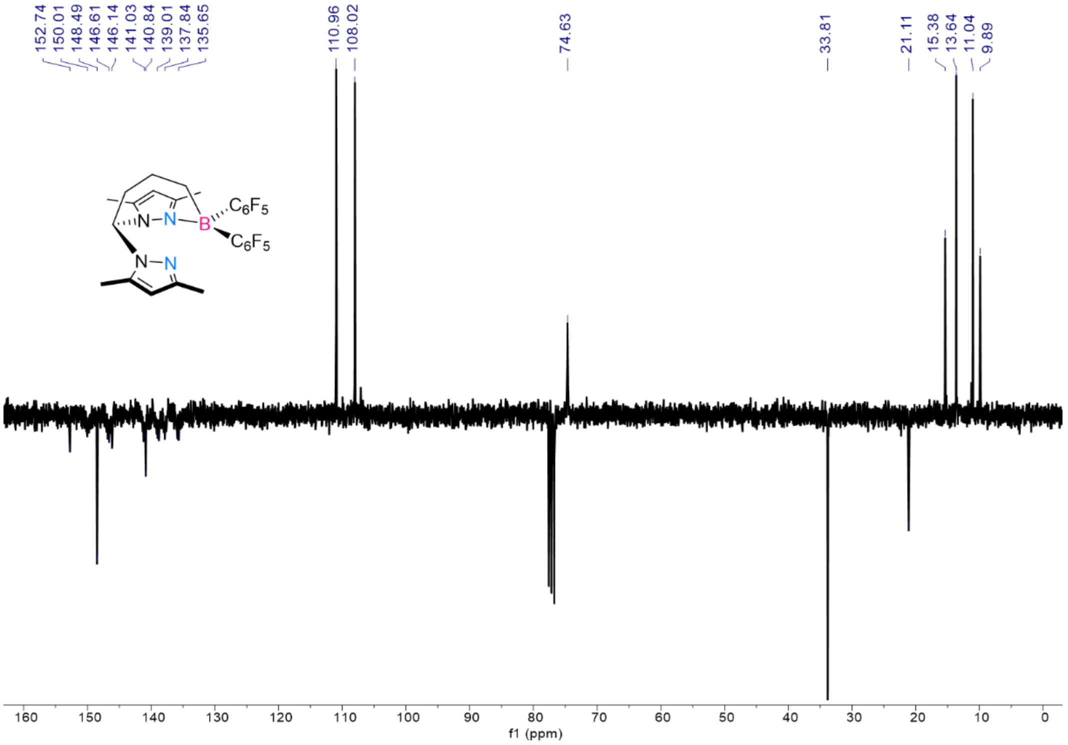

Figure S10 ^{13}C APT NMR spectrum (75 MHz, $CDCl_3$) of compound **1c**.

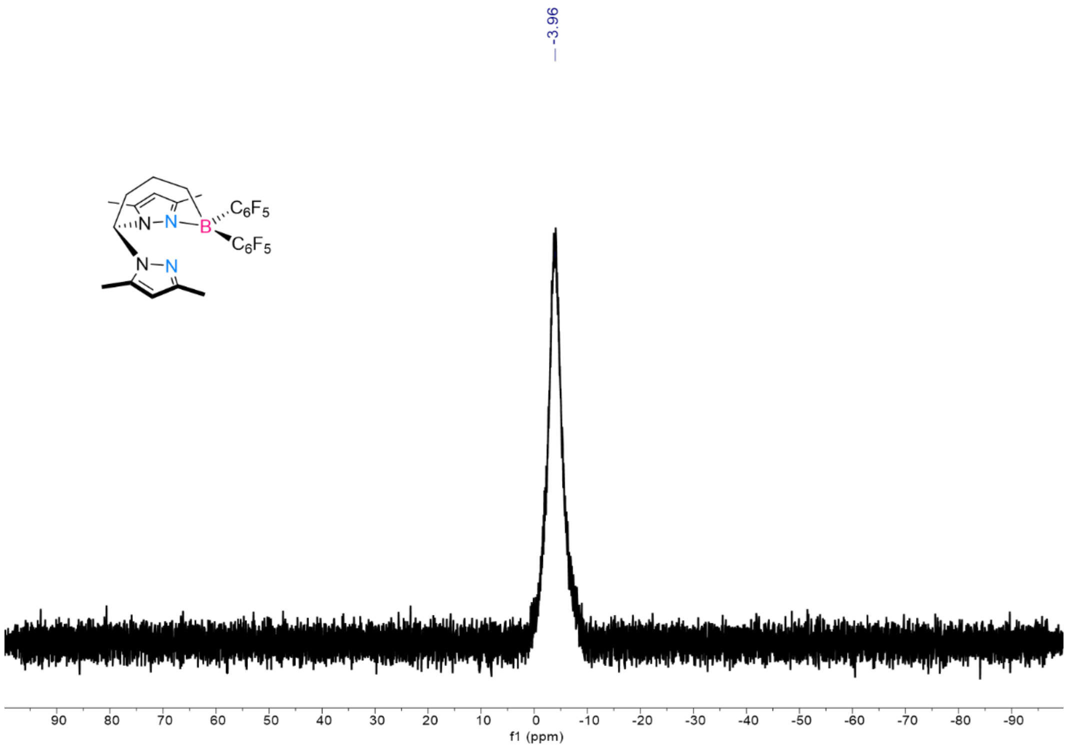

Figure S11 ^{11}B NMR spectrum (96 MHz, $CDCl_3$) of compound **1c**.

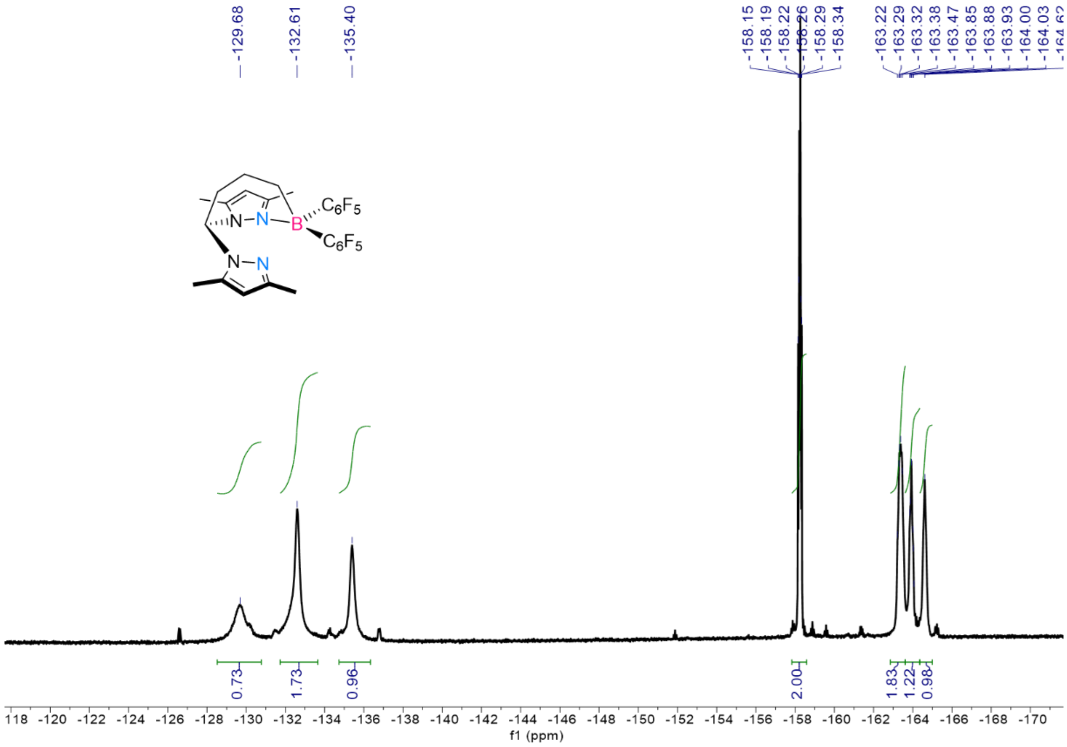

Figure S12 ^{19}F NMR spectrum (96 MHz, $CDCl_3$) of compound **1c**.

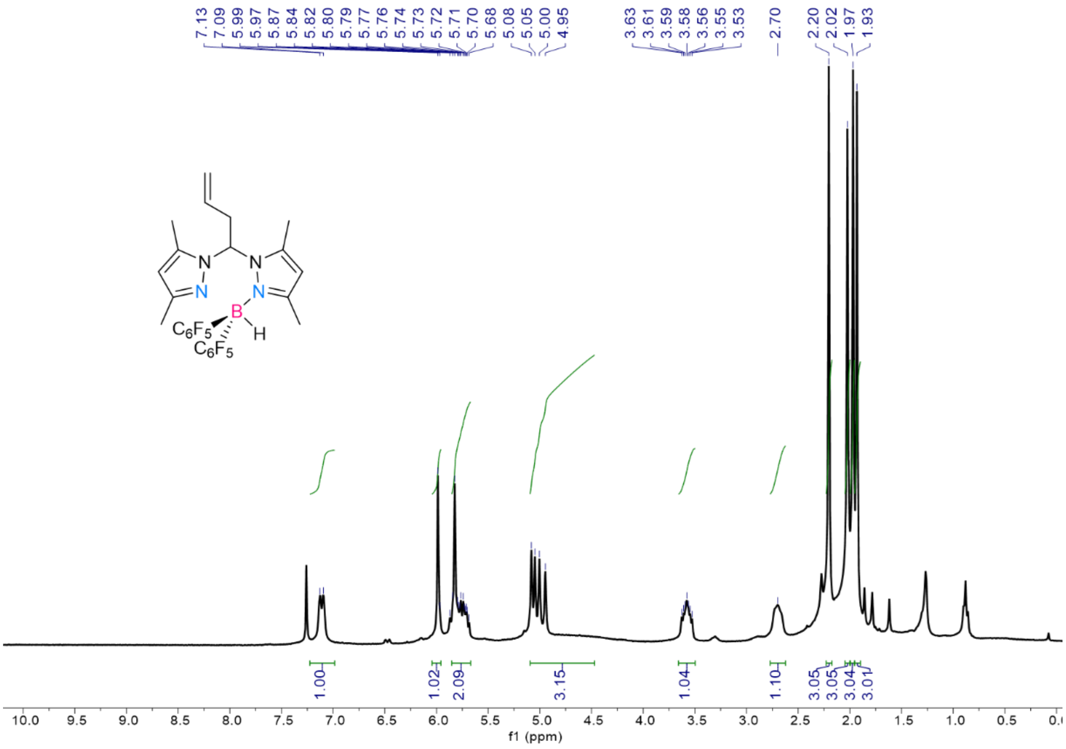

Figure S13 ^{1}H NMR spectrum (300 MHz, $CDCl_3$) of compound **L_{allyl}•HB(C_6F_5)$_2$**.

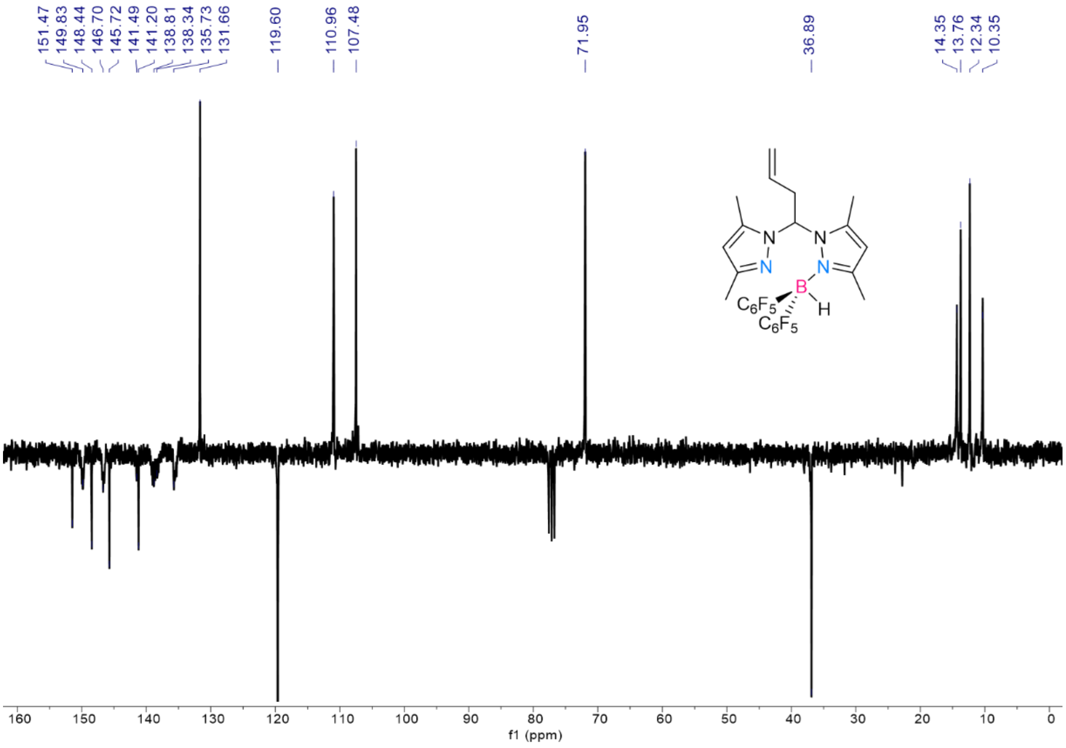

Figure S14 ^{13}C APT NMR spectrum (75 MHz, $CDCl_3$) of compound **L_{allyl}•HB(C_6F_5)$_2$**.

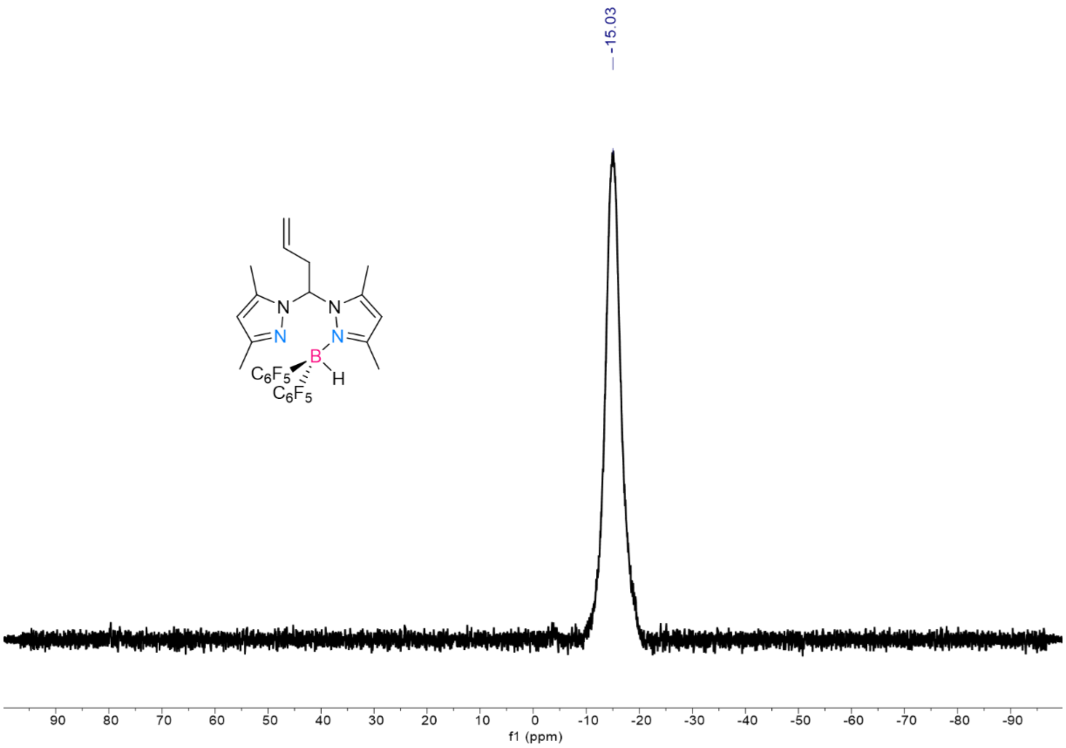

Figure S15 ^{11}B NMR spectrum (96 MHz, $CDCl_3$) of compound **L$_{allyl}$•HB(C_6F_5)$_2$.**

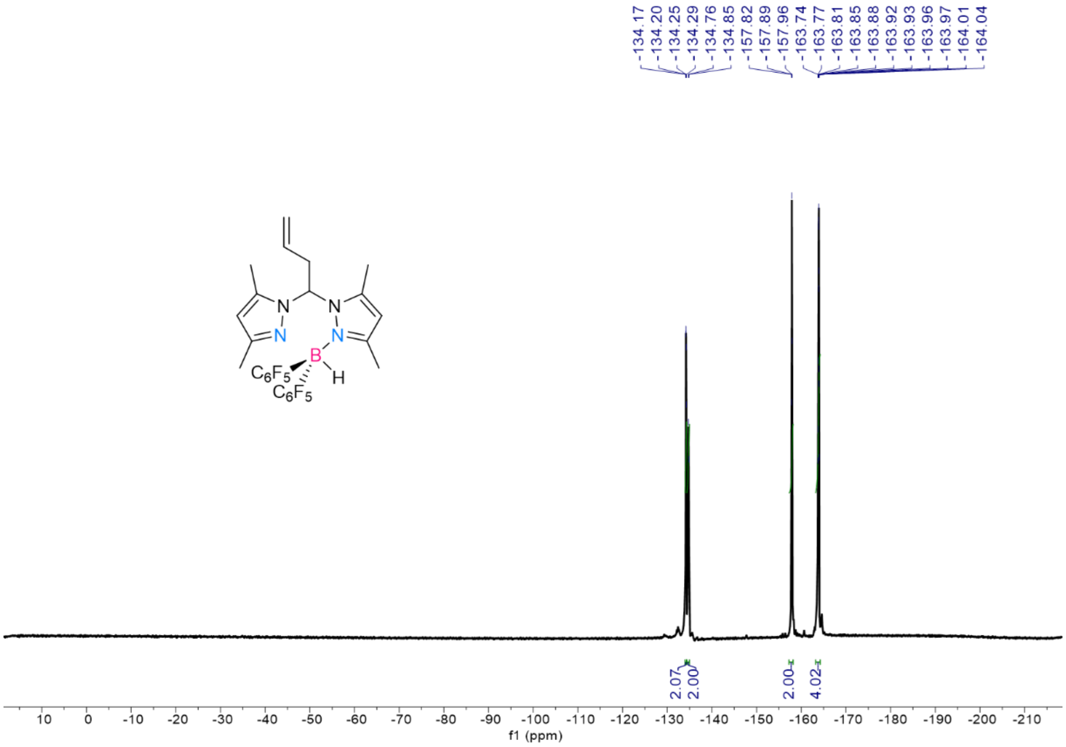

Figure S16 ^{19}F NMR spectrum (96 MHz, $CDCl_3$) of compound **L$_{allyl}$•HB(C_6F_5)$_2$.**

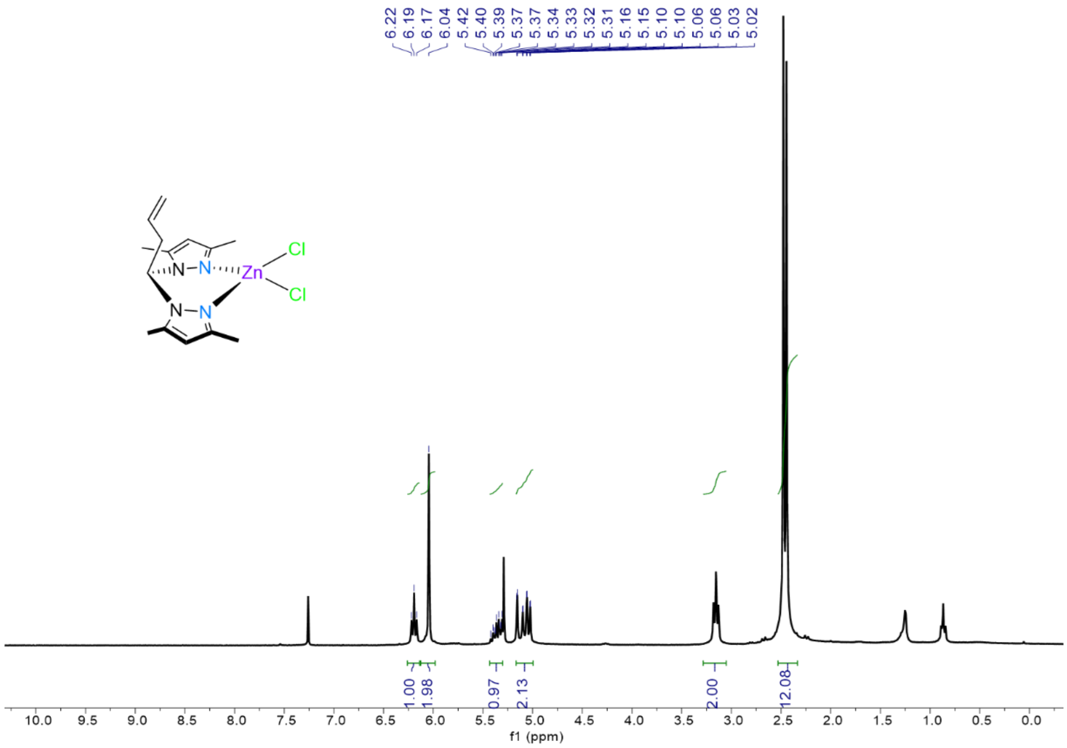

Figure S17 ^{1}H NMR spectrum (300 MHz, $CDCl_3$) of complex **2**.

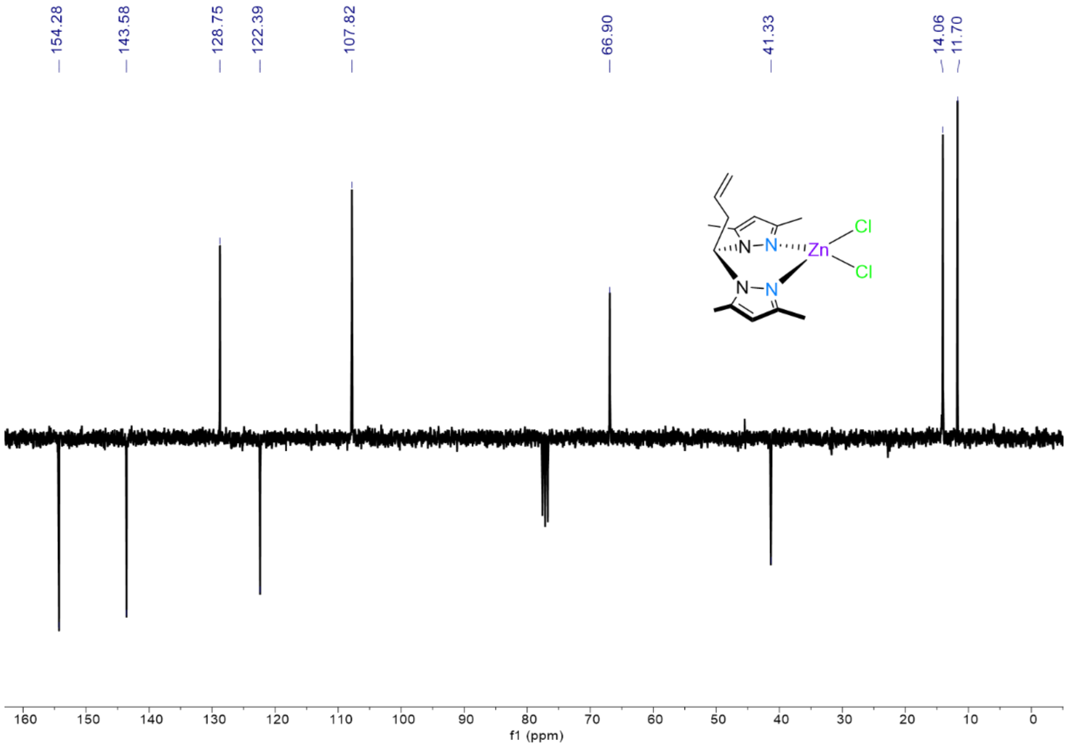

Figure S18 ^{13}C APT NMR spectrum (75 MHz, $CDCl_3$) of complex **2**.

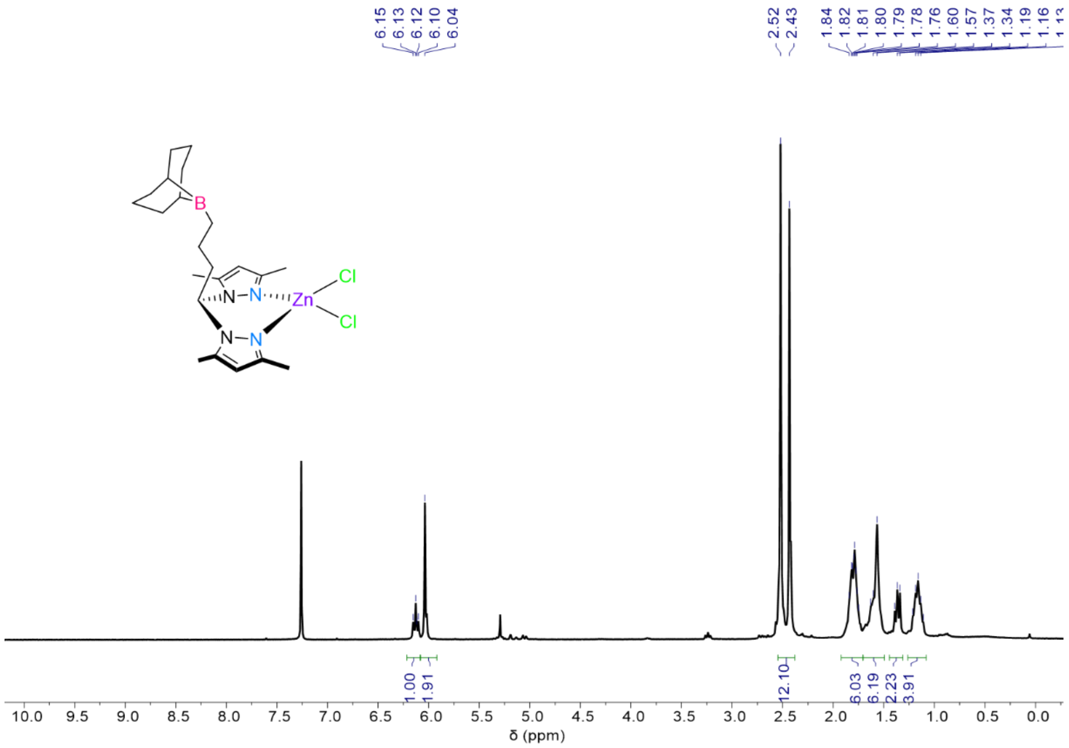

Figure S19 ^{1}H NMR spectrum (300 MHz, $CDCl_3$) of complex **3a**.

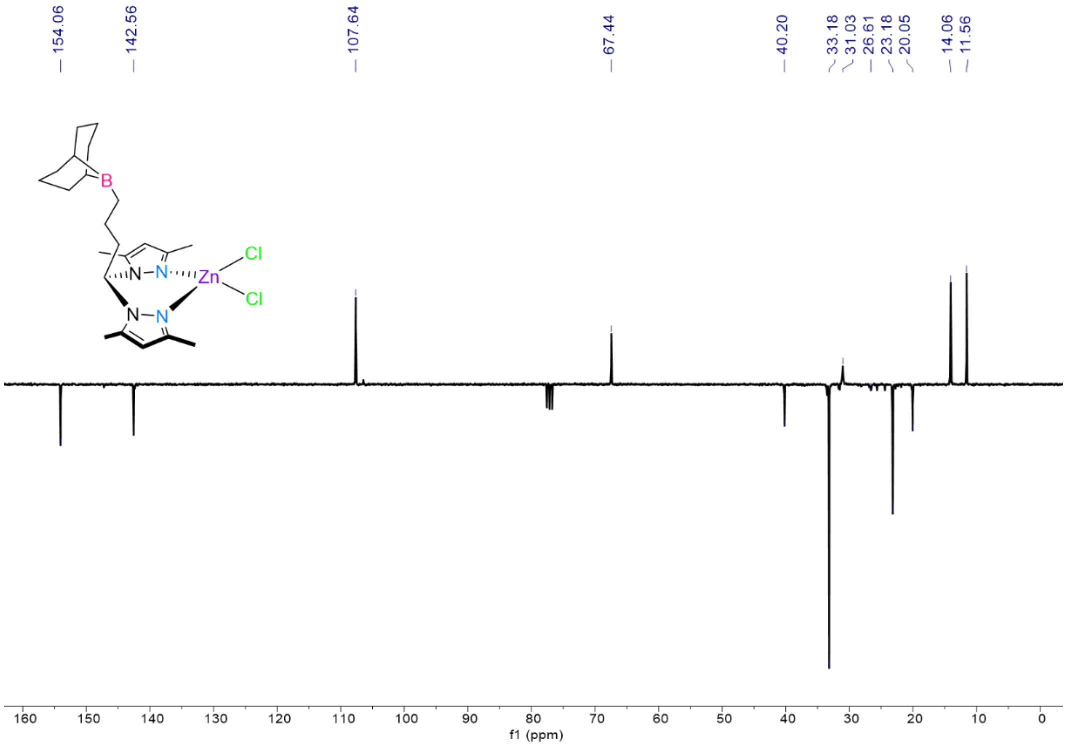

Figure S20 ^{13}C APT NMR spectrum (75 MHz, $CDCl_3$) of complex **3a**.

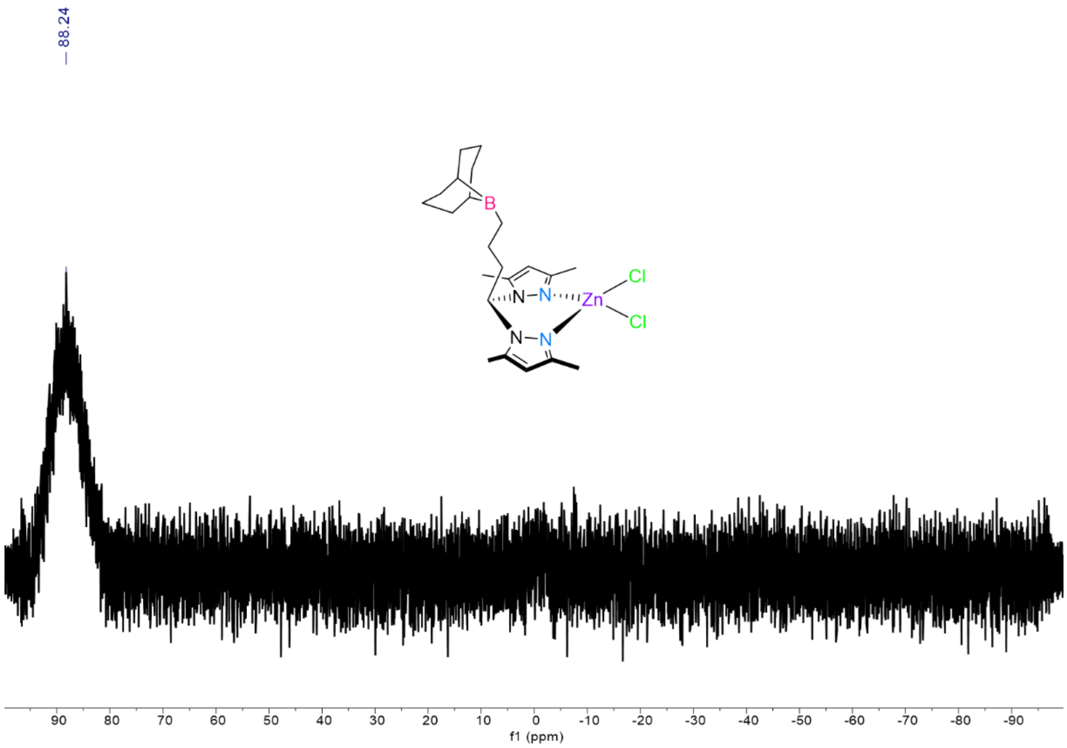

Figure S21 ^{11}B NMR spectrum (96 MHz, $CDCl_3$) of complex **3a**.

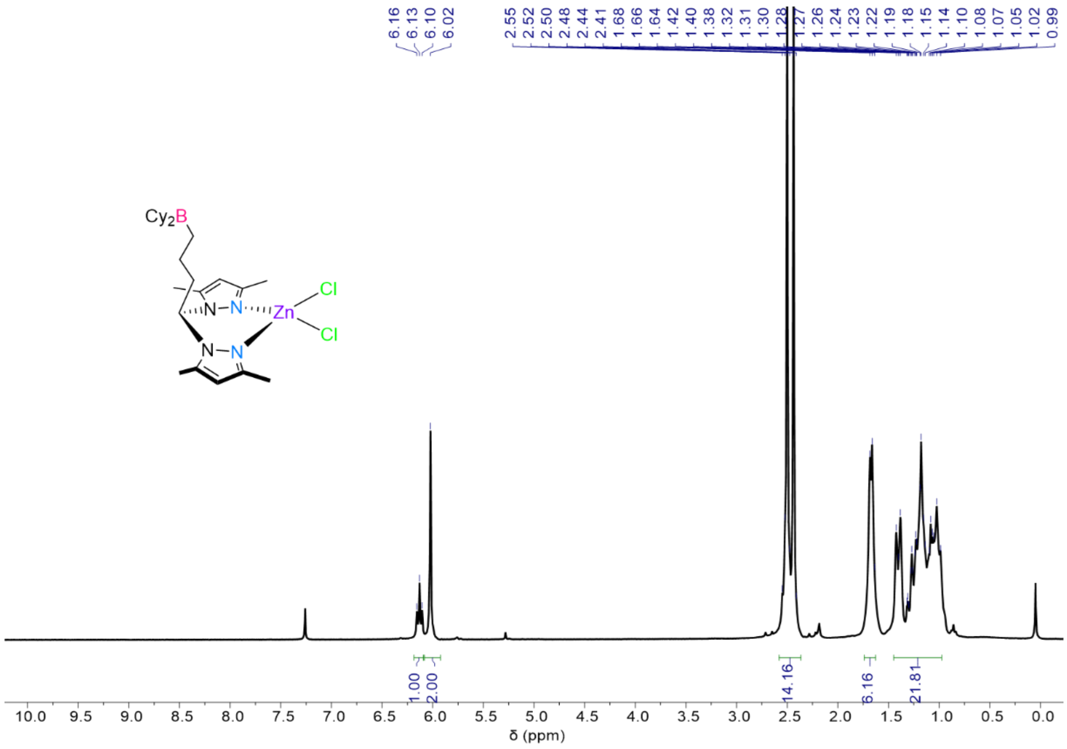

Figure S22 ^{1}H NMR spectrum (300 MHz, $CDCl_3$) of complex **3b**.

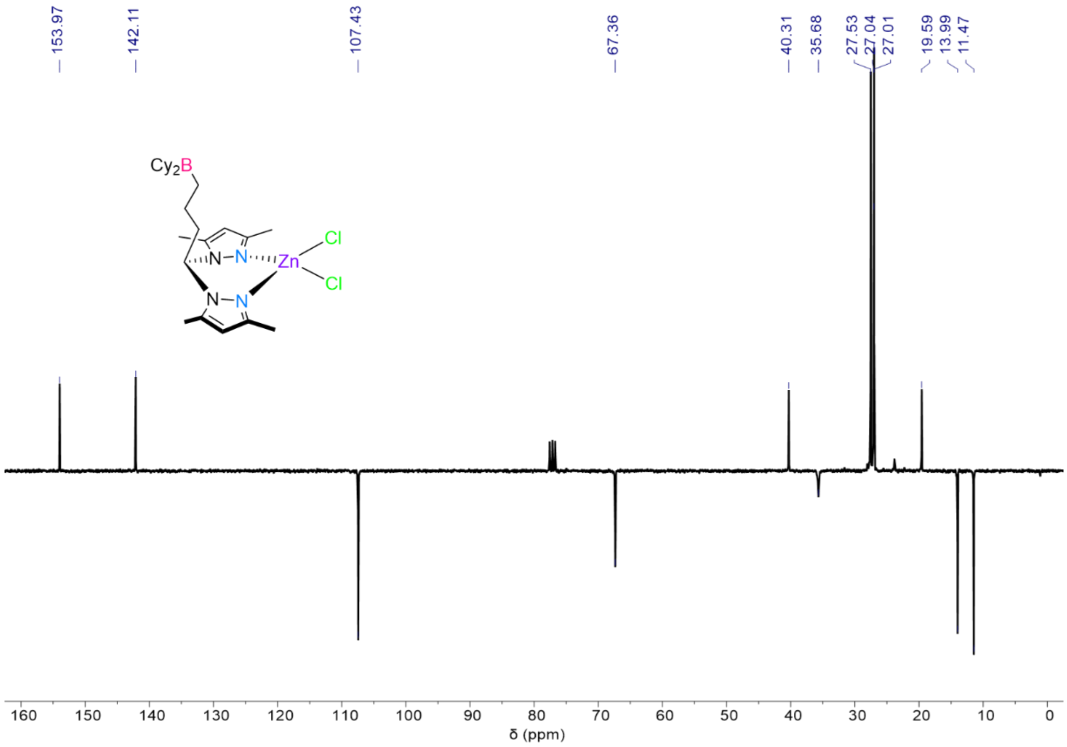

Figure S23 ^{13}C APT NMR spectrum (75 MHz, $CDCl_3$) of complex **3b**.

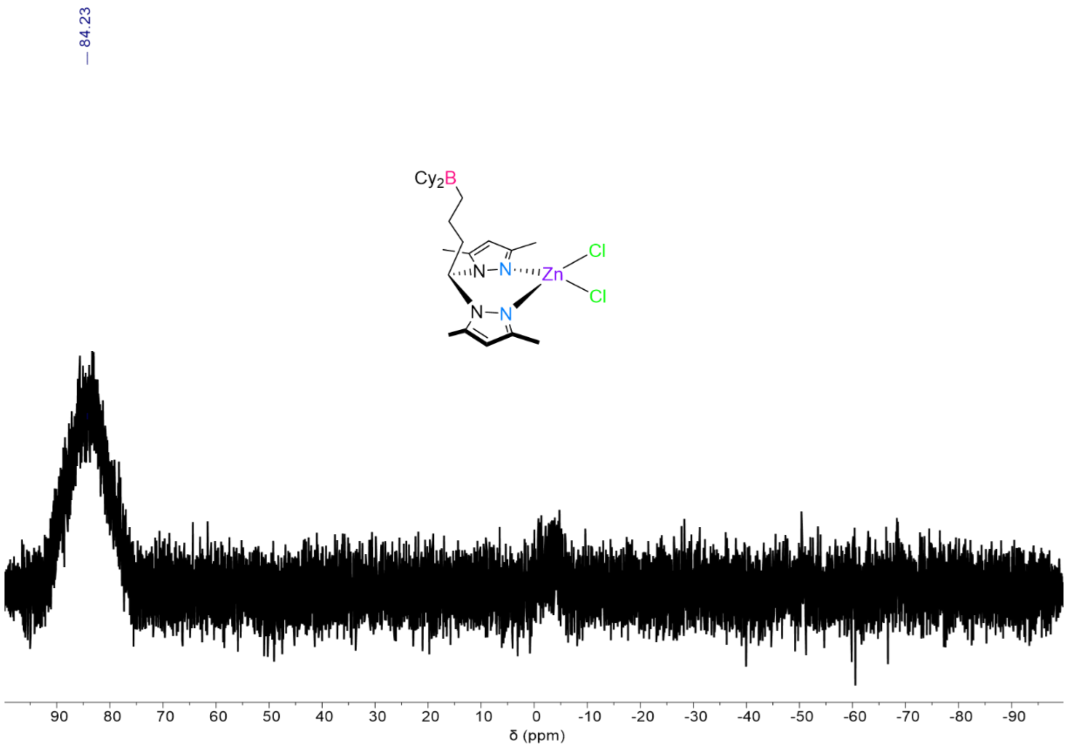

Figure S24 ^{11}B NMR spectrum (96 MHz, $CDCl_3$) of complex **3b**.

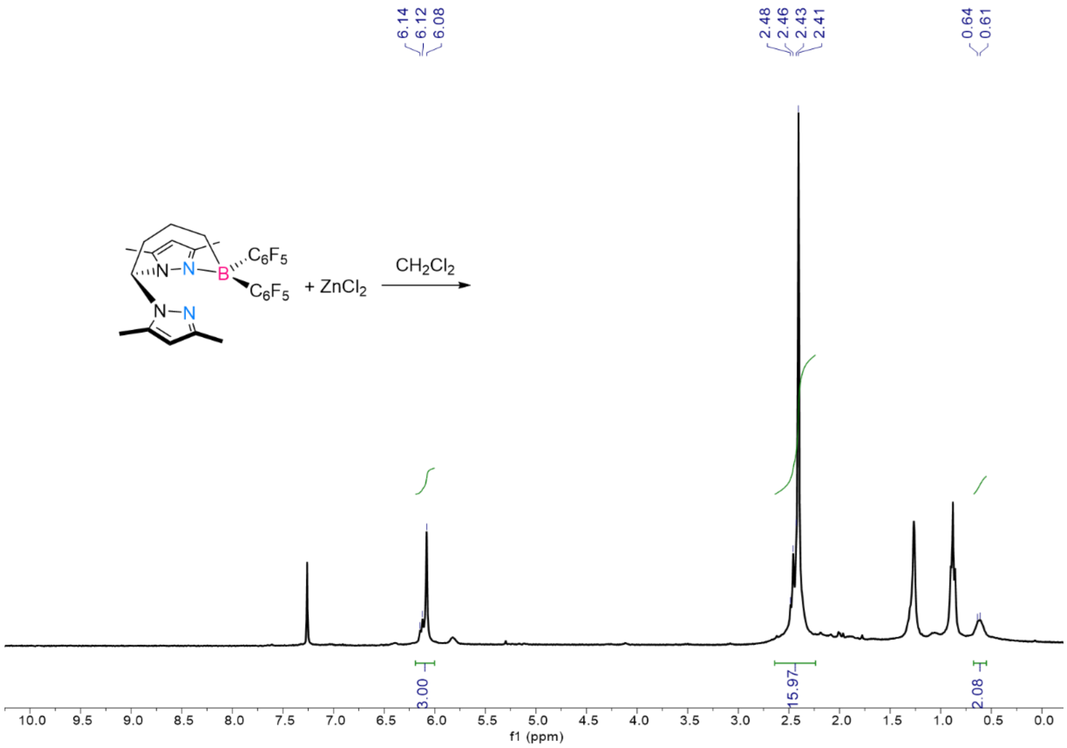

Figure S25 ^{1}H NMR spectrum (300 MHz, $CDCl_3$) of the synthesis attempt of complex **[(1c)ZnCl2]**.

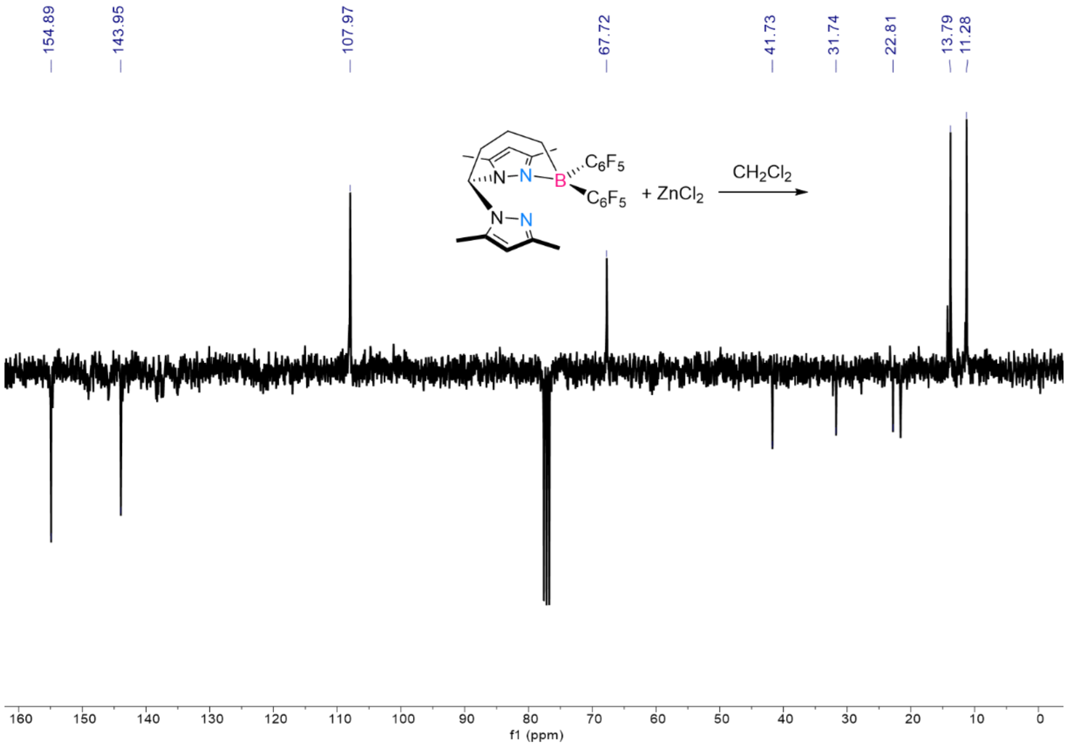

Figure S26 ^{13}C APT NMR spectrum (75 MHz, $CDCl_3$) of the synthesis attempt of complex **[(1c)ZnCl2]**.

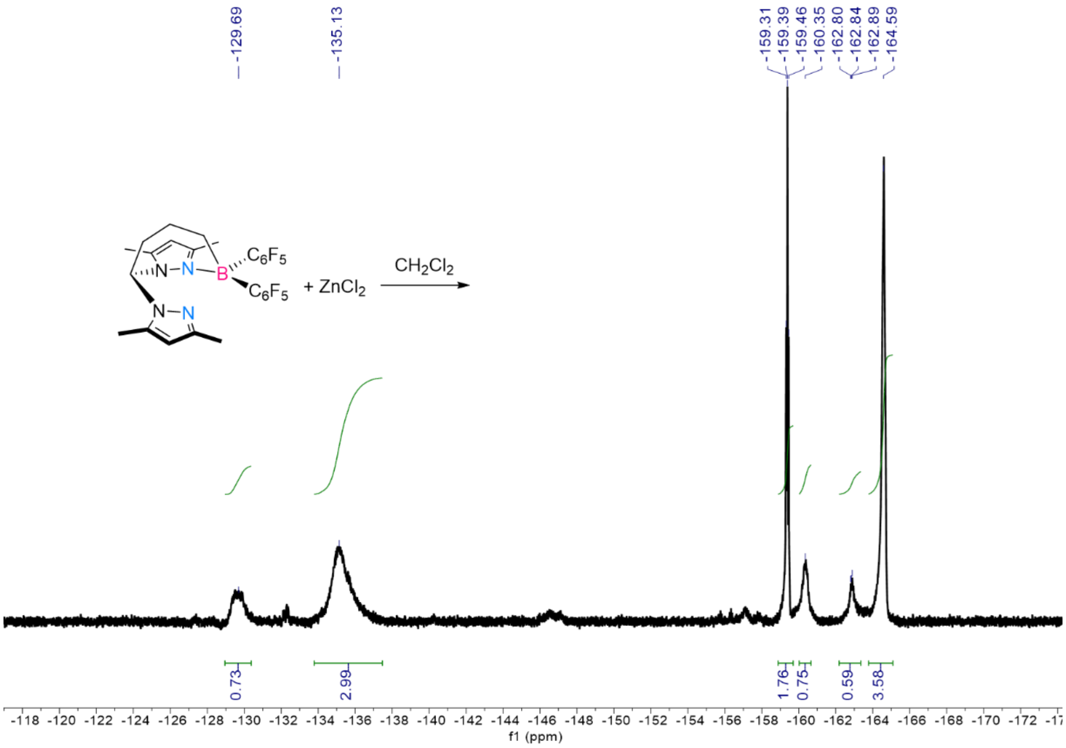

Figure S27 ^{19}F NMR spectrum (96 MHz, $CDCl_3$) of the synthesis attempt of complex **[(1c)ZnCl$_2$]**.

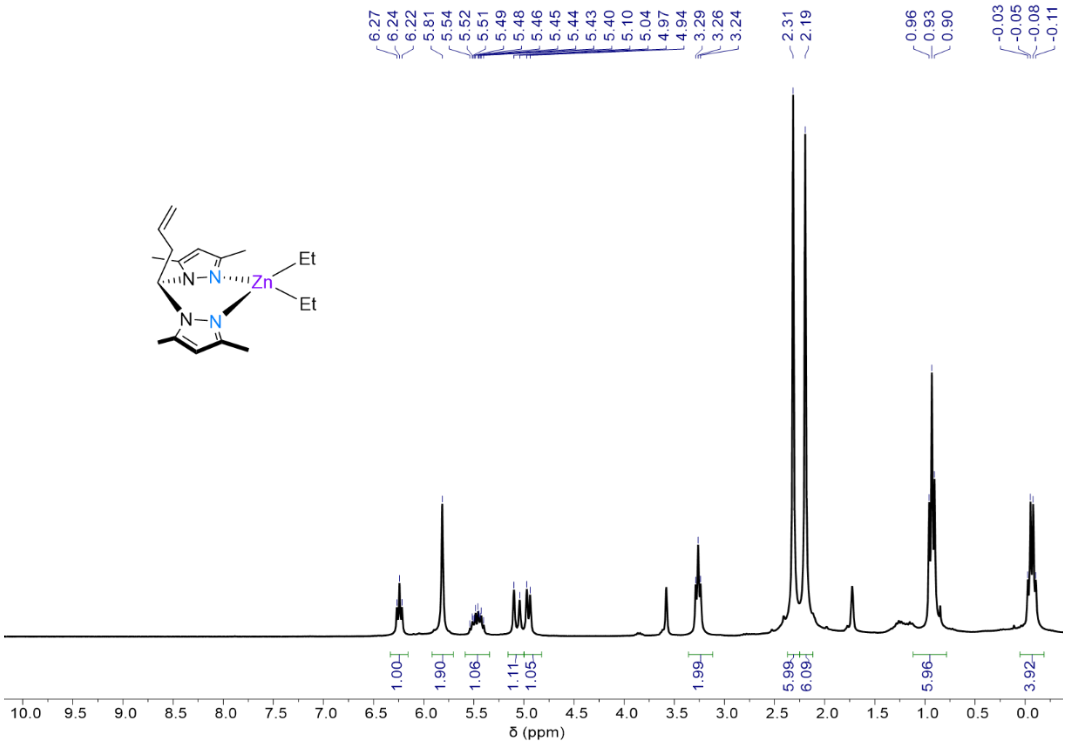

Figure S28 ^{1}H NMR spectrum (300 MHz, THF-d_8) of complex **4**.

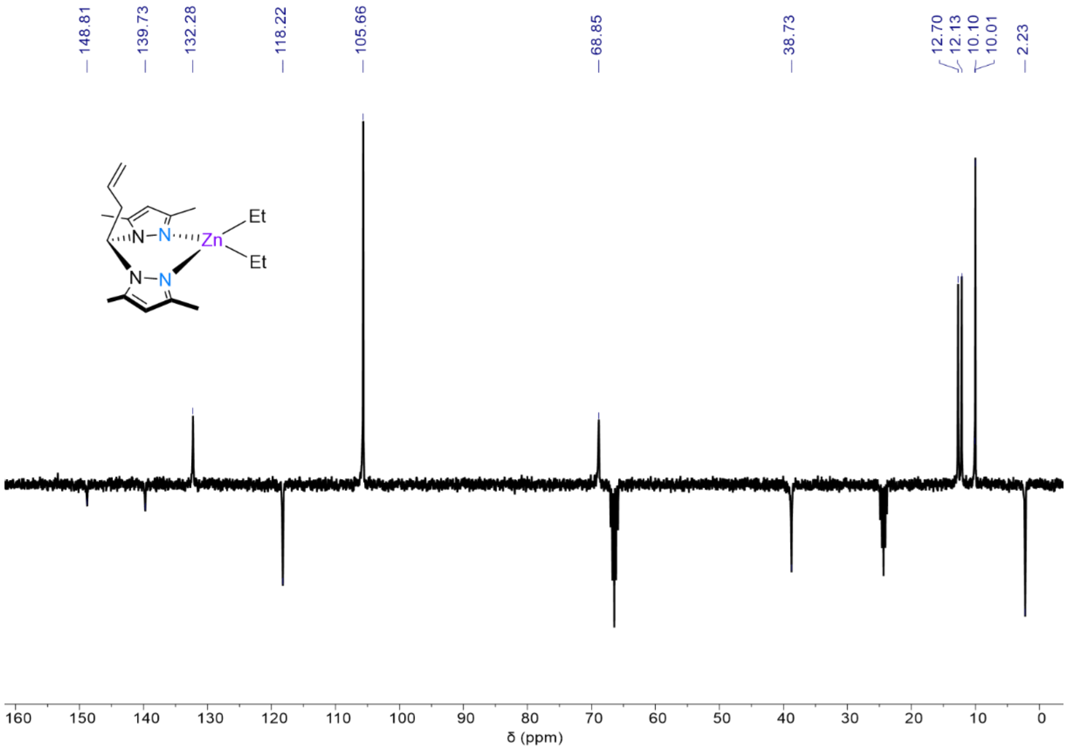

Figure S29 ^{13}C APT NMR spectrum (75 MHz, THF-d_8) of complex **4**.

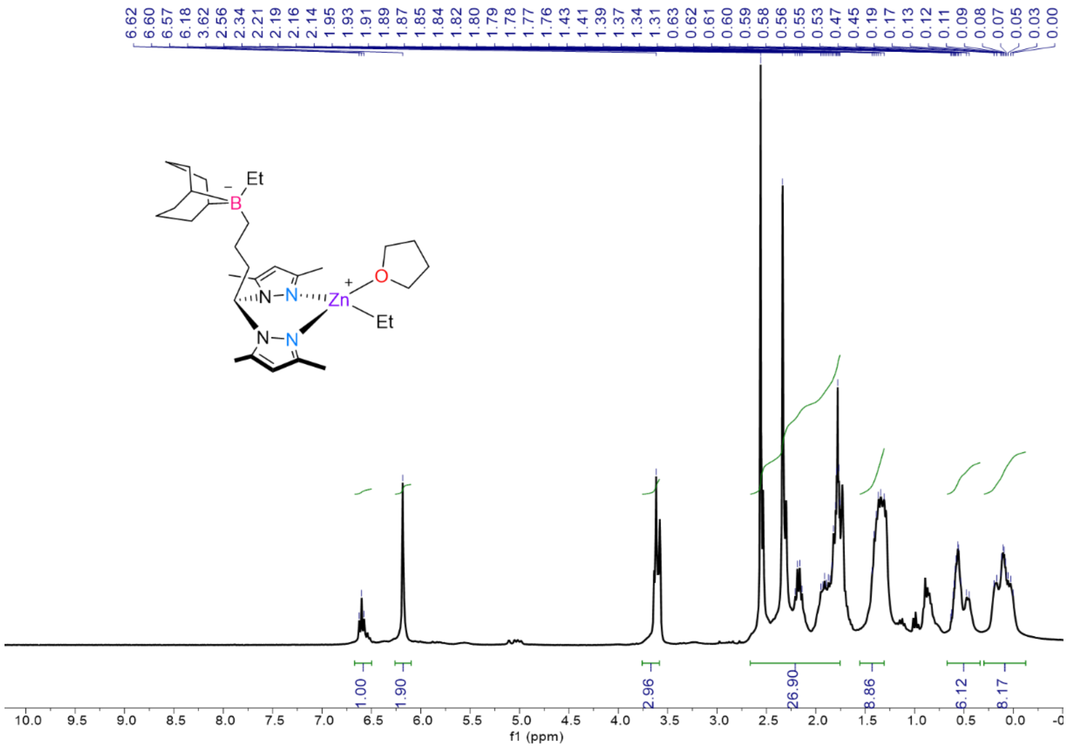

Figure S30 ^{1}H NMR spectrum (300 MHz, THF-d_8) of complex **5**.

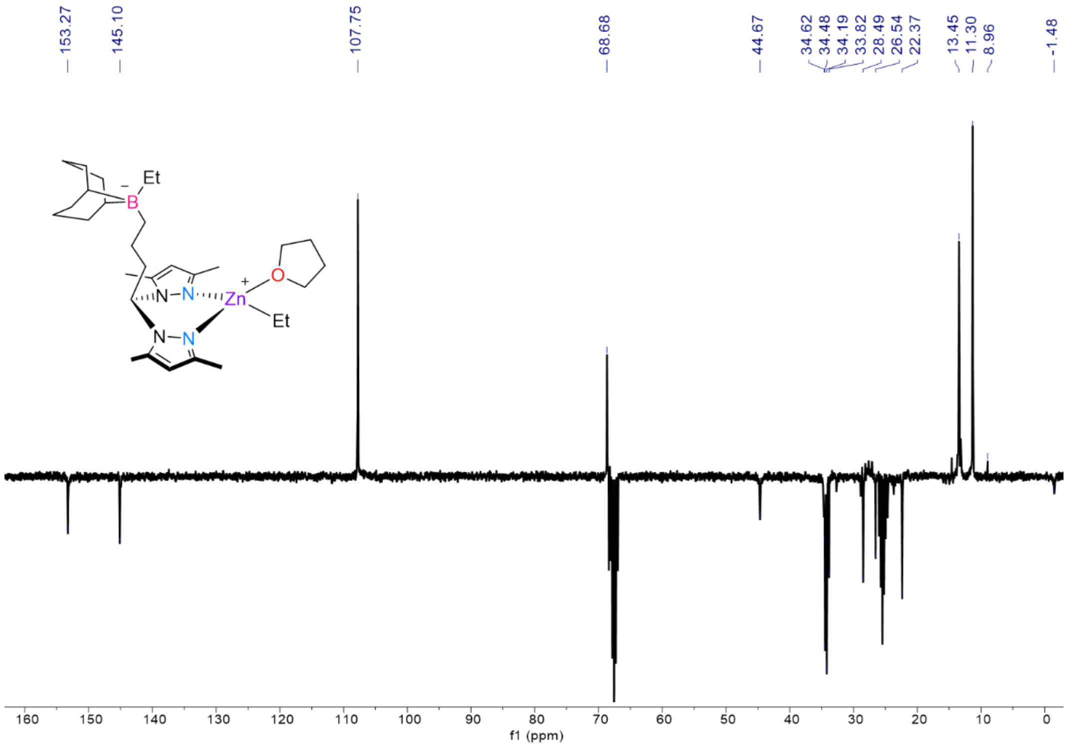

Figure S31 ^{13}C APT NMR spectrum (75 MHz, THF-d_8) of complex **5**.

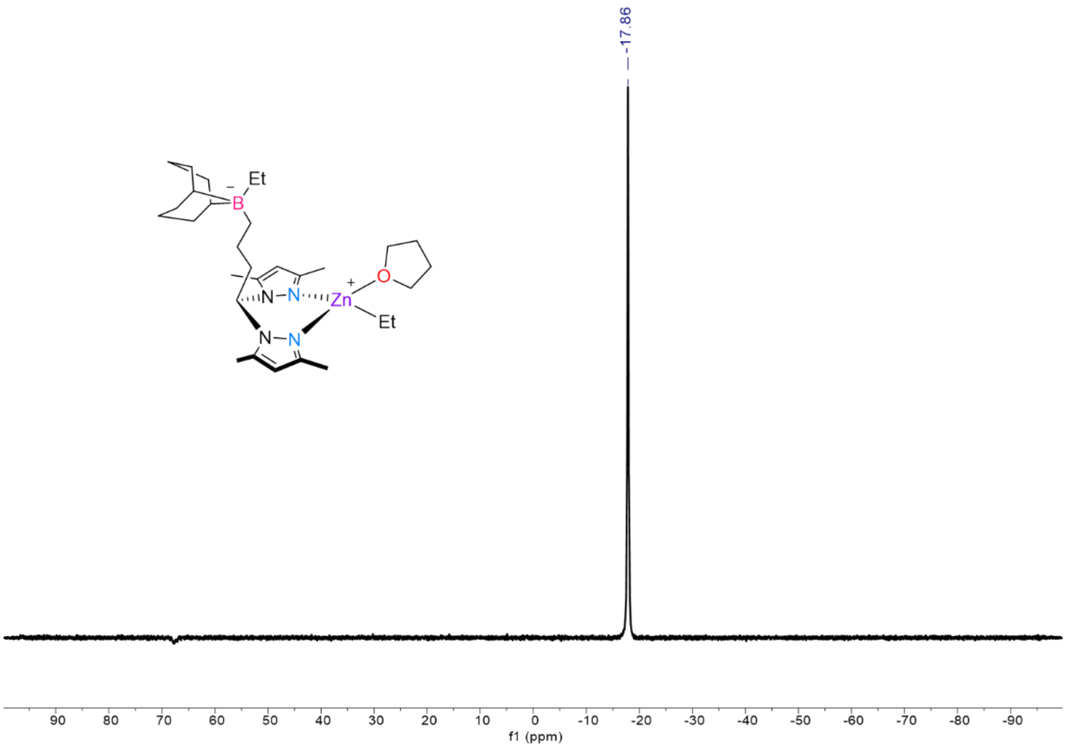

Figure S32 ^{11}B NMR spectrum (96 MHz, THF-d_8) of complex **5**.

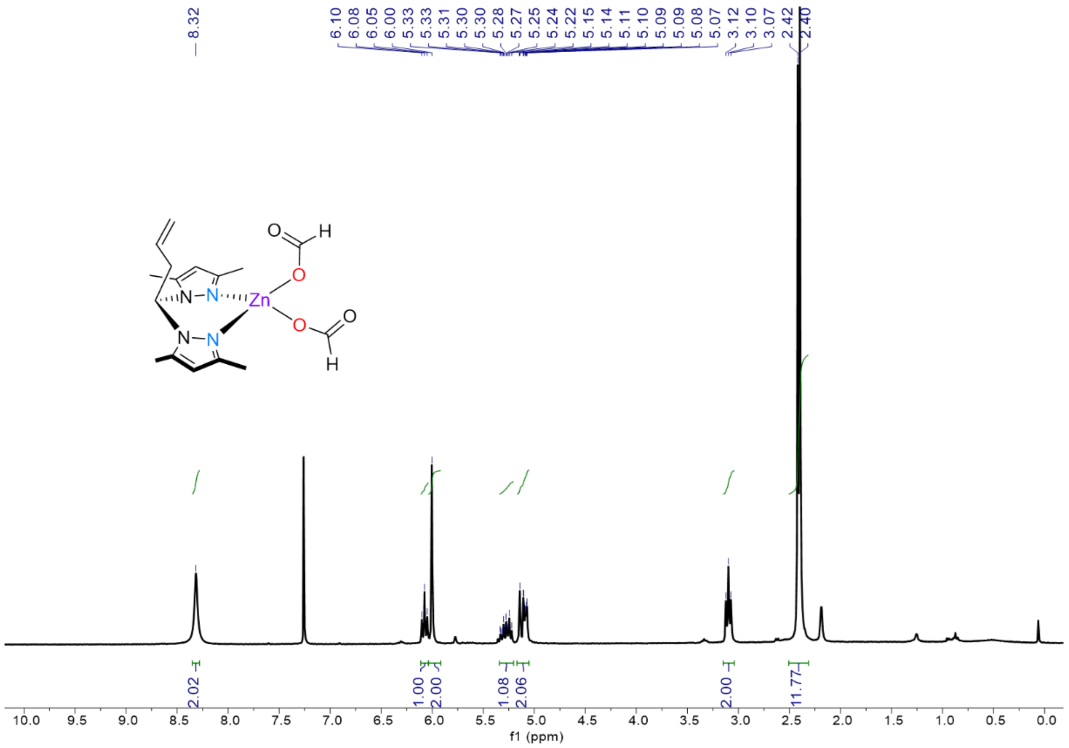

Figure S33 ^{1}H NMR spectrum (300 MHz, $CDCl_3$) of complex **7**.

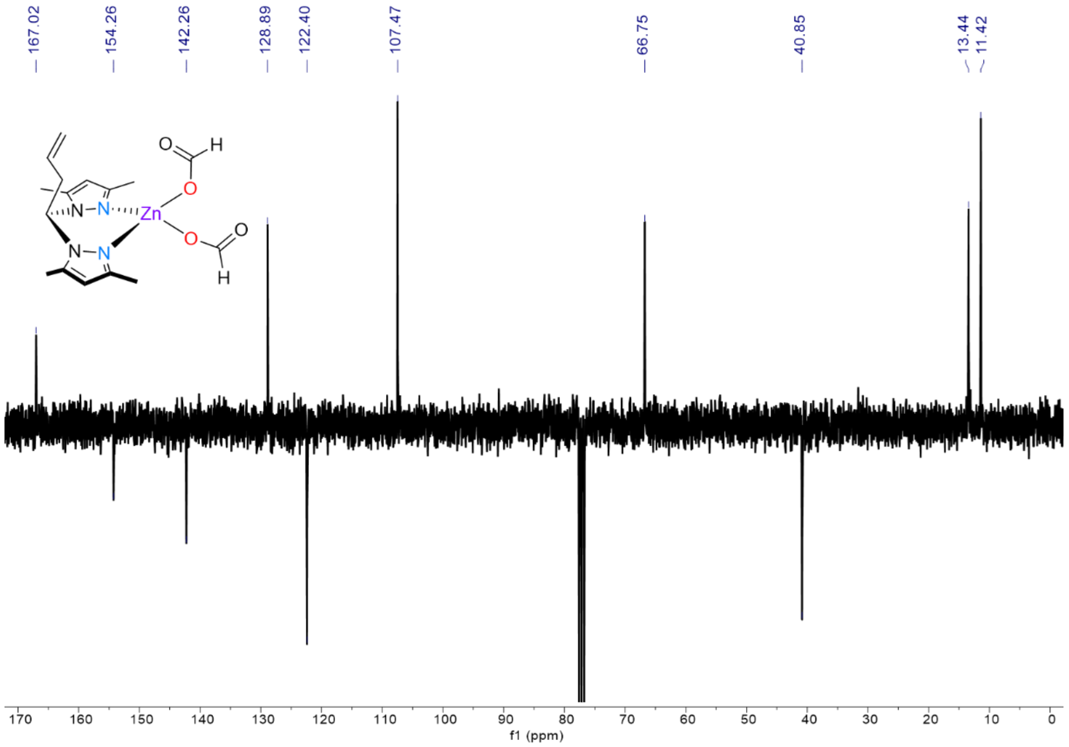

Figure S34 ^{13}C APT NMR spectrum (300 MHz, $CDCl_3$) of complex **7**.

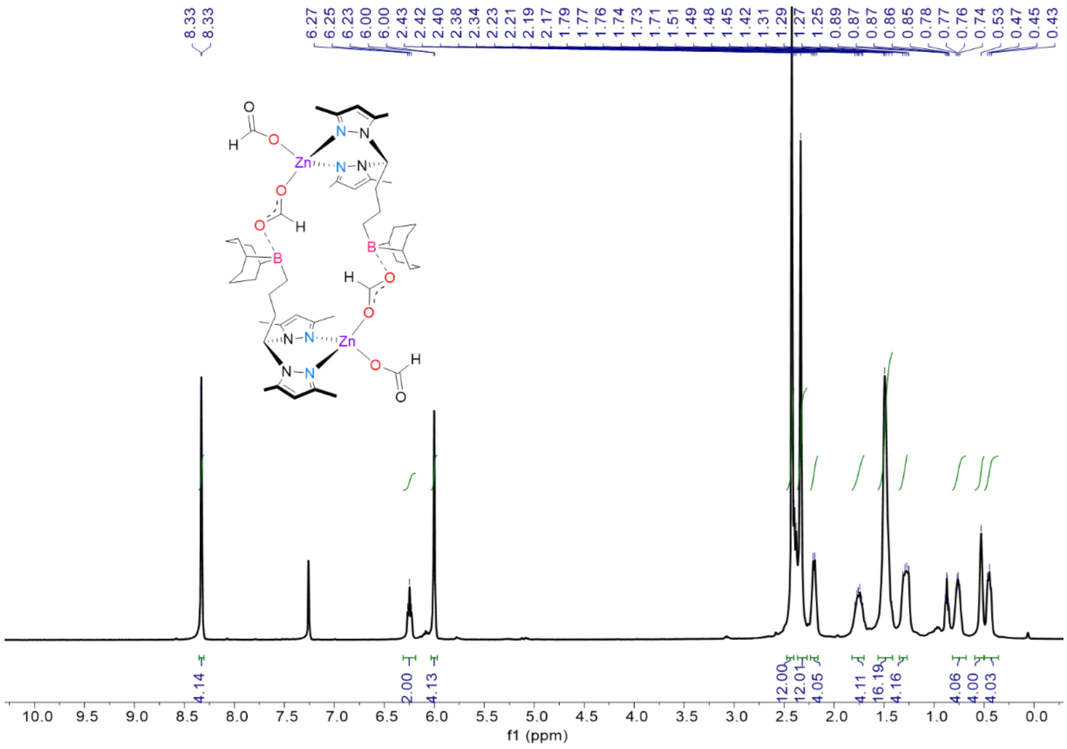

Figure S35 ^{1}H NMR spectrum (300 MHz, $CDCl_3$) of complex **8**.

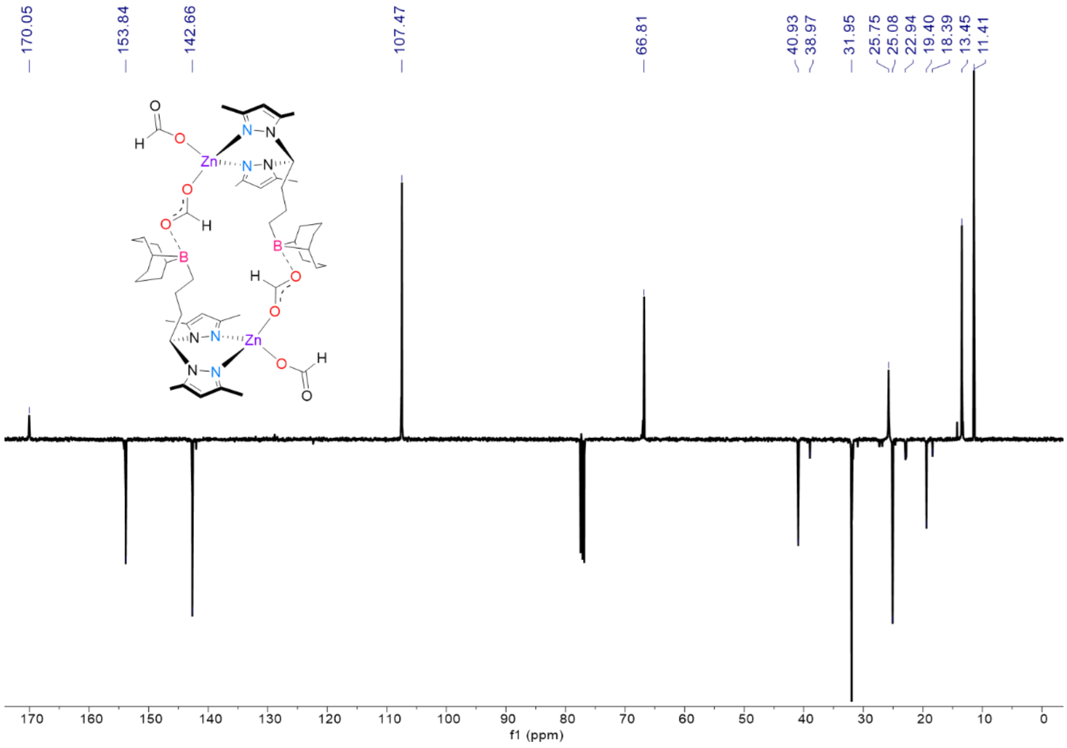

Figure S36 ^{13}C APT NMR spectrum (75 MHz, $CDCl_3$) of complex **8**.

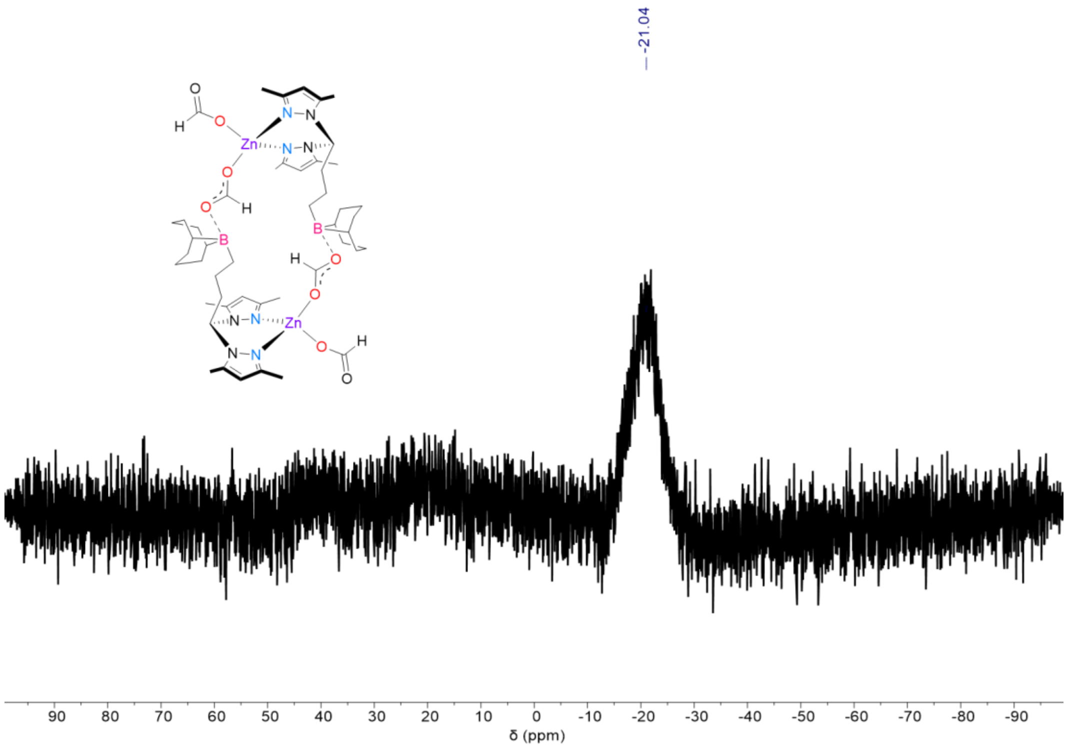

Figure S37 ^{11}B NMR spectrum (96 MHz, $CDCl_3$) of complex **8**.

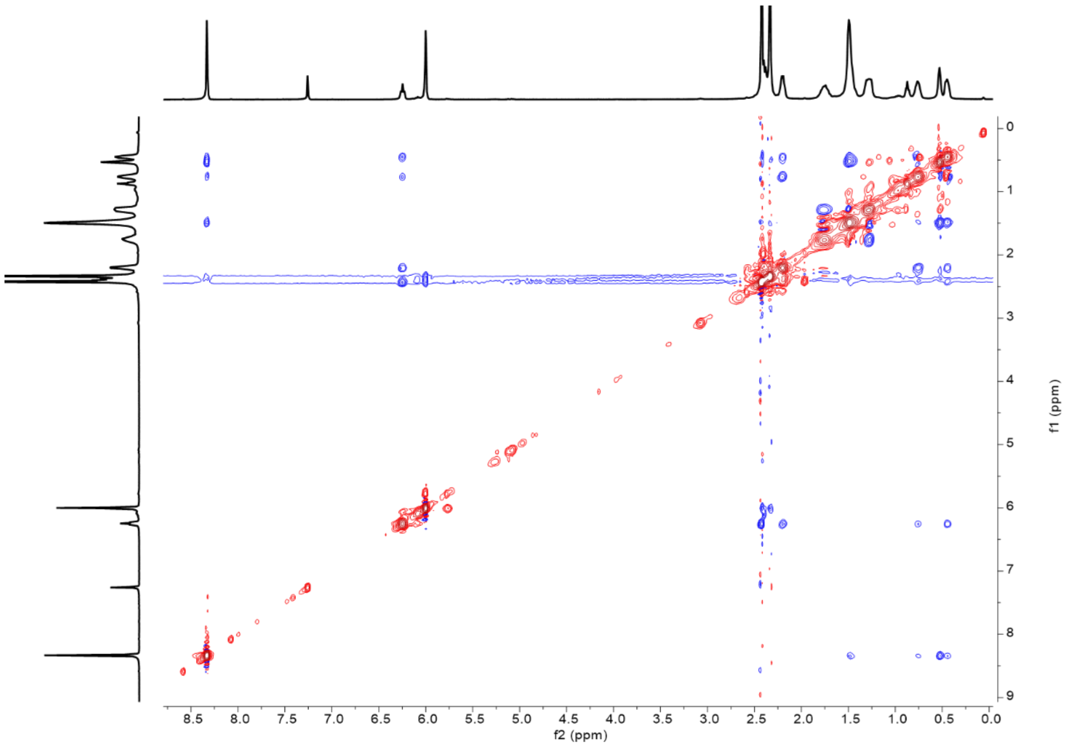

Figure S38 ^{1}H-^{1}H NOESY NMR spectrum (300 MHz, $CDCl_3$) of complex **8**.

DOSY NMR data for complexes 7 and 8

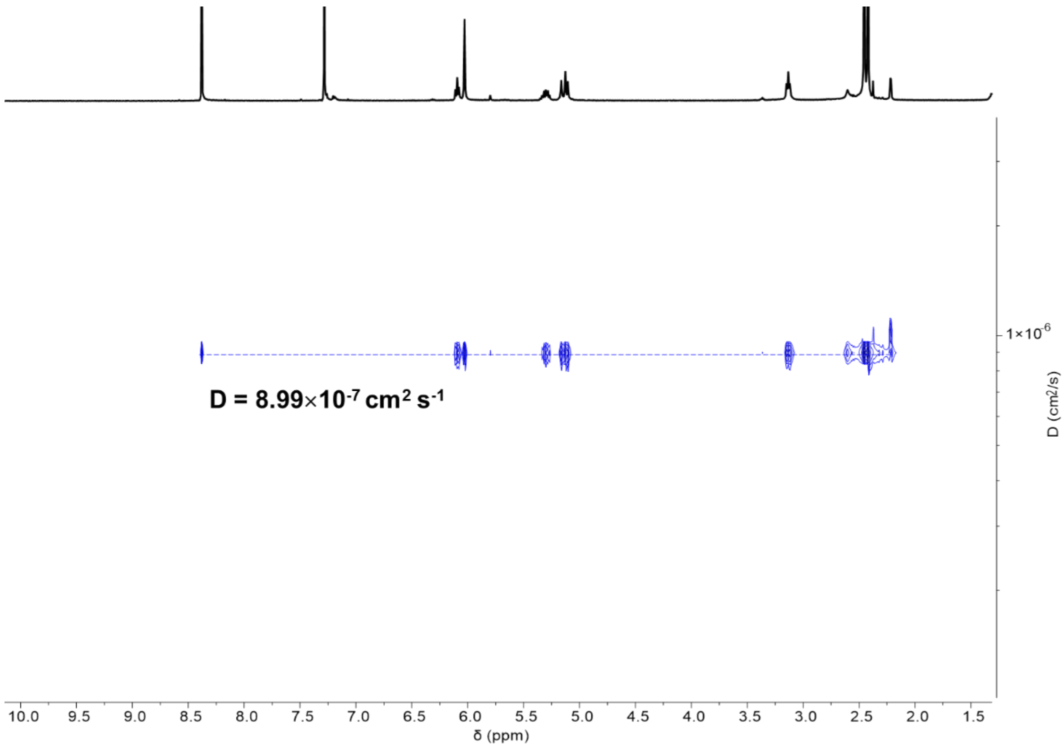

Figure S39 DOSY NMR spectrum (300 MHz, $CDCl_3$) of complex **7** and its average diffusion coefficient. The blue dashed line is a guide to the eye.

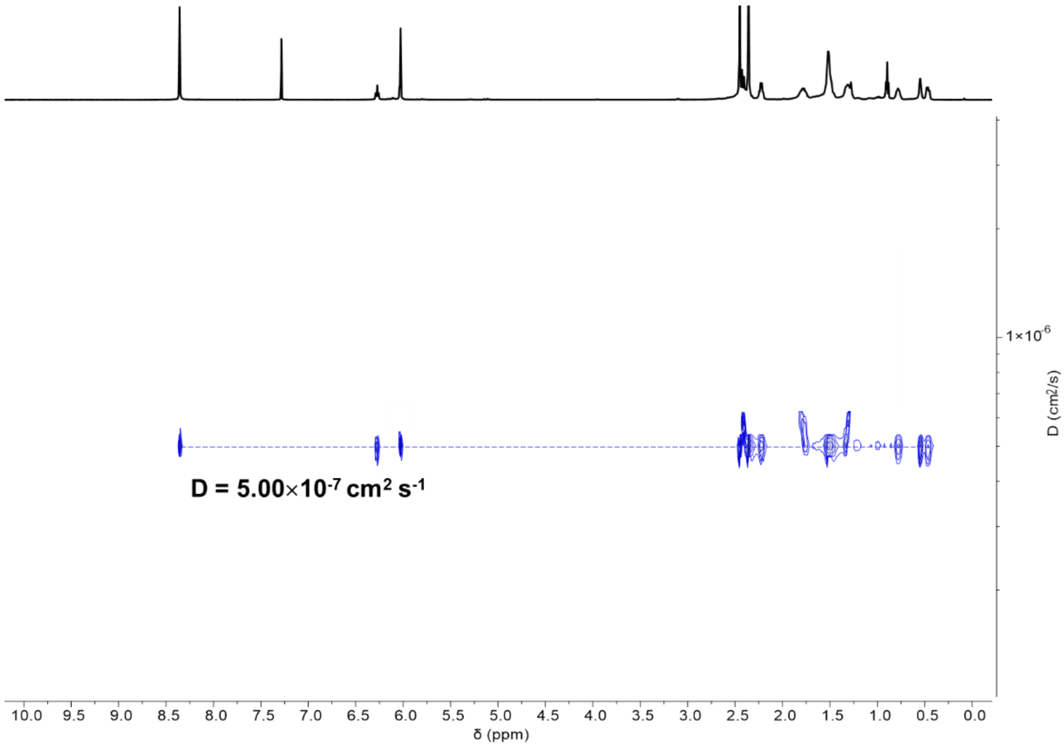

Figure S40 DOSY NMR spectrum (300 MHz, $CDCl_3$) of complex **8** and its average diffusion coefficient. The blue dashed line is a guide to the eye.

FTIR spectra of the complexes

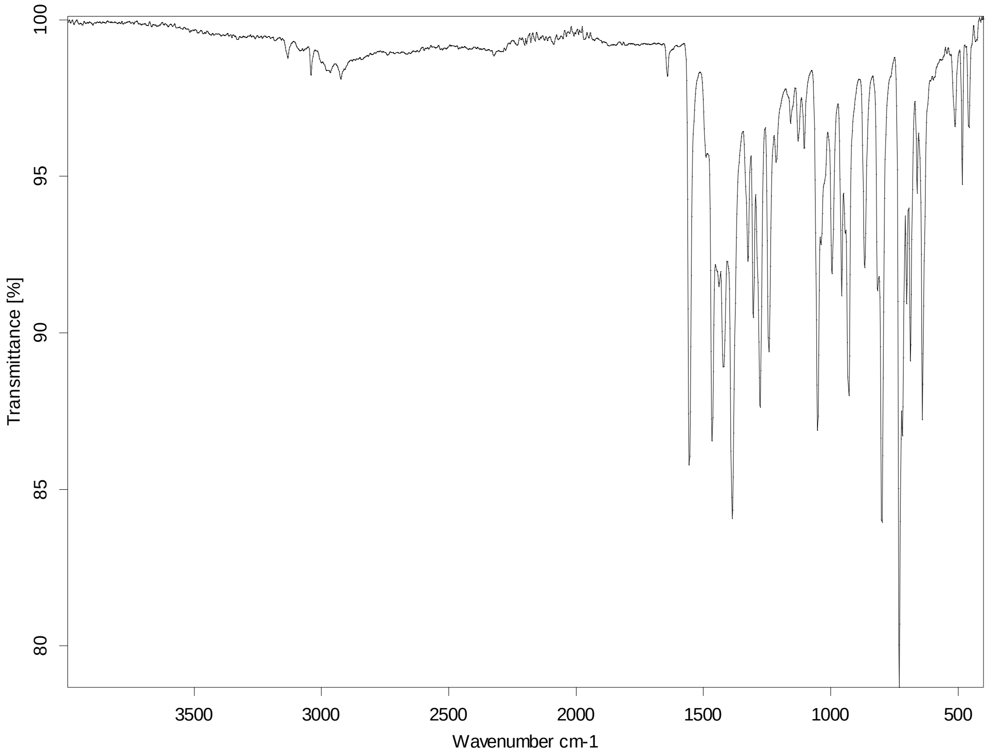

Figure S41 ATR-FTIR spectrum of complex **2**.

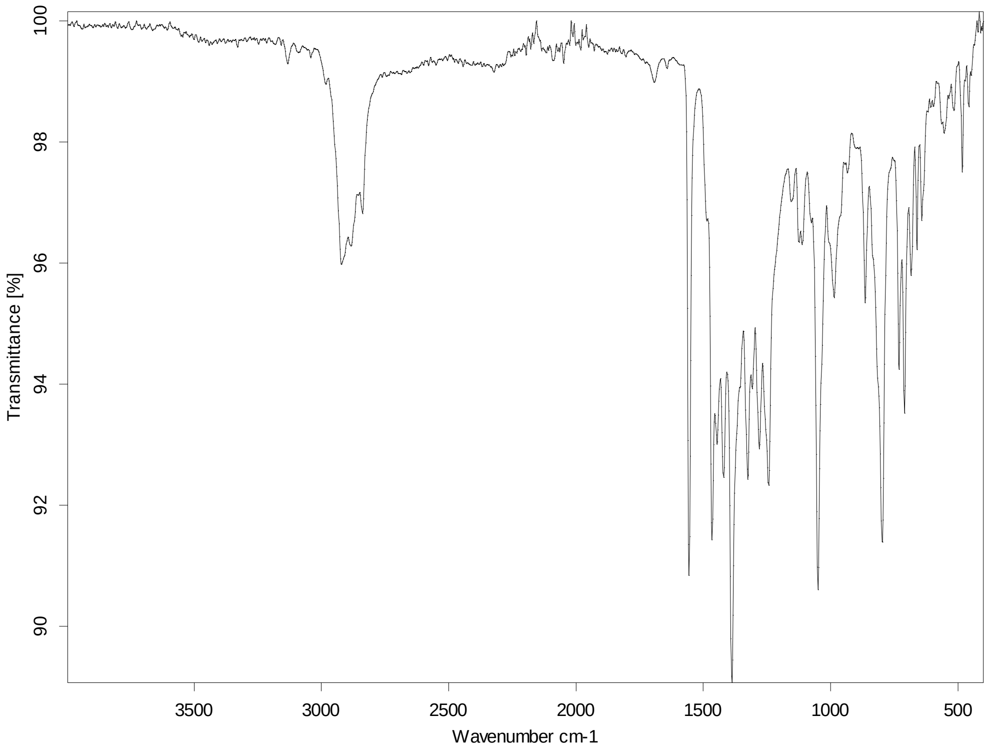

Figure S42 ATR-FTIR spectrum of complex **3a**.

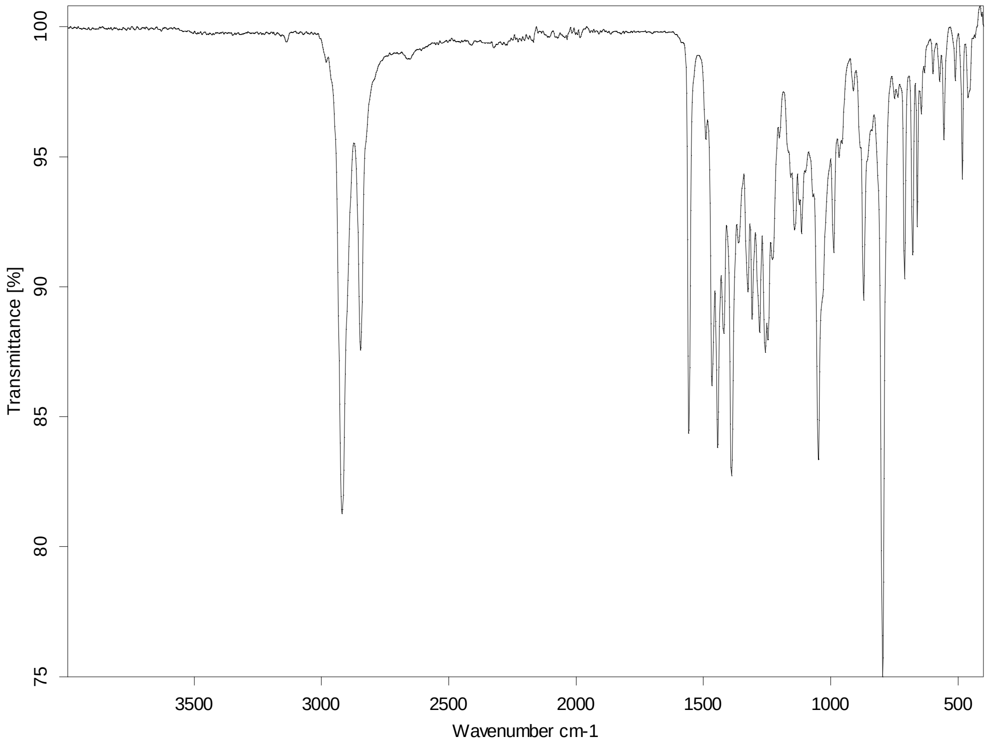

Figure S43 ATR-FTIR spectrum of complex **3b**.

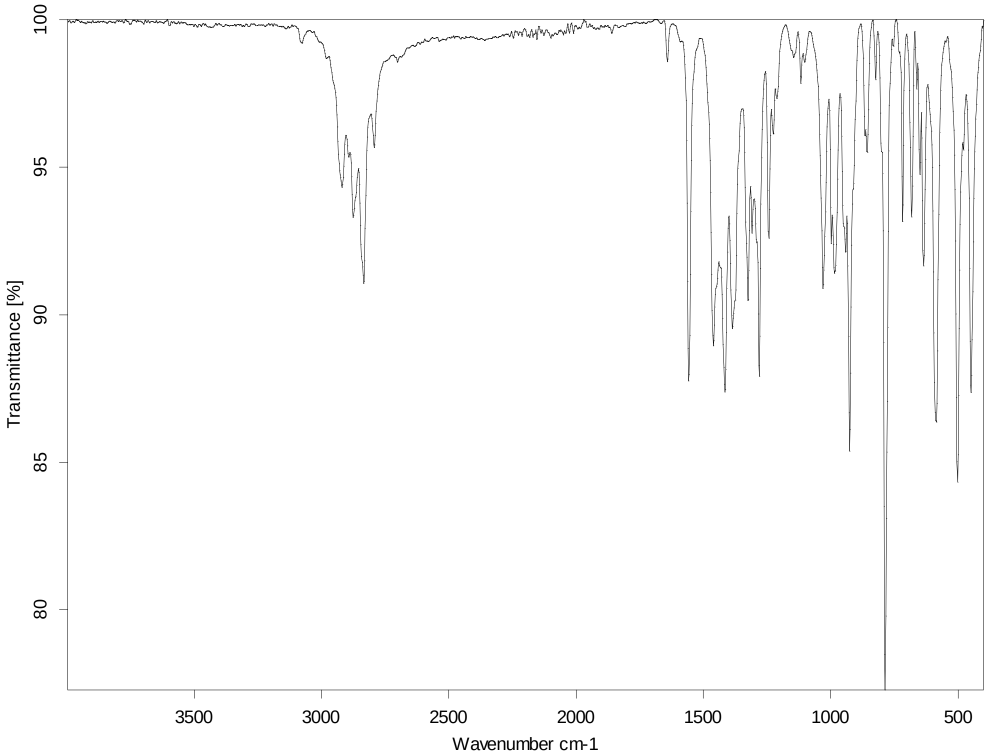

Figure S44 ATR-FTIR spectrum of complex **4**.

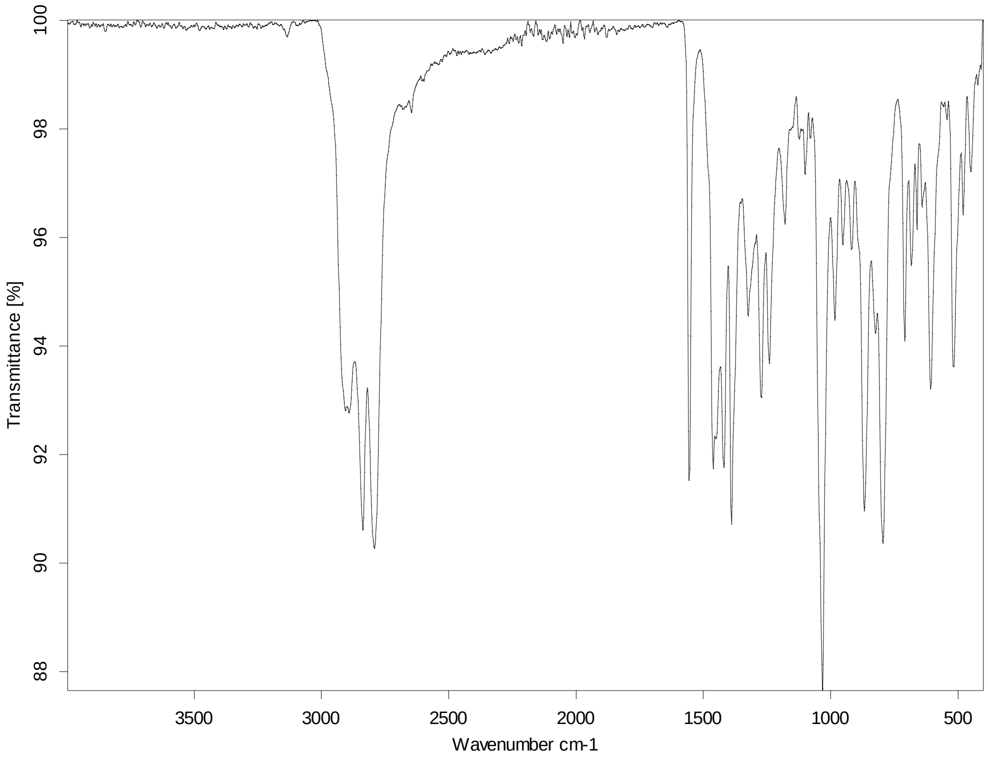

Figure S45 ATR-FTIR spectrum of complex **5**.

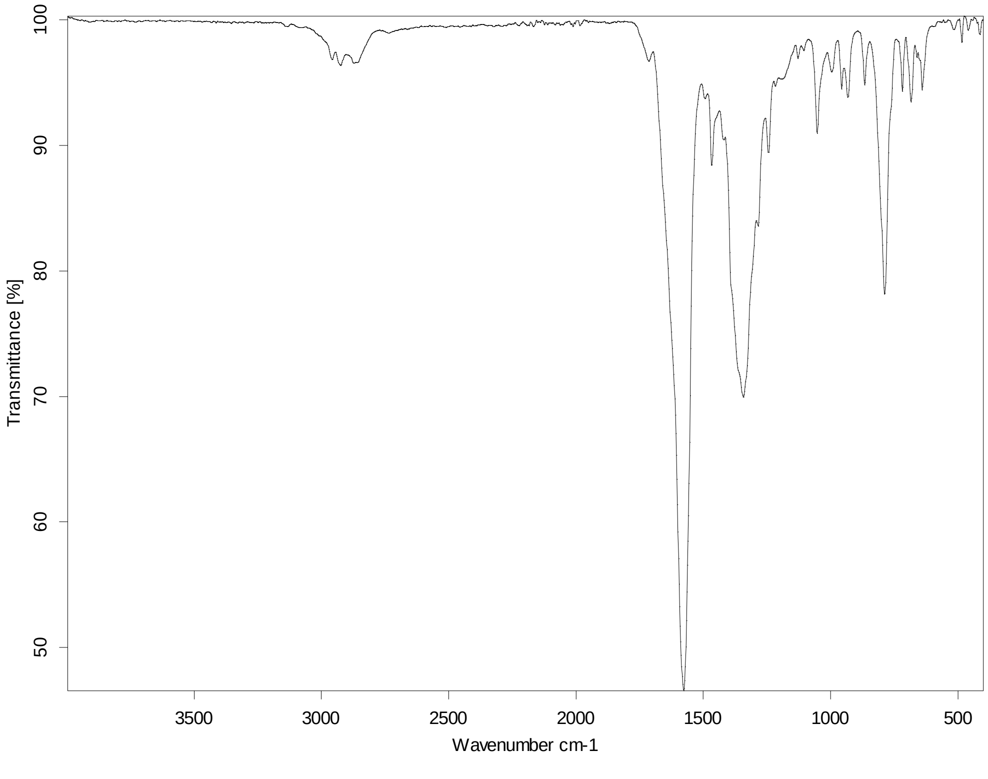

Figure S46 ATR-FTIR spectrum of complex **7**.

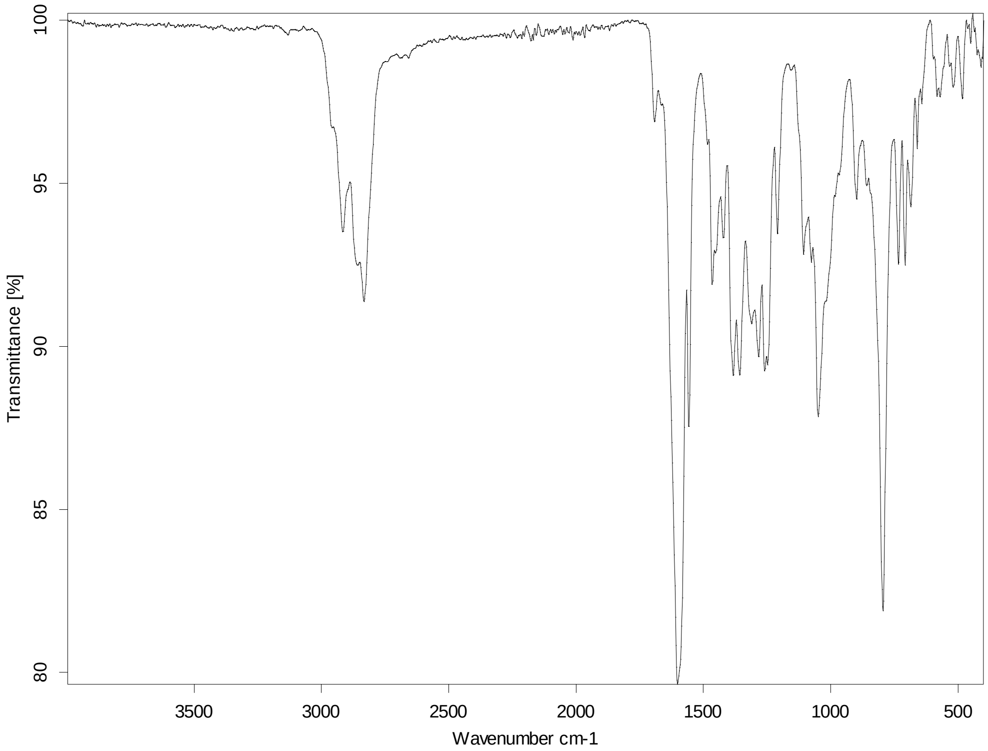

Figure S47 ATR-FTIR spectrum of complex **8**.

Supplementary single crystal X-ray diffraction data

Table S1 Selected bond distances (Å) and bond angles (º) for **1a**, **2**, **3a** and **7**. X indicates either Cl or O atoms, when applicable.

	**1a**	**2**	**3a**	**7**
Lengths (Å)				
Zn1–N1	-	2.036(3)	2.035(16)	2.071(3)
Zn1–N3	-	2.027(3)	2.06(2)	2.027(3)
Zn1–X1	-	2.2336(10)	2.218(6)	1.967(3)
Zn1–X2	-	2.2251(12)	2.192(5)	1.949(3)
N2–C3	1.354(5)	1.357(4)	1.38(3)	1.369(5)
N2–N1	1.379(4)	1.375(4)	1.33(2)	1.361(4)
N2–C11	1.470(4)	1.470(4)	1.428(19)	1.460(5)
N1–C1	1.325(5)	1.340(5)	1.41(3)	1.340(5)
C3–C2	1.378(5)	1.381(5)	1.31(3)	1.377(6)
C11–C12	1.513(5)	1.527(6)	1.389(19)	1.537(5)
C12–C13	1.532(5)	1.493(6)	1.44(2)	1.502(6)
C14–C13	1.512(5)	1.299(6)	1.48(2)	1.308(6)
B1–C14	1.561(6)	-	1.56(2)	-
B1–C15	1.542(7)	-	1.58(2)	-
B1–C16	1.557(6)	-	1.60(2)	-
Angles (º)				
N3–Zn1–N1	-	93.04(12)	93.0(4)	92.64(13)
N1–Zn1–X1	-	107.91(9)	111.7(5)	99.57(13)
N1–Zn1–X2	-	114.07(10)	112.4(5)	106.85(13)
X2–Zn1–X1	-	116.26(4)	115.9(2)	118.32(14)
C3–N2–N1	112.7(3)	111.4(3)	110(2)	111.3(3)
C1–N1–N2	104.3(3)	105.4(3)	107.2(16)	106.1(3)
N2–C3–C2	105.9(3)	106.0(3)	103.1(15)	105.5(4)
C13–C14–B1	121.0(3)	-	127(2)	-
C11–C12–C13	113.9(3)	110.7(3)	105(2)	112.5(3)
C14–C13–C12	110.1(3)	124.5(4)	102.8(16)	124.3(5)
C15–B1–C14	127.8(4)	-	121(2)	-
C15–B1–C16	111.0(4)	-	108(2)	-
C14–B1–C16	121.1(4)	-	131(2)	-

Table S2 Crystallographic data for **1a**, **2**, **3a** and **7**.

	**1a**	**2**	**3a**	**7**
Formula	$C_{22}H_{35}BN_4$	$C_{15}H_{22}Cl_4N_4Zn$	$C_{22}H_{35}BCl_2N_4Zn$	$C_{16}H_{22}N_4O_4Zn$
M	366.35	465.53	502.62	399.74
λ (Å)	0.71073	0.71073	0.71073	0.71073
T (K)	150	150	150	150
Crystal system	Triclinic	Monoclinic	Monoclinic	Monoclinic
Space group	P-1	$P2_1/n$	$P2_1$	$P2_1/n$
a (Å)	9.060(3)	8.5195(6)	8.5722(9)	11.849(4)
b (Å)	9.339(4)	13.3379(12)	13.8455(13)	8.603(3)
c (Å)	13.907(5)	18.4519(18)	11.3802(13)	18.471(6)
α (Å)	70.962(14)	90	90	90
β (Å)	75.946(13)	100.887(3)	96.369(4)	101.045(13)
γ (Å)	85.744(14)	90	90	90
V ($Å^3$)	1079.0(7)	2059.0(3)	1342.3(2)	1848.0(11)
Z	2	4	2	4
ρ_{calc} (g.cm^{-3})	1.128	1.502	1.244	1.437
μ (mm^{-1})	0.067	1.717	1.129	1.356
Crystal size (mm)	0.30×0.25×0.25	0.25×0.15×0.05	0.20×0.07×0.07	0.10×0.05×0.05
θ_{max} (º)	26.729	26.390	26.756	26.671
Total data	7353	9203	10566	12632
Unique data	4465	4172	4335	3874
R_{int}	0.0386	0.0681	0.0478	0.0892
R [$I > 2\sigma(I)$]	0.1022	0.0491	0.1055	0.0533
R_w	0.1557	0.0899	0.1568	0.1216
Goodness of fit	1.074	0.949	1.125	0.997
ρ_{min}	-0.466	-0.492	-1.558	-0.545
ρ_{max}	1.113	0.557	1.928	0.453

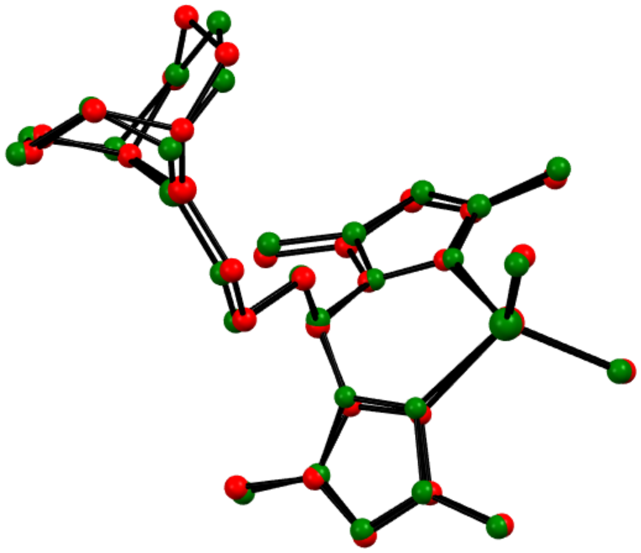

Figure S48 Superimposition of the experimental (determined by single crystal X-ray diffraction, in green) and theoretical (determined by DFT calculations, in red) structures of complex **3a**.

NMR data of the reaction of complex 8 with HBPin

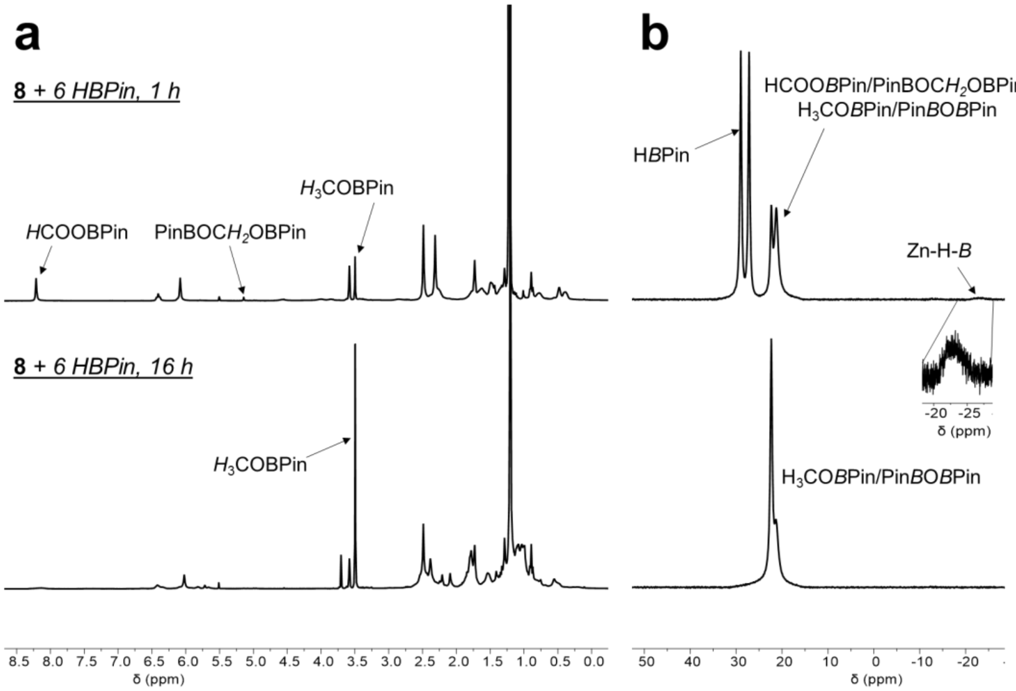

Figure S49 Stacking of the (a) ^{1}H NMR (300 MHz, THF-d_8) and (b) ^{11}B NMR (96 MHz, THF-d_8) spectra of the reaction of complex **8** with 6 equivalents of HBPin for 1 h (top) and 16 h (bottom).

Selected NMR data of the catalytic reactions

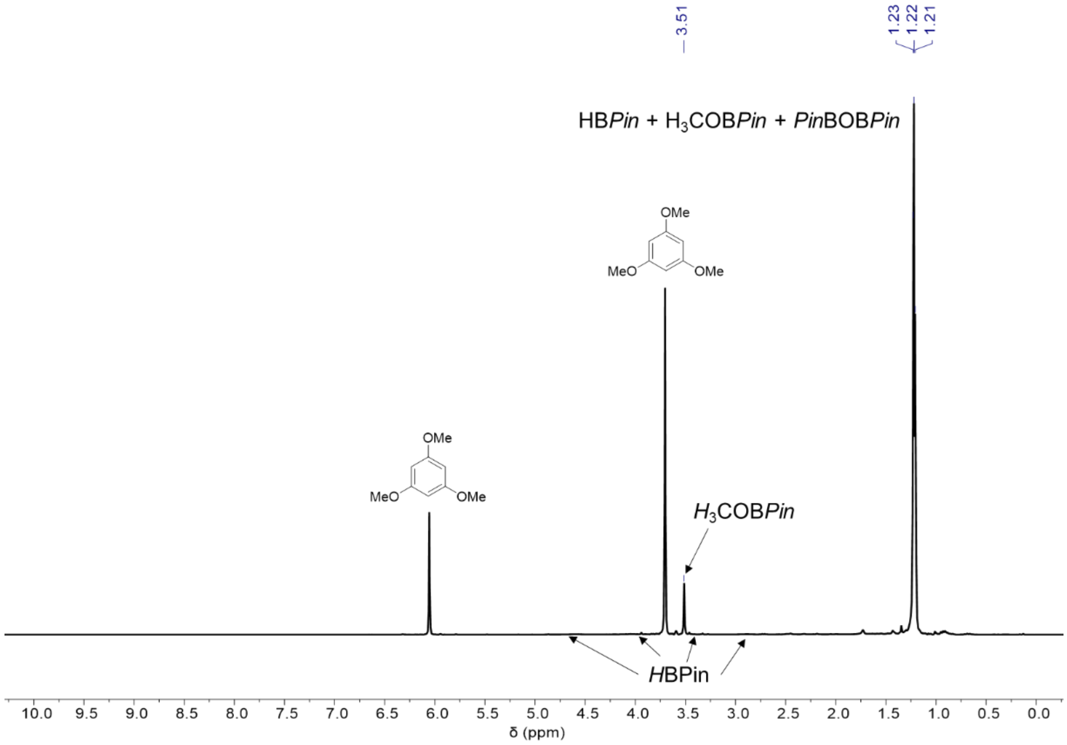

Figure S50 ^{1}H NMR spectrum (300 MHz, THF-d_8) of the hydroboration of CO_2 (1 bar) with HBPin catalyzed by 1 mol% of complex **3b**/2 $KHBEt_3$, at 40 ºC, for 16 h.

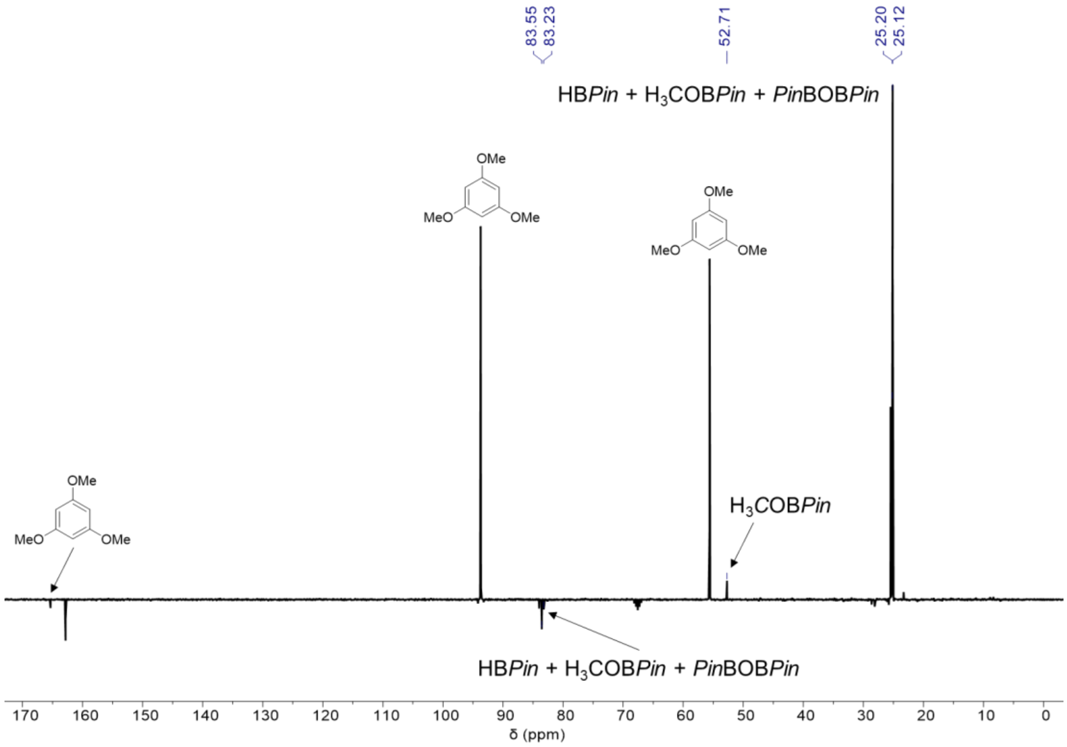

Figure S51 ^{13}C APT NMR spectrum (75 MHz, THF-d_8) of the hydroboration of CO_2 (1 bar) with HBPin catalyzed by 1 mol% of complex **3b**/2 $KHBEt_3$, at 40 ºC, for 16 h.

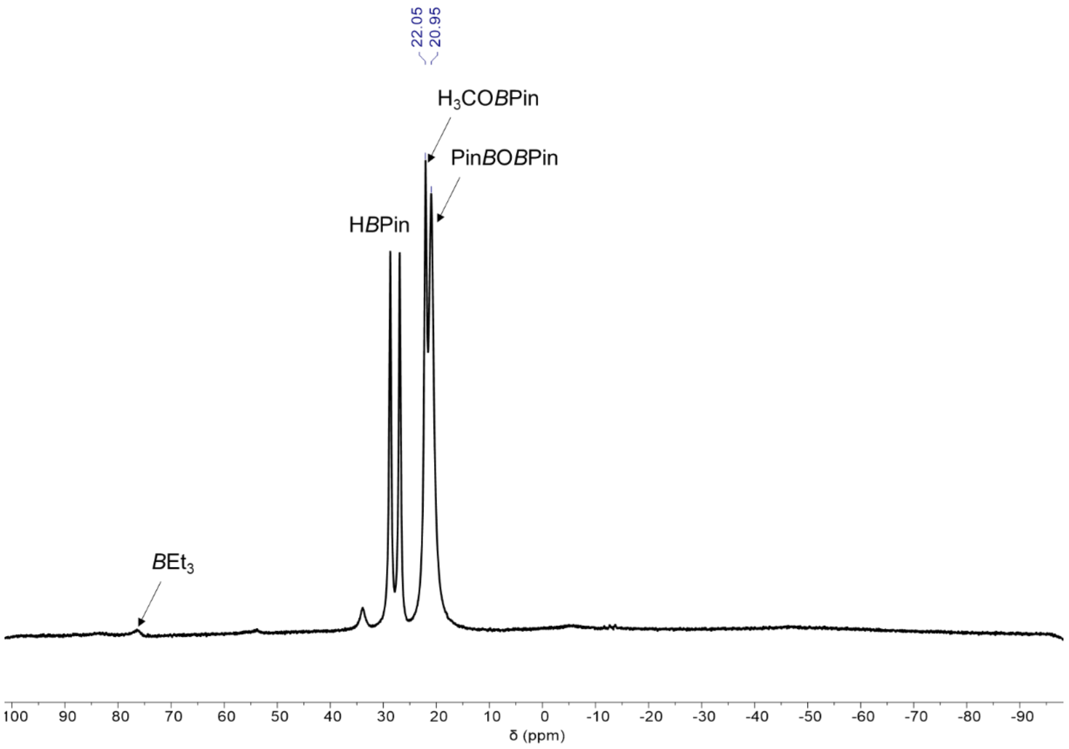

Figure S52 ^{11}B NMR spectrum (96 MHz, THF-d_8) of the hydroboration of CO_2 (1 bar) with HBPin catalyzed by 1 mol% of complex **3b**/2 $KHBEt_3$, at 40 ºC, for 16 h.

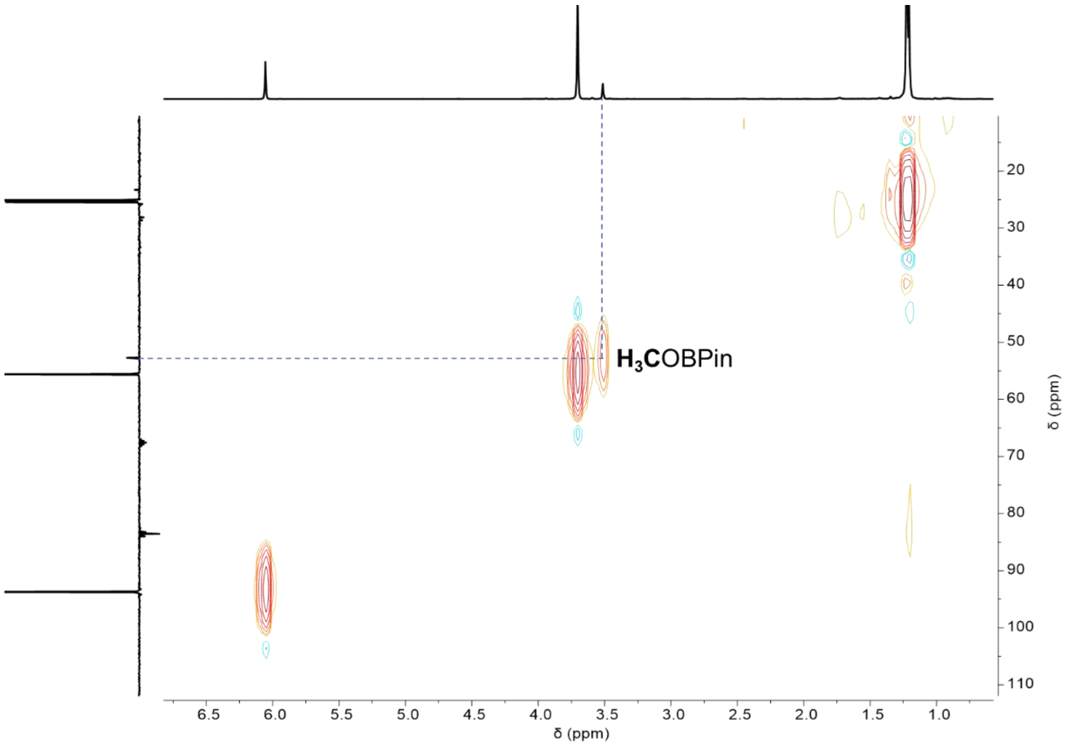

Figure S53 ^{1}H-^{13}C HSQC spectrum of the hydroboration of CO_2 (1 bar) with HBPin catalyzed by 1 mol% of complex **3b**/2 $KHBEt_3$, at 40 ºC, for 16 h.

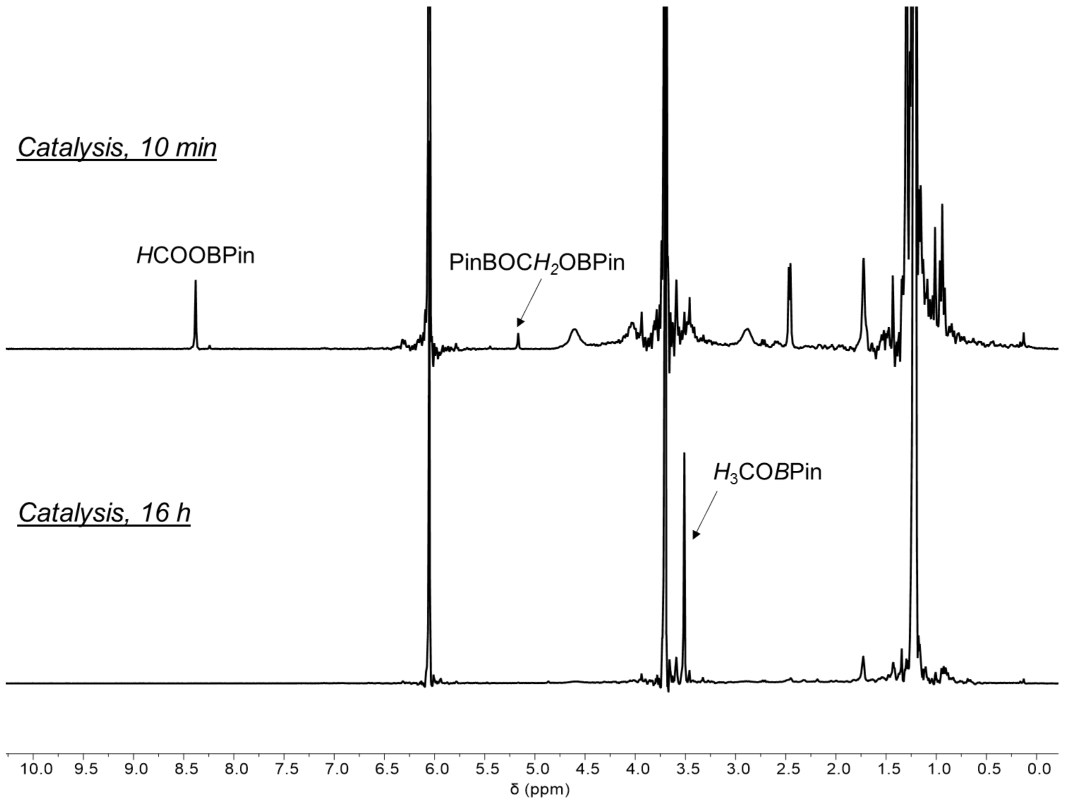

Figure S54 Stacking of the ^{1}H NMR spectra (300 MHz, THF-d_8) of the hydroboration of CO_2 (1 bar) with HBPin catalyzed by 1 mol% of complex **3b**/2 $KHBEt_3$, at 40 °C, for 10 min (top) and 16 h (bottom).

Table S3 Comparison between observed and reported ^{1}H and ^{11}B NMR (300 MHz, THF-d_8) resonances (in ppm) of the CO_2 hydroboration products.

Compound	^{1}H NMR (this work)	^{1}H NMR (literature)	^{11}B NMR (this work)	^{11}B NMR (literature)
HCOOBPin	8.38 (s, 1H, C*H*) [a]	8.29 (s, 1H, C*H*) [b]	*n.d.* [d]	21.7 [b]
PinBOCH$_2$OBPin	5.17 (s, 2H, C*H*$_2$) [a]	5.30 (s, 2H, C*H*$_2$) [c]	*n.d.* [d]	*n.a.* [e]
H$_3$COBPin	3.51 (s, 3H, C*H*$_3$), 1.21 (s, 12H, B*Pin*)	3.50 (s, 3H, C*H*$_3$), 1.16 (s, 12H, B*Pin*) [b]	22.1	22.0 [b]
PinBOBPin	1.22 (s, 24H, B*Pin*)	1.18 (s, 12H, B*Pin*) [b]	21.0	21.2 [b]

[a] The methyl protons of the BPin resonance were not determined since the product was only observed during the kinetic run was thus overlapped with those of HBPin. [b] Reference 29 (reference 14 in the article). [c] Reference 30 (reference 18c in the article). [d] Not determined since the product was not observed at the end of the catalytic reactions and no intermediate ^{11}B NMR spectra were collected during the kinetic run. [e] Data not available in the literature.

Literature comparison of catalytic results

Table S4 Summary of the catalysts and respective catalytic results used in the hydroboration of CO_2 which have selectively yielded methanol-level products.

$$CO_2 + 3\ HBPin \xrightarrow{cat.} H_3COBPin + PinBOBPin$$

Reference	Metal(%cat)	P_{CO2} (bar)	solvent	T (ºC)	t (h)	Yield (%)	TOF (h^{-1})
Angew. Chem. Int. Ed. **2012**, *51*, 11343–11345	Ru(5.6)	1	CD_2Cl_2	50	96	75	0.1
J. Am. Chem. Soc. **2013**, *135*, 9326−9329	-(1)[a]	2	C_6D_6	70	3	60	20
Chem. Sci. **2014**, *5*, 2826–2830	Mg(10)	1	THF-d_8	60	96	100	0.001
Inorg. Chem. **2015**, *54*, 7506−7515	Mo(0.1)	1	C_6D_6	90	8	97	121
Chem. Commun. **2016**, *52*, 13155−13158	Mg(10)	1	THF	25	3	100	3.3
ACS Catal. **2016**, *6*, 4526–4535	-(2.5)[a]	1	THF	90	16	100	2.5
Dalton Trans. **2017**, *46*, 6183–6186	Zn(10)	1	THF-d_8	60	16	100	0.6
Dalton Trans. **2018**, *47*, 8199–8203	Co(1)	1	THF-d_8	50	16	4	<0.001
Nat. Commun. **2018**, *9*, 4521–4530	Mn(0.036)	1	Neat	100	14	96	190
ACS Catal. **2019**, *9*, 301−314	Ni or Pd(1)	1	C_6D_6	RT	"days"	36	<1
Dalton Trans. **2020**, *49*, 2776–2780	Mg(5)	1	Neat	100	15	96	1.3
ChemCatChem, **2020**, *12*, 4625–4631	Mn(1)	1	THF-d_8	60	24	17	0.7
Inorg. Chem. **2021**, *60*, 4569−4577	Al(10)	1	C_6D_6	110	48	29	0.1
Chem. Eur. J. **2023**, e202303380	-(3)[a]	4	C_6D_6	60	144	100	0.2
This work	**Zn(1)**	**1**	**THF-d_8**	**60**	**16**	**86**	**5.4**

[a] Catalyst based on a frustrated Lewis pair metal-free system.

References

1 Juliá, S.; Sala, P.; Mazo, J. D.; Sancho, M.; Ochoa, C.; Elguero, J.; Fayet, J.-P.; Vertut, M.-C. *N*-polyazolylmethanes. 1. Synthesis and nmr study of *N,N′*-diazolylmethanes. *J. Heterocyclic Chem.* **1982**, *19*, 1141–1145.

2 Reedijk, J.; Verbiest, J. Coordination Compounds Derived from Transition Metal Salts and Bis(3,5-dimethylpyrazolyl)methane. *Transition Met. Chem.* **1979**, *4*, 239–243.

3 Zweifel, G.; Ayyangar, N. R.; Brown, H. C. Hydroboration. XVII. An Examination of Several Representative Dialkylboranes as Selective Hydroborating Agents. *J. Am. Chem. Soc.* **1963**, *85*, 2072–2075.

4 (a) Parks, D. J.; Piers, W. E.; Yap, G. P. A. Synthesis, Properties, and Hydroboration Activity of the Highly Electrophilic Borane Bis(pentafluorophenyl)borane, $HB(C_6F_5)_2$. *Organometallics* **1998**, *17*, 5492–5503. (b) Longobardi, L. E.; Johnstone, T. C.; Falconer, R. L.; Russell, C. A.; Stephan, D. W. Hydroboration of Phosphaalkynes by $HB(C_6F_5)_2$. *Chem. Eur. J.* **2016**, *22*, 12665–12669.

5 Knights, E. F.; Brown, H. C. 9-Borabicyclo[3.3.1]nonane as a convenient selective hydroborating agent. *J. Am. Chem. Soc.* **1968**, *90*, 5281–5283.

6 Drover, M. W.; Schafer, L. L.; Love, J. A. Capturing $HBCy_2$: Using N,O-Chelated Complexes of Rhodium(I) and Iridium(I) for Chemoselective Hydroboration. *Angew. Chem. Int. Ed.* **2016**, *55*, 3181–3186.

7 Fulmer, G. R.; Miller, A. J. M.; Sherden, N. H.; Gottlieb, H. E.; Nudelman, A.; Stoltz, B. M.; Bercaw, J. E.; Goldberg, K. I. NMR Chemical Shifts of Trace Impurities: Common Laboratory Solvents, Organics, and Gases in Deuterated Solvents Relevant to the Organometallic Chemist. *Organometallics* **2010**, *29*, 2176–2179.

8 Miller, C. C. The Stokes-Einstein law for diffusion in solution. *Proc. R. Soc. Lond. A* **1924**, *106*, 724–749

9 SMART Software for the CCD Detector System Version 5.625, Bruker AXS Inc., Madison, WI, USA, 2001.

10 SAINT Software for the CCD Detector System, Version 7.03, Bruker AXS Inc., Madison, WI, USA, 2004.

11 Sheldrick, G. M. SADABS, *Program for Empirical Absorption Correction*, University of Göttingen, Göttingen, 1996.

12 Burla, M. C.; Caliandro, R.; Carrozzini, B.; Cascarano, G. L.; Cuocci, C.; Giacovazzo, C.; Mallamo, M.; Mazzone, A.; Polidori, G. Crystal structure determination and refinement via SIR2014. *J. Appl. Cryst.* **2015**, *48*, 306–309.

13 (a) Sheldrick, G. M. Crystal structure refinement with SHELXL. *Acta Crystallogr., Sect. C: Struct. Chem.* **2015**, *71*, 3–8. (b) Hübschle, C. B.; Sheldrick, G. M.; Dittrich, B. ShelXle: a Qt graphical user interface for SHELXL. *J. Appl. Crystallogr.*, 2011, **44**, 1281–1284.

14 (a) Farrugia, L. J. WinGX suite for small-molecule single-crystal crystallography. *J. Appl. Crystallogr.* **1999**, *32*, 837–838. (b) Farrugia, L. J. WinGX and ORTEP for Windows: an update. *J. Appl. Crystallogr.* **2012**, *45*, 849–854.

15 (a) Spek, A. L. *PLATON – A Multipurpose Crystallographic Tool*, Utrecht University, Utrecht, The Netherlands, 1998. (b) Spek, A. L. Structure validation in chemical crystallography. *Acta Crystallogr., Sect. D: Biol. Crystallogr.* **2009**, *65*, 148–155.

16 Macrae, C. F.; Sovago, I.; Cottrell, S. J.; Galek, P. T. A.; McCabe, P.; Pidcock, E.; Platings, M.; Shields, G. P.; Stevens, J. S.; Towler, M.; Wood, P. A. Mercury 4.0: from visualization to analysis, design and prediction. *J. Appl. Cryst.* **2020**, *53*, 226–235.

17 GAUSSIAN 09, Revision A.01, Frisch, M. J.; Trucks, G. W.; Schlegel, H. B.; Scuseria, G. E.; Robb, M. A.; Cheeseman, J. R.; Scalmani, G.; Barone, V.; Mennucci, B.; Petersson, G. A.; Nakatsuji, H.; Caricato, M.; Li, X.; Hratchian, H. P.; Izmaylov, A. F.; Bloino, J.; Zheng, G.; Sonnenberg, J. L.; Hada, M.; Ehara, M.; Toyota, K.; Fukuda, R.; Hasegawa, J.; Ishida, M.; Nakajima, T.; Honda, Y.; Kitao, O.; Nakai, H.; Vreven, T.; Montgomery, Jr., J. A.; Peralta, J. E.; Ogliaro, F.; Bearpark, M.; Heyd, J. J.; Brothers, E.; Kudin, K. N.; Staroverov, V. N.; Kobayashi, R.; Normand, J.; Raghavachari, K.; Rendell, A.; Burant, J. C.; Iyengar, S. S.; Tomasi, J.; Cossi, M.; Rega, N.; Millam, J. M.; Klene, M.; Knox, J. E.; Cross, J. B.; Bakken, V.; Adamo, C.; Jaramillo, J.; Gomperts, R.; Stratmann, R. E.; Yazyev, O.; Austin, A. J.; Cammi, R.; Pomelli, C.; Ochterski, J. W.; Martin, R. L.; Morokuma, K.; Zakrzewski, V. G.; Voth, G. A.; Salvador, P.; Dannenberg, J. J.; Dapprich, S.; Daniels, A. D.; Farkas, Ö.; Foresman, J. B.; Ortiz, J. V.; Cioslowski, J.; Fox, D. J. Gaussian, Inc., Wallingford CT, 2009.

18 Hehre, W. J.; Radom, L.; Schleyer, P. v.R.; Pople, J. A. Ab Initio Molecular Orbital Theory, John Wiley & Sons, NY, 1986.

19 Parr, R. G.; Yang, W. Density Functional Theory of Atoms and Molecules; Oxford University Press: New York, 1989.

20 (a) Perdew, J. P.; Burke, K.; Ernzerhof, M. Generalized Gradient Approximation Made Simple. *Phys. Rev. Lett.* **1996**, *77*, 3865–3868; (b) Perdew, J. P.; Burke, K.; Ernzerhof, M. Generalized Gradient Approximation Made Simple. *Phys. Rev. Lett.* **1997**, *78*, 1396–1396. (c) Perdew, J. P. Density-functional approximation for the correlation energy of the inhomogeneous electron gas. *Phys. Rev. B* **1986**, *33*, 8822–8824.

21 (a) Haeusermann, U.; Dolg, M.; Stoll, H.; Preuss, H.; Schwerdtfeger, P.; Pitzer, R. M. Accuracy of energy-adjusted quasirelativistic ab initio pseudopotentials. *Mol. Phys.* **1993**, *78*, 1211–1224. (b) Kuechle, W.; Dolg, M.; Stoll, H.; Preuss, H. Energy-adjusted pseudopotentials for the actinides. Parameter sets and test calculations for thorium and thorium monoxide. *J. Chem. Phys.* **1994**, *100*, 7535–7542. (c) Leininger, T.; Nicklass, A.; Stoll, H.; Dolg, M.; Schwerdtfeger, P. The accuracy of the pseudopotential approximation. II. A comparison of various core sizes for indium pseudopotentials in calculations for spectroscopic constants of InH, InF, and InCl. *J. Chem. Phys.* **1996**, *105*, 1052–1059.

22 Ehlers, A. W.; Böhme, M.; Dapprich, S.; Gobbi, A.; Höllwarth, A.; Jonas, V.; Köhler, K. F.; Stegmann, R.; Veldkamp, A.; Frenking, G. A set of f-polarization functions for pseudo-potential basis sets of the transition metals Sc−Cu, Y−Ag and La−Au. *Chem. Phys. Lett.* **1993**, *208*, 111–114.

23 (a) Ditchfield, R.; Hehre, W. J.; Pople, J. A. Self-Consistent Molecular-Orbital Methods. IX. An Extended Gaussian-Type Basis for Molecular-Orbital Studies of Organic Molecules. *J. Chem. Phys.* **1971**, *54*, 724–728. (b) Hehre, W. J.; Ditchfield, R.; Pople, J. A. Self-Consistent Molecular Orbital Methods. 12. Further extensions of Gaussian-type basis sets for use in molecular-orbital studies of organic-molecules. *J. Chem. Phys.* **1972**, *56*, 2257–2261. (c) Hariharan, P. C.; Pople, J. A. Accuracy of AH equilibrium geometries by single determinant molecular-orbital theory. *Mol. Phys.* **1974**, *27*, 209–214. (d) Gordon, M. S. The isomers of silacyclopropane. *Chem. Phys. Lett.* **1980**, *76*, 163–168. (e) Hariharan, P. C.; Pople, J. A. Influence of polarization functions on molecular-orbital hydrogenation energies. *Theor. Chim. Acta* **1973**, *28*, 213–222.

24 (a) McClean, A. D.; Chandler, G. S. Contracted Gaussian basis sets for molecular calculations. I. Second row atoms, *Z*=11-18 *J. Chem. Phys.* **1980**, *72*, 5639–5648. (b) Krishnan, R.; Binkley, J. S.; Seeger, R.; Pople, J. A. Self-consistent molecular orbital methods. XX. A basis set for correlated wave functions. *J. Chem. Phys.* **1980**, *72*, 650–654. (c) Wachters, A. J. H. Gaussian Basis Set for Molecular Wavefunctions Containing Third-Row Atoms. *J. Chem. Phys.* **1970**, *52*, 1033–1036. (d) Hay, P. J.

Gaussian basis sets for molecular calculations – representation of 3D orbitals in transition-metal atoms. *J. Chem. Phys.* **1977**, *66*, 4377–4384. (e) Raghavachari, K.; Trucks, G. W. Highly correlated systems: Excitation energies of first row transition metals Sc-Cu. *J. Chem. Phys.* **1989**, *91*, 1062–1065. (f) Binning Jr., R. C.; Curtiss, L. A. Compact contracted basis-sets for 3rd-row atoms - Ga-Kr. *J. Comp. Chem.* **1990**, *11*, 1206–1216. (g) McGrath, M. P.; Radom, L. Extension of Gaussian-1 (G1) theory to bromine-containing molecules. *J. Chem. Phys.* **1991**, *94*, 511–516. (h) Curtiss, L. A.; McGrath, M. P.; Blaudeau, J.-P.; Davis, N. E.; Binning Jr., R. C.; Radom, L. Extension of Gaussian-2 theory to molecules containing third-row atoms Ga-Kr.. *J. Chem. Phys.* **1995**, *103*, 6104–6113. (i) Clark, T.; Chandrasekhar, J.; Spitznagel, G. W.; Schleyer, P. v. R. Efficient diffuse function-augmented basis-sets for anion calculations. 3. The 3-21+G basis set for 1st-row elements, Li-F. *J. Comp. Chem.* **1983**, *4*, 294–301. (j) Frisch, M. J.; Pople, J. A.; Binkley, J. S. Self-Consistent Molecular Orbital Methods. 25. Supplementary Functions for Gaussian Basis Sets. *J. Chem. Phys.* **1984**, *80*, 3265–3269.

25 (a) Cancès, M. T.; Mennucci, B.; Tomasi, J. A new integral equation formalism for the polarizable continuum model: Theoretical background and applications to isotropic and anistropic dielectrics. *J. Chem. Phys.* **1997**, *107*, 3032–3041. (b) Cossi, M.; Barone, V.; Mennucci, B.; Tomasi, J. Ab initio study of ionic solutions by a polarizable continuum dielectric model. *Chem. Phys. Lett.* **1998**, *286*, 253–260. (c) Mennucci, B.; Tomasi, J. Continuum solvation models: A new approach to the problem of solute's charge distribution and cavity boundaries. *J. Chem. Phys.* **1997**, *106*, 5151–5158. (d) Tomasi, J.; Mennucci, B.; Cammi, R. Quantum mechanical continuum solvation models. *Chem. Rev.* **2005**, *105*, 2999–3094.

26 Marenich, A. V.; Cramer, C. J.; Truhlar, D. G. Universal solvation model based on solute electron density and a continuum model of the solvent defined by the bulk dielectric constant and atomic surface tensions. *J. Phys. Chem. B*, **2009**, *113*, 6378–6396.

27 Grimme, S.; Antony, J.; Ehrlich, S.; Krieg, H. A consistent and accurate ab initio parameterization of density functional dispersion correction (DFT-D) for the 94 elements H-Pu. *J. Chem. Phys.* **2010**, *132*, 154104.

28 (a) Becke, A. D.; Johnson, E. R. A density-functional model of the dispersion interaction. *J. Chem. Phys.* **2005**, *122*, 154101. (b) Johnson, E. R.; Becke, A. D. A post-Hartree-Fock model of intermolecular interactions. *J. Chem. Phys.* **2005**, *123*, 24101. (c) Johnson, E. R.; Becke, A. D. A post-Hartree-Fock model of intermolecular interactions: Inclusion of higher-order corrections. *J. Chem. Phys.* **2006**, *124*, 174104.

29 Tamang, S. R.; Findlater, M. Cobalt catalysed reduction of CO_2 via Hydroboration. *Dalton Trans.* **2018**, *47*, 8199–8203.

30 Bontemps, S.; Vendier, L.; Sabo-Etienne, S. Ruthenium-Catalyzed Reduction of Carbon Dioxide to Formaldehyde. *J. Am. Chem. Soc.* **2014**, *136*, 4419−4425.
